# Supplementary material for: Efficacy and moderators of mindfulness-based cognitive therapy (MBCT) in ‘Difficult to Treat’ depression: protocol for a systematic review and individual participant data meta-analysis of randomised controlled trials
Source: BMJ Open. 2025 Dec 8;15(12):e106350. doi: 10.1136/bmjopen-2025-106350 (PMC12719901; doi:10.1136/bmjopen-2025-106350)
Supplement: Supplementary data [file bmjopen-15-12-s001.pdf]

APPENDIX

Search Terms

Database	Search Term
Web of Science	(“MDE” OR “major depressive episode” OR “MDD” OR “major depressive disorder” OR “major depression” OR “depression” OR “dysthymia” OR “dysthymic”) AND (“chronic” OR “persistent” OR “treatment-resistant” OR “non-responders” OR “non-response” OR “non-remitted” OR “dysthymia” OR “dysthymic”) AND (“MBCT” OR "mindfulness-based cognitive therapy") AND (“randomized controlled trial” OR “randomised controlled trial” OR “randomized-controlled trial” OR “randomised-controlled trial” OR “RCT” OR “randomized” or “randomised” OR “randomly allocated” OR “randomly assigned”)
Scopus	(“MDE” OR “major depressive episode” OR “MDD” OR “major depressive disorder” OR “major depression” OR “depression” OR “dysthymia” OR “dysthymic”) AND (“chronic” OR “persistent” OR “treatment-resistant” OR “non-responders” OR “non-response” OR “non-remitted” OR “dysthymia” OR “dysthymic”) AND (“MBCT” OR "mindfulness-based cognitive therapy") AND (“randomized controlled trial” OR “randomised controlled trial” OR “randomized-controlled trial” OR “randomised-controlled trial” OR “RCT” OR “randomized” or “randomised” OR “randomly allocated” OR “randomly assigned”)
PubMed	(“MDE” OR “major depressive episode” OR “MDD” OR “major depressive disorder” OR “major depression” OR “depression” OR “dysthymia” OR “dysthymic”) AND (“chronic” OR “persistent” OR “treatment-resistant” OR “non-responders” OR “non-response” OR “non-remitted” OR “dysthymia” OR “dysthymic”) AND (“MBCT” OR "mindfulness-based cognitive therapy") AND (“randomized controlled trial” OR “randomised controlled trial” OR “randomized-controlled trial” OR “randomised-controlled trial” OR “RCT” OR “randomized” or “randomised” OR “randomly allocated” OR “randomly assigned”)
PsycInfo	(“MDE” OR “major depressive episode” OR “MDD” OR “major depressive disorder” OR “major depression” OR “depression” OR “dysthymia” OR “dysthymic”) **AND** (“chronic” OR “persistent” OR “treatment-resistant” OR “non-responders” OR “non-response” OR “non-remitted” OR “dysthymia” OR “dysthymic”) **AND** (“MBCT” OR "mindfulness-based cognitive therapy") **AND** (“randomized controlled trial” OR “randomised controlled trial” OR “randomized-controlled trial” OR “randomised-controlled trial” OR “RCT” OR “randomized” or “randomised” OR “randomly allocated” OR “randomly assigned”)
Embase	(‘MDE’ OR ‘major depressive episode’ OR ‘MDD’ OR ‘major depressive disorder’ OR ‘major depression’ OR ‘depression’ OR ‘dysthymia’ OR ‘dysthymic’) **AND** (‘chronic’ OR ‘persistent’ OR ‘treatment-resistant’ OR ‘non-responders’ OR ‘non-response’ OR ‘non-remitted’ OR ‘dysthymia’ OR ‘dysthymic’) **AND** (‘MBCT’ OR ‘mindfulness-based cognitive therapy’) **AND** (‘randomized controlled trial’ OR ‘randomised controlled trial’ OR ‘randomized-controlled trial’ OR ‘randomised-controlled trial’ OR ‘RCT’ OR ‘randomized’ or ‘randomised’ OR ‘randomly allocated’ OR ‘randomly assigned’)

Barnhofer T, *et al. BMJ Open* 2025; 15:e106350. doi: 10.1136/bmjopen-2025-106350

Cochrane Controlled Trials	(“MDE” OR “major depressive episode” OR “MDD” OR “major depressive disorder” OR “major depression” OR “depression” OR “dysthymia” OR “dysthymic”) AND (“chronic” OR “persistent” OR “treatment-resistant” OR “non-responders” OR “non-response” OR “non-remitted” OR “dysthymia” OR “dysthymic”) AND (“MBCT” OR "mindfulness-based cognitive therapy") AND (“randomized controlled trial” OR “randomised controlled trial” OR “randomized-controlled trial” OR “randomised-controlled trial” OR “RCT” OR “randomized” or “randomised” OR “randomly allocated” OR “randomly assigned”)

Barnhofer T, *et al. BMJ Open* 2025; 15:e106350. doi: 10.1136/bmjopen-2025-106350
