# Supplementary material for: Efficacy and moderators of mindfulness-based cognitive therapy (MBCT) in ‘Difficult to Treat’ depression: protocol for a systematic review and individual participant data meta-analysis of randomised controlled trials
Source: BMJ Open. 2025 Dec 8;15(12):e106350. doi: 10.1136/bmjopen-2025-106350 (PMC12719901; doi:10.1136/bmjopen-2025-106350)
Supplement: Author's manuscript [file bmjopen-2025-106350.draft_revisions.pdf]

BMJ Open

BMJ Open is committed to open peer review. As part of this commitment we make the peer review history of every article we publish publicly available.

When an article is published we post the peer reviewers' comments and the authors' responses online. We also post the versions of the paper that were used during peer review. These are the versions that the peer review comments apply to.

The versions of the paper that follow are the versions that were submitted during the peer review process. They are not the versions of record or the final published versions. They should not be cited or distributed as the published version of this manuscript.

BMJ Open is an open access journal and the full, final, typeset and author-corrected version of record of the manuscript is available on our site with no access controls, subscription charges or pay-per-view fees (http://bmjopen.bmj.com).

If you have any questions on BMJ Open's open peer review process please email info.bmjopen@bmj.com

BMJ Open

Efficacy and Moderators of Mindfulness-Based Cognitive Therapy (MBCT) in 'Difficult to Treat' Depression: Protocol for a Systematic Review and Individual Patient Data Meta-Analysis of Randomised Controlled Trials

Journal:	*BMJ Open*
Manuscript ID	bmjopen-2025-106350
Article Type:	Protocol
Date Submitted by the Author:	10-Jun-2025

Complete List of Authors:	Barnhofer, Thorsten; University of Surrey,<br>Niemi, Maria; Karolinska Institutet, Department of Global Public Health<br>Michalak, Johannes; Witten/Herdecke University, Department of Psychology and Psychotherapy<br>Velana, Maria; Witten/Herdecke University, Department of Psychology and Psychotherapy<br>Williams, J Mark; University of Oxford, Department of Psychiatry<br>Chiesa, Alberto; Università di Bologna<br>Eisendrath, Stuart; University of California at San Francisco<br>Delucchi, Kevin; University of California at San Francisco<br>Segal, Zindel; University of Toronto - Scarborough, Graduate Department of Psychological Clinical Science<br>Cladder-Micus, Mira; Radboud University Nijmegen<br>Speckens, Anne; Radboudumc Instituut voor Wetenschappelijk Onderwijs en Opleidingen, Psychiatry<br>Foroughi, Ali Akbar; Kermanshah University of Medical Sciences (KUMS), Department of Clinical Psychology<br>García-Toro, M; Research Network on Chronicity Primary Care and Health Promotion; University of the Balearic Islands; Health Research Institute of the Balearic Islands; University of the Balearic Islands, Department of Medicine<br>Montero-Marin, Jesus; Research & Innovation Unit Parc Sanitari Sant Joan de Déu; University of Oxford, Department of Psychiatry; Consortium for Biomedical Research in Epidemiology and Public Health<br>dunn, barney; University of Exeter<br>Strauss, Clara; Sussex Partnership NHS Foundation Trust; University of Sussex<br>Ruths, Florian; South London and Maudsley NHS Foundation Trust<br>Ryan, Mary; Royal College of Psychiatrists<br>Harrer, Mathias; Technical University of Munich, Department of Psychiatry and Psychotherapy; World Health Organization, Department of Clinical, Neuro and Developmental Psychology; Vrije Universiteit

Keywords:	Meta-Analysis, Depression & mood disorders < PSYCHIATRY, Mindfulness

For peer review only - http://bmjopen.bmj.com/site/about/guidelines.xhtml

Title

Efficacy and Moderators of Mindfulness-Based Cognitive Therapy (MBCT) in 'Difficult to Treat' Depression: Protocol for a Systematic Review and Individual Patient Data Meta-Analysis of Randomised Controlled Trials

Running Title

MBCT in 'Difficult to Treat' Depression

Authors and Affiliations

Thorsten Barnhofer [1], Maria Niemi [2, *], Johannes Michalak [3], Maria Velana [3], J. Mark G. Williams [4], Alberto Chiesa [5], Stuart Eisendrath [6], Kevin Delucchi [6], Zindel V. Segal [7], Mira Cladder-Micus [8], Anne Speckens [9], Ali Akbar Foroughi [10], Mauro Garcia-Toro [11, 12, 13, 14], Jesus Montero-Marin [15, 4, 16], Barnaby D. Dunn [17], Clara Strauss [18, 19], Florian A. Ruths [20], Mary Ryan [21], Mathias Harrer [22, 23, 24]

[1] School of Psychology, University of Surrey, Guildford, UK, t.barnhofer@surrey.ac.uk

[2] Department of Global Public Health, Karolinska Institutet, Stockholm, Sweden, maria.niemi@ki.se

[3] Department of Psychology and Psychotherapy, Witten/Herdecke University, Witten, Germany, Johannes.Michalak@uni-wh.de, Maria.Velana@uni-wh.de ,

[4] Department of Psychiatry, University of Oxford, Oxford, UK, mark.williams@psych.ox.ac.uk, jesus.monteromarin@psych.ox.ac.uk

[5] Institute of Psychiatry, Università di Bologna, Bologna, Italy, chiesa@scuola-spc.it

[6] Weill Institute for Neurosciences, University of California at San Francisco, San Francisco, USA, stuart.eisendrath@ucsf.edu, kevin.delucchi@ucsf.edu

[7] Graduate Department of Psychological Clinical Science, University of Toronto - Scarborough, Toronto, Canada, zindel.segal@utoronto.ca

For peer review only

[8] Behavioural Science Institute, Radboud University Nijmegen, Nijmegen, The Netherlands, Mira.Cladder-Micus@radboudumc.nl

[9] Department of Psychiatry, Radboudumc, Nijmegen, The Netherlands, anne.speckens@radboudumc.nl

[10] Department of Clinical Psychology, Kermanshah University of Medical Sciences (KUMS), Hermanshah, Iran, foroughi_2002@yahoo.com

[11] Research Network on Chronicity, Primary Care and Health Promotion (RICAPPS), Carlos III Health Institute, Madrid, Spain

[12] University Institute of Health Science Research (IUNICS), University of the Balearic Islands, Spain, mauro.garcia@uib.es

[13] Health Research Institute of the Balearic Islands (IdISBa), Palma, Spain

[14] Department of Medicine, University of the Balearic Islands, Palma, Spain

[15] Research & Innovation Unit, Parc Sanitari Sant Joan de Déu, Sant Boi de Llobregat, Barcelona, Spain

[16] Consortium for Biomedical Research in Epidemiology and Public Health (CIBER Epidemiology and Public Health-CIBERESP), Madrid, Spain

[17] Mood Disorders Centre, University of Exeter, Exeter, UK, b.d.dunn@exeter.ac.uk

[18] Sussex Partnership NHS Foundation Trust, Hove, UK, clara.strauss@nhs.net

[19] Sussex Mental Health Research Centre, University of Sussex, Brighton, UK

[20] South London and Maudsley NHS Foundation Trust, London, UK, Florian.Ruths@slam.nhs.uk

[21] Royal College of Psychiatrists, London, UK, mary@highbag.co.uk

[22] Department of Psychiatry and Psychotherapy, School of Medicine and Health, Technical University of Munich, Munich, Germany

[23] Department of Clinical, Neuro and Developmental Psychology, World Health Organization

For peer review only - http://bmjopen.bmj.com/site/about/guidelines.xhtml

[24] Collaborating Center for Research and Dissemination of Psychological Interventions, Amsterdam, Public Health Research Institute, Vrije Universiteit, Amsterdam, The Netherlands, mathias.harrer@tum.de

* Corresponding author:

Associate Professor Maria Niemi, Department of Global Public Health, Karolinska Institutet, Stockholm, Sweden, maria.niemi@ki.se

For peer review only

Word Count

Abstract: 300

Main text: 4110

Keywords

Major Depression, Difficult-to-Treat, Mindfulness-Based Cognitive Therapy, Independent Patient Data, Meta-Analysis

For peer review only - http://bmjopen.bmj.com/site/about/guidelines.xhtml

ABSTRACT

Introduction: About 30% of depressed patients suffer from a protracted course in which the disorder continues to cause significant burden despite treatment efforts. While originally developed for relapse prevention, Mindfulness-Based Cognitive Therapy (MBCT) has increasingly been investigated in depressed patients with such 'difficult-to-treat' courses. This is a protocol for an individual participant data (IPD) meta-analysis aiming to determine efficacy and potential moderators of MBCT treatment effects in this group based on evidence from randomised controlled trials.

Methods and analysis: Systematic searches in PubMed, Web of Science, Scopus, PsycINFO, EMBASE, and the Cochrane Controlled Trials Register for randomised controlled trials were completed on 17 June 2024. Authors of identified studies have contributed IPD and data extractions have been completed. An update search will be conducted immediately before the start of data analyses. We will investigate the following outcomes: (a) self-reported and observer-reported severity of depression symptomatology, (b) remission, (c) clinically meaningful improvement and deterioration. One-stage and two-stage IPD meta-analyses will be conducted with one-stage models using the observed IPD from all studies simultaneously as the primary approach. One-stage IPD models will include stratified study intercepts and error terms as well as random effects to capture between-study heterogeneity. Moderator analyses will test treatment-covariate interactions for both individual patient level and study level characteristics.

Ethics and dissemination: The results will inform understanding of the use of MBCT in patients with current 'difficult-to-treat' depression and will contribute to arguments in favour of or against implementing MBCT as a treatment for this group. They will be published in a peer reviewed journal and made available to stakeholders in accessible formats. No local ethical review was necessary following consultation with the Ethics and Governance Board

For peer review only - http://bmjopen.bmj.com/site/about/guidelines.xhtml

of the University of Surrey. Guidance on patient data storage and management will be adhered to throughout.

PROSPERO registration number: CRD42022332039

For peer review only

For peer review only - http://bmjopen.bmj.com/site/about/guidelines.xhtml

STRENGTHS AND LIMITATIONS OF THIS STUDY

- The chosen approach using IPD has several advantages over conventional meta-analyses including the possibility to assess whether individual participant level characteristics influence treatment effects with more specificity and power
- Limitations of the approach include the fact that inconsistencies across studies regarding the format and type of variables reported may restrict our ability to investigate moderators of treatment effects
- Transformation of outcome measures to a common metric across all studies may come with a small loss of information.

For peer review only

INTRODUCTION

Major Depressive Disorder (MDD) is highly prevalent, debilitating [1] , and in about 30% of patients takes a recurrent or chronic course in which established treatments fail to bring sustained remission [2] . Recently framed under the broader heuristic of difficult-to-treat depression (DTD) [3, 4] , such courses are associated with continuing functional impairment, reduced quality of life, and significantly increased risk for chronic physical and neurodegenerative disorders [5, 6] .

While treatment approaches for DTD have traditionally been dominated by pharmacological strategies [7] , it is now increasingly acknowledged that effective management requires a broader emphasis on working with symptoms, maximising function and minimizing burden [4] . Psychological interventions have an important contribution to make in this context [8] and it has been suggested that Mindfulness-Based Cognitive Therapy (MBCT [9]), an eight-week treatment that combines elements of cognitive therapy with mindfulness meditation, may be particularly suited for this purpose. Using a mental training approach, the intervention aims to provide patients with portable skills that enable them to disengage from habitual maladaptive responses and respond more adaptively to negative mood and stress.

MBCT was originally introduced for the prevention of relapse in patients who are currently in remission and has been proven to be effective for this purpose [10] , particularly in patients with highly recurrent courses (a use that is endorsed by treatment guidelines internationally). Early trials restricted the intervention to patients who were currently in full remission as it was assumed that intensive mindfulness practice may be too demanding for those who are suffering from current symptoms [11, 12] . However, as patients who are at high risk of relapse are likely to show residual symptoms between episodes, this restriction was later loosened, and analyses across studies for relapse prevention show that treatment

For peer review only - http://bmjopen.bmj.com/site/about/guidelines.xhtml

effects increase, rather than decrease, with level of residual symptoms [10] . From a theoretical perspective, this observation is in line with the view that mindfulness skills serve to buffer the effects of negative mood and stress [13, 14] , which implies that effects should become more visible under conditions of higher symptoms or stress.

Based on this reasoning, research has extended the use of MBCT to patients with current depression that has proven ‘difficult-to-treat’. Preliminary studies investigating MBCT in treatment-resistant and chronic depression were published in 2008 [15] and 2009 [16] and several definitive randomised controlled trials have been conducted since then [17–22] , suggesting that evidence has accumulated to a point where the use of MBCT as a treatment for patients with DTD can now be considered for guideline endorsement and wider implementation. It seems timely therefore to bring data from existing randomised controlled trials together and analyse outcome across studies.

For this purpose, we have pooled individual patient data (IPD) from these studies to conduct meta-analyses with one-stage random effects meta-analysis of independent patient data (IPD) as our primary approach given its advantages over conventional aggregate data meta-analyses [23–26] . The main aim of the analyses is to establish efficacy of MBCT for patients with DTD as compared to the control conditions utilised in the existing trials. Given the need for both effective symptom reduction and the maintenance of gains, we will aim to determine immediate effects at the end of the eight-week MBCT intervention (post-treatment) and effects in the longer-term after patients have finished the intervention and are free to continue engaging in mindfulness practice by themselves (follow-up).

An important advantage of IPD meta-analysis is that it allows to examine moderator or subgroup questions across the pooled sample and can thus serve to overcome limitations in sample size and power for answering these questions in the original studies [27] . Information about potential moderators of treatment effects, such as severity of depression at entry to

For peer review only - http://bmjopen.bmj.com/site/about/guidelines.xhtml

treatment, is important as it can potentially help with implementation by providing more precise information about relative indications or contraindications of the intervention. A further aim of this IPD meta-analysis is therefore to explore effects of potential individual patient level (e.g. sociodemographic and clinical characteristics) and study level (e.g. delivery format) moderators of treatment effects in the pooled data set.

As the clinical concept of DTD does not link to an objective way of assessment, the current meta-analysis will include studies that have used a range of conventional definitions falling under the wider concept of DTD, including selecting participants based on treatment non-response/remission, treatment resistance or the presence of chronic depression. Subgroup analyses will allow us to determine the extent to which effects found in the aggregated data generalise across these conventional categories.

METHODS

General approach

This IPD meta-analysis is registered on PROSPERO (registration number: CRD42022332039; date of registration in PROSPERO: 24 May 2022) and any key changes or amendments will be documented there. The protocol is registered retrospectively as searches to identify eligible papers were already conducted in 2024, with the last search having been performed on 17 June 2024 and an update search planned immediately before the start of data analysis. Corresponding authors of the selected studies were contacted and asked to provide raw data from their studies. The Preferred Reporting Items for Systematic Reviews and Meta-Analyses IPD statement [28] will be followed for the reporting of this study.

For peer review only - http://bmjopen.bmj.com/site/about/guidelines.xhtml

Eligibility criteria

Types of studies to be included

This IPD meta-analysis will include only peer-reviewed RCTs, published in English. There will be no restriction on the type of recruitment settings.

Type of intervention

Included studies will have investigated the effects of manualised mindfulness-based cognitive therapy (MBCT) alone or as an adjunct treatment to treatment-as-usual (TAU) [29]. MBCT is a psychological group-based intervention that combines intensive training in mindfulness meditation with elements of cognitive behavioural therapy (CBT) for depression. Mindfulness can be operationally defined as non-judgemental awareness of present moment experience [30]. As MBCT had been originally designed to prevent relapse into depression and not to reduce symptoms during an episode, we will accept studies that introduce minor changes to the manual (e.g. incorporating specific support for managing current depressive symptoms), as well as standard manualised MBCT, but not other types of mindfulness-based interventions, and include MBCT studies regardless of delivery format (e.g. physical group setting versus videoconference).

Types of comparators

This may include TAU for depression, including pharmacological and psychological treatments (e.g., CBT) or active controls. The latter will include established interventions as well as control interventions designed to mimic effects of mindfulness-based interventions in some or all aspects with the exception of the core element of mindfulness.

Participants

We will include studies that recruited adults with current Major Depressive Disorder (MDD) [31, 32] and studies which included mixed groups of patients with residual depressive symptoms or full episodes in the context of a chronic or treatment-resistant course of the

For peer review only - http://bmjopen.bmj.com/site/about/guidelines.xhtml

disorder. In the latter case, participants with only residual symptoms will be excluded from the primary analysis approach (one-stage meta-analysis). As DTD does not link to an objective way of assessment, we will opt for a maximally inclusive interpretation of the term and include any studies that selected patients based on previous treatment non-response/remission, treatment-resistance or chronicity. We will exclude studies with participants below the age of 18 and studies that have exclusively recruited participants over the age of 65 given that the factors involved in the onset, maintenance and recurrence of depression in these age groups can differ from those in adults within this age bracket [33, 34] .

Outcomes

The main type of outcome will be severity of depressive symptomatology as assessed using standard depression outcome measures. Standard depression outcome measures may include self-report measures and observer-rated measures. If both types of measures are available, we will investigate effects on both types of outcome measures. If several measures are available for a single study, preference will be given to the measure that was used as the primary outcome of the original study. To compare outcomes across studies using different outcome measures, we will transform scores from these measures into standardised scores using the common metric developed by Wahl et al. [35] . Where a measure is not part of the common metric approach, we will transform the measure into one that is part of the common metric using published conversion tables [36] , and then derive the common metric.

To facilitate clinical interpretation of results, we will derive dichotomous outcomes reflecting remission, improvement and deterioration. To apply a common rule for all studies, common metric scores will be converted to the PHQ-9 and dichotomous outcomes computed based on this measure. Following conventions for the PHQ-9 used in pragmatic trials and health services, remission will be defined as a score of less than 10 [37, 38], i.e. a shift from

For peer review only - http://bmjopen.bmj.com/site/about/guidelines.xhtml

"caseness" to a non-case score. Definition of clinically meaningful change will follow suggestions from recent trials in treatment-resistant depression, which has indicated a threshold of 6 or more points [39, 40] . We will therefore define clinically meaningful improvement as a reduction of 6 or more points and clinically meaningful deterioration as an increase of 6 or more points on the PHQ-9.

Secondary outcomes such as mindfulness, self-compassion and quality of life will be collected and may be used for future analysis.

Moderators

We will also investigate potential moderators of treatment response, i.e. factors that determine whether a person responds better to MBCT as compared to the control condition or vice versa. These will include potential moderators on the individual patient level (e.g. sociodemographic characteristics, history of childhood trauma, and clinical characteristics) and on the study level. We will include patient level characteristics in the analyses, if they are reported across a sufficient number of studies and datasets (see statistical analysis section for criteria).

Sociodemographic and clinical characteristics that we aim to investigate include age [41] , gender [42] , marital status [43] , education [44] , employment [45] , childhood adversity [46] , age at onset of depression [47] , number of previous episodes [48] , duration of current episode [49] , current suicidality [50] , current antidepressants [51] , past antidepressants and treatment-resistance to antidepressants [52] , co-morbid anxiety disorder, comorbid obsessive-compulsive disorder, co-morbid post-traumatic stress disorder, co-morbid substance abuse or dependence, co-morbid eating disorder, co-morbid personality disorder [41], and level of depressive symptoms at entry [53] . Study level characteristics will include type of delivery (remote versus face-to-face), type of control condition (TAU, active

For peer review only - http://bmjopen.bmj.com/site/about/guidelines.xhtml

control), type of DTD (non-response/remission, treatment-resistance, chronic depression) and study quality.

Timing of outcome assessments

As follow-up periods of the studies differ, we will group follow-up periods in the following categories: 8 to 10 weeks post-randomisation (post-treatment), 11 to 34 weeks post-randomisation (follow-up 1), and 35 to 60 weeks post-randomisation (follow-up 2).

Searches and study selection

We searched PubMed, Web of Science, Scopus, PsychINFO, EMBASE, and the Cochrane Controlled Trials Register using index and free terms, jointly with Boolean operators, on four tiers, namely: (1) depressive disorder, (2) non-response, treatment resistance and chronicity, (3) mindfulness-based cognitive therapy, and (4) randomised-controlled trial. The search terms used for the different databases are shown in the appendix.

We searched for any study published up until the day of the search, on 17 June 2024. There was no lower time limit. Initial screening of titles was performed by two independent reviewers (TB, JM), with the publication abstract being obtained if selected by at least one reviewer. Abstracts were screened by the same two independent reviewers, with disagreements about eligibility being referred to a third reviewer whose decision was final. An update search will be conducted immediately before the start of data analysis.

Risk of bias assessment

The validity of the studies will be assessed using the Risk of Bias tool by the Cochrane Collaboration (version 2) [54, 55] . Possible sources of bias assessed by the tool are (1) random sequence generation, (2) allocation concealment, (3) blinding of outcome assessors, (4) incomplete outcome data, (5) selective outcome reporting, and (6) other threats to validity including lack of compliance and different timing of outcome assessments. Data from the published papers of the trials will be evaluated by two independent assessors.

For peer review only

For peer review only - http://bmjopen.bmj.com/site/about/guidelines.xhtml

Agreement rate will be reported, and any disagreements will be solved through discussion. A sensitivity analysis will be performed excluding studies judged to be at high risk of bias, or where risk of bias is unclear.

Patient and public involvement

A lived experience advisory group, led by MR and consisting of experts who have been involved in previous trials of MBCT, will be consulted for the interpretation of study results and their dissemination. A coproduced lay summary of results will be written for dissemination alongside papers for peer review journals.

IPD data collection and aggregation

All corresponding authors of the articles selected for inclusion were contacted via email by TB with an invitation to participate in the review and to share the IPD from their primary study. The standard letter described all relevant details of the study including its purpose, main research questions, and the variables of interest and ended with a request to share the raw data from the study. Corresponding authors of studies were invited to serve as collaborators for the project. If corresponding authors did not respond within four weeks, we sent a second email request. If this had failed, a second author was contacted. Apart from one study, whose authors could not be contacted, none of the selected studies required contact attempts beyond a second author and the responding authors agreed to share their data.

Data checking and integrity

Authors were requested to provide a codebook or data dictionary explaining variable names, coding of values, measurement units etc. After accepting the invitation to collaborate and signing the data transfer agreement, the study authors shared their IPD via secure data transfer portal at the University of Surrey. Data is stored securely on University of Surrey servers in accordance with the European Union's General Data Protection Regulation Act and the UK's Data Protection Act 2018.

For peer review only - http://bmjopen.bmj.com/site/about/guidelines.xhtml

Received datasets will be reviewed to assess completeness and checked against the reported data from relevant study publications. The numbers of participants, overall and by arm, will be confirmed to correspond with the numbers in the study publication. Ranges of values will be inspected for clearly erroneous data. Baseline participant demographic data will be inspected by arm, with the aim of checking that the IPD matches the data reported in the publication, in terms of the numbers of participants with reported data, and overall aggregated values for each variable. Discrepancies will be referred to the study authors for investigation and any remaining discrepancies will be recorded.

Although the main search and initial receipt of IPD occurred before the submission of this protocol for publication, the protocol was registered in PROSPERO (CRD42022332039) beforehand. No data analysis or outcome-driven decisions were made prior to finalizing the protocol, and the methods described here were pre-specified prior to data inspection.

Data harmonization

Once datasets from the individual studies have been checked and outcomes standardised, they will be merged into the final dataset. The final dataset for analysis will be checked again for accuracy by a researcher in the study team.

STATISTICAL ANALYSIS

To finalise the selection of moderator variables, we will determine presence of these variables across the individual data sets and decide on ways to standardise these variables where needed, for example by collapsing categories. Potential moderator variables will be included in the analyses if the variable has at least 40% available data in the final dataset and has been recorded by at least 4 studies.

To investigate the efficacy of MBCT compared to control conditions and test moderating effects of individual patient level and study level characteristics, we will conduct one-stage and two-stage IPD meta-analyses (IPD-MAs). Random-effects models will be used

For peer review only - http://bmjopen.bmj.com/site/about/guidelines.xhtml

throughout because we expect high statistical heterogeneity due to variation in control groups (treatment as usual, active controls) and study populations (treatment non-responders/remitters, treatment-resistance, chronic depression).

The main research question of the study (whether MBCT is efficacious compared to control conditions) will be addressed via a maximum of three pairwise comparisons: (1) MBCT versus treatment-as-usual controls, (2) MBCT versus active controls, and (3) MBCT versus all controls.

By default, all analyses will be conducted in a Bayesian framework using JAGS (Just Another Gibbs Sampler)/BUGS (Bayesian analysis Using Gibbs Sampling) [56]. We chose a Bayesian approach because it offers greater flexibility in the parametrization of the (one-stage) IPD-MA models, and because this allows for a more intuitive interpretation of results based on posterior tail probabilities [57] . Across all analyses, findings will be considered "significant" when the credibility interval of the relevant effect does not include zero.

Two-stage random effects meta-analysis

We will first conduct a conventional meta-analysis using data extracted from the published papers. We will calculate Hedges' g to determine the effect sizes of the difference between the intervention and the control conditions reported in the papers. To investigate heterogeneity of effect sizes, we will calculate I^2, the percentage of variation not attributable to sampling error, as well as 95% prediction intervals (PIs) around the pooled effect [58, 59] . Leave-one-out analyses will be conducted to identify potential outliers and/or influential studies. Small-study effects will be investigated visually using funnel plot inspection and the Egger's test for asymmetry. A weakly informative Half-Cauchy (0,0.5) prior will be used for the between-study heterogeneity variance τ^2 [60], and flat normal priors for all other parameters. A detailed justification for this prior setup has been derived in previous work [61].

For peer review only - http://bmjopen.bmj.com/site/about/guidelines.xhtml

One-stage random effects meta-analysis using IPD

One-stage IPD meta-analysis models will be used as the primary analytic approach. To analyse the effects on common metrics-converted symptom severity scores, an identity-link linear mixed effects model with stratified intercepts and trial-specific (heteroscedastic) error terms will be used. Let y_{ik} be the depressive symptom severity of patient *i* in trial *k* (with *k*=1, …, *K* studies included in the meta-analysis). The one-stage IPD can then be defined as:

$$y_{ik} = \alpha_k + \theta T_{ik} + u_k T_{ik} + \beta_k (X_{ik} - \bar{X}_{ik}) + \varepsilon_{ik} \quad (1)$$

$$u_k \sim \mathcal{N}(0, \tau_\theta^2) \qquad \varepsilon_{ik} \sim \mathcal{N}(0, \sigma_k^2)$$

Where T_{ik} is the treatment indicator, X_{ik} is the symptom severity score at baseline, $\bar{X}_{ik}$ is the mean of X for each specific trial, α_k is the stratified trial intercept, and u_k is the trial-specific random slope of the average treatment effect θ. For this model, priors mirroring the two-stage approach will be used throughout, with a uniform distribution assigned to σ_k^2.

The same model specification, but with a logit link, will be used to model the effects on binary outcomes, including remission, clinically meaningful improvement and clinically meaningful deterioration based on PHQ-9 thresholds:

$$\log_e\left(\frac{\pi_{ik}}{1 - \pi_{ik}}\right) = \alpha_k + \theta T_{ik} + u_k T_{ik} + \beta_k (X_{ik} - \bar{X}_{ik}) + \varepsilon_{ik} \quad (2)$$

$$y_{ik} \sim \text{Bern}(\pi_{ik}) \qquad u_k \sim \mathcal{N}(0, \tau_\theta^2) \qquad \varepsilon_{ik} \sim \mathcal{N}(0, \sigma_k^2)$$

Where y_{ik} now represents the patient's binary outcome of interest. Numbers needed to treat (*NNT*) corresponding to the estimated SMD values will be calculated using the method by Furukawa and Leucht [62], and as the inverse of the absolute risk reduction otherwise.

For peer review only - http://bmjopen.bmj.com/site/about/guidelines.xhtml

Moderator analyses

We will explore potential moderators of treatment effect by adding individual participant level and study level treatment-covariate interactions into the main IPD-MA model. To optimise power, these analyses will be done on treatment effects at post-treatment as all studies can provide data for this timepoint.

Missing data

We will record the percentage of individual participant missing data for baseline characteristics and outcomes in each of the studies included in the meta-analyses. All analyses will be conducted according to the "intention-to-treat" principle, so that analyses target a treatment policy estimand [63]. Missing data will be handled using multiple imputation (fully conditional specification; MICE algorithm [64] under the missing at random (MAR) assumption. For the main effectiveness analysis, multilevel two-stage imputation models with heteroscedastic errors will be used to account for the nested data structure [65]. Highly collinear variables will be removed as predictors, as well as variables with systematically missing information ("structural zeros"). A total of m=50 imputation sets will be generated. A separate imputation model will be constructed for each focal moderator, including auxiliary variables with a maximum of 10% missingness. Imputations will be generated separately for each treatment groups (intervention, control) to account for treatment-covariate interactions. All analysis models will be fitted in the multiply imputed data. Parameter estimates and test statistics will then be aggregated by mixing the draws from the posterior distribution of each completed dataset [66]. Sensitivity analyses will be performed to compare results using observed and imputed data [67] .

Subgroup analyses

We plan to examine the efficacy of MBCT and potential moderators of treatment outcomes in subgroups defined by the conventional descriptive categories used by the

For peer review only - http://bmjopen.bmj.com/site/about/guidelines.xhtml

individual studies to define their populations, distinguishing between studies that recruited patients characterised as treatment non-responders/remitters, treatment-resistant, or as suffering from chronic depression. These analyses will be based on subsamples created from the final IPD dataset.

Sensitivity analyses

As a sensitivity analysis, we will repeat all main effectiveness analyses using equivalent frequentist (one- and two-stage) approaches. Additionally, we will conduct sensitivity analyses to investigate whether sources of heterogeneity affect the overall effect size estimate and robustness of our findings. This will include exploring the role of risk of bias and other potential study level sources of heterogeneity that may become obvious after data have been examined. These analyses will be conducted using a two-stage IPD-MA model.

DISCUSSION

This IPD meta-analysis will provide estimates of efficacy for the use of MBCT in DTD and offer comprehensive information about potential moderators of treatment effects. As such it will contribute to arguments in favour of or against implementing MBCT as a treatment for DTD and may help to decide whether MBCT is a recommendable treatment option for this group. This is important given the need to improve the care and management of DTD and the increasing recognition that psychological therapies can have an integral role in this endeavour. Information about potential moderators of treatment effects will help to refine the understanding of who may or may not benefit from MBCT for DTD

The chosen approach using IPD has several advantages over conventional meta-analyses including the possibility to assess whether individual participant level characteristics influence treatment effects with more specificity and power. Limitations of the approach include the fact that inconsistencies across studies regarding the format and type of variables

For peer review only - http://bmjopen.bmj.com/site/about/guidelines.xhtml

reported may restrict our ability to investigate moderators of treatment effects and that transformation of outcome measures to a common metric across all studies may come with a small loss of information. To address this latter point, the current study will complement one-stage IPD analyses with a conventional two-stage approach. A more general concern that is often raised for IPD meta-analyses is inclusion bias as it may be difficult to obtain IPD for all selected studies.

To our knowledge, this is the first IPD meta-analysis to investigate the effects of MBCT in DTD and assess moderators of treatment effects. As evidence from trials of MBCT for DTD has now accumulated to a point where the use of MBCT for DTD can be considered for endorsement in treatment guidelines, findings from this IPD meta-analysis have the potential to inform treatment recommendations and decisions regarding implementation. The results will provide clinicians, healthcare providers and people who are suffering from DTD with information on the characteristics that may make it more or less likely for patients to benefit from this treatment and thus facilitate implementation.

ETHICS AND DISSEMINATION

No local ethical review was necessary following consultation with the Ethics and Governance Board of the University of Surrey. The investigators of the primary trials have local ethical approval for sharing the data. TB and MH will be responsible for oversight of the database and analyses will be conducted by MH and TB. Patient privacy will be ensured by adhering to University of Surrey guidance on data management and storage. Exchange of data is governed by inter-institutional data sharing agreements. All data were pseudonymised (de-identified) before sharing, shared through secure upload facilities and are stored on secure servers. The data set will not be open access. Researchers of good standing may receive access pending on institutional approvals on data transfer.

For peer review only - http://bmjopen.bmj.com/site/about/guidelines.xhtml

We will disseminate the work in peer-reviewed journals, provide lay summaries and discuss findings with patient and lived experience groups. Other means of communication to disseminate the results will include conference presentations and social media announcements.

CONTRIBUTIONS

TB, MN, JM, MV and MH conceptualised and designed the study. TB developed the search strategy and contacted the primary authors, who contributed to further refinement of the design and approach of the study. TB, MV and MH are responsible for building the database. MH provides statistical expertise and is responsible for data analyses. TB drafted the protocol manuscript with statistical input by MH, which was critically revised by all authors. All authors read and approved the final version. TB serves as the guarantor of the review.

FUNDING STATEMENT

This research received no specific grant from any funding agency in the public, commercial or not-for-profit sectors.

ACKNOWLEDGMENTS

JMM has a ‘Miguel Servet’ research contract from the ISCIII (CP21/00080), and he is grateful to the Spanish CIBER of Epidemiology and Public Health (CBERESP CB22/02/00052< ISCIII) for their support.

COMPETING INTERESTS STATEMENT

TB is the co-author of a book on mindfulness-based interventions and regularly offers workshops on mindfulness-based interventions. He is the co-investigator of a programme grant evaluating an adapted MBCT course for adolescents experiencing depression.

MN is author of a book chapter on mindfulness-based interventions and offers workshops on mindfulness-based interventions.

For peer review only

For peer review only - http://bmjopen.bmj.com/site/about/guidelines.xhtml

JM is the director of the Achtsamkeitsinstitut Ruhr (an institute offering mindfulness training) and principal investigator of several DFG (German Science Foundation) research projects. He receives royalties from mindfulness books he has authored.

MV reports no conflicts of interest.

JMGW co-developed MBCT, and was founding Director of the University of Oxford Mindfulness Centre. He receives royalties from books describing the MBCT program. He also receives honoraria from lectures and training workshops on mindfulness.

AC leads a professional practice (Istituto Mente Corpo) that offers courses of mindfulness-based interventions.

SE is the author of book on Mindfulness-Based Cognitive Therapy for the treatment of chronic and treatment-resistant depression.

KD declares no conflicts of interest.

ZS received book royalties from Guilford Press, workshop fees from the Centre for Mindfulness studies, and revenue from online sales at Mindful Noggin Inc., which are all related to his work as a co-founder of Mindfulness-Based Cognitive Therapy (MBCT).

MCM declares no conflicts of interest.

AS is the founder and director of the Radboudumc Expertise Centre for Mindfulness and co-investigator of a number of studies examining the effectiveness and implementation of mindfulness-based interventions for patients with psychological or somatic disorders and health care professionals.

AAF declares no conflicts of interest.

MGT declares no conflicts of interest.

JMM is associated with the University of Oxford Mindfulness Research Centre.

BDD is the lead of the University of Exeter AccEPT clinic, which offers courses of MBCT.

For peer review only - http://bmjopen.bmj.com/site/about/guidelines.xhtml

CS is a member of a training organisation that has been commissioned by NHS England to deliver MBCT training across NHS Talking Therapies services in England and co-leads the Sussex Mindfulness Centre. CS is a co-investigator of a programme grant evaluating an adapted MBCT course for adolescents experiencing depression and co-investigator on a grant evaluating an adapted MBCT course for NHS staff.

FAR regularly provides workshops on mindfulness-based interventions.

MR declares no conflicts of interest.

MH serves as a statistical consultant for HelloBetter/GetOn Institut für Gesundheitstrainings GmbH, a company that implements digital mental health therapeutics in routine care.

REFERENCES

1. Collaborators G 2019 MD. Global, regional, and national burden of 12 mental disorders in 204 countries and territories, 1990–2019: a systematic analysis for the Global Burden of Disease Study 2019. Lancet Psychiatry. 2022;9:137–50.

2. McIntyre RS, Alsuwaidan M, Baune BT, Berk M, Demyttenaere K, Goldberg JF, et al. Treatment-resistant depression: definition, prevalence, detection, management, and investigational interventions. World Psychiatry. 2023;22:394–412.

3. Rush AJ, Sackeim HA, Conway CR, Bunker MT, Hollon SD, Demyttenaere K, et al. Clinical research challenges posed by difficult-to-treat depression. Psychol Med. 2022;52:419–32.

4. McAllister-Williams RH, Arango C, Blier P, Demyttenaere K, Falkai P, Gorwood P, et al. The identification, assessment and management of difficult-to-treat depression: An international consensus statement. J Affect Disord. 2020;267:264–82.

5. Jaffe DH, Rive B, Denee TR. The humanistic and economic burden of treatment-resistant depression in Europe: a cross-sectional study. BMC Psychiatry. 2019;19:247.

6. Bhattacharya R, Shen C, Sambamoorthi U. Excess risk of chronic physical conditions associated with depression and anxiety. BMC Psychiatry. 2014;14.

7. Markowitz JC, Wright JH, Peeters F, Thase ME, Kocsis JH, Sudak DM. The Neglected Role of Psychotherapy for Treatment-Resistant Depression. Am J Psychiatry. 2022;179:90–3.

For peer review only - http://bmjopen.bmj.com/site/about/guidelines.xhtml

8. McHugh RK, Whitton SW, Peckham AD, Welge JA, Otto MW. Patient Preference for Psychological vs Pharmacologic Treatment of Psychiatric Disorders: A Meta-Analytic Review. J Clin Psychiatry. 2013;74:595–602.

9. Segal ZV, Williams JMG, Teasdale J. Mindfulness-Based Cognitive Therapy for Depression. 2013.

10. Kuyken W, Warren FC, Taylor RS, Whalley B, Crane C, Bondolfi G, et al. Efficacy of Mindfulness-Based Cognitive Therapy in Prevention of Depressive Relapse: An Individual Patient Data Meta-analysis From Randomized Trials. Jama Psychiat. 2016;73:565.

11. Ma SH, Teasdale JD. Mindfulness-Based Cognitive Therapy for Depression: Replication and Exploration of Differential Relapse Prevention Effects. J Consult Clin Psych. 2004;72:31–40.

12. Teasdale JD, Segal ZV, Williams JMG, Ridgeway VA, Soulsby JM, Lau MA. Prevention of relapse/recurrence in major depression by mindfulness-based cognitive therapy. J Consult Clin Psych. 2000;68:615–23.

13. Creswell JD, Lindsay EK. How Does Mindfulness Training Affect Health? A Mindfulness Stress Buffering Account. Current Directions in Psychological Science. 2014;23.

14. Lindsay EK, Creswell DJ. Mechanisms of mindfulness training: Monitor and Acceptance Theory (MAT). Clinical Psychology Review. 2017;51.

15. Eisendrath SJ, Delucchi K, Bitner R, Fenimore P, Smit M, McLane M. Mindfulness-Based Cognitive Therapy for Treatment-Resistant Depression: A Pilot Study. Psychother Psychosom. 2008;77:319–20.

16. Barnhofer T, Crane C, Hargus E, Amarasinghe M, Winder R, Williams JMG. Mindfulness-based cognitive therapy as a treatment for chronic depression: A preliminary study. Behav Res Ther. 2009;47:366–73.

17. Eisendrath SJ, Gillung E, Delucchi KL, Segal ZV, Nelson JC, McInnes LA, et al. A Randomized Controlled Trial of Mindfulness-Based Cognitive Therapy for Treatment-Resistant Depression. Psychother Psychosom. 2016;85:99–110.

18. Cladder-Micus MB, Speckens AEM, Vrijsen JN, Donders ART, Becker ES, Spijker J. Mindfulness-based cognitive therapy for patients with chronic, treatment-resistant depression: A pragmatic randomized controlled trial. Depress Anxiety. 2018;35:914–24.

19. Michalak J, Schultze M, Heidenreich T, Schramm E. A randomized controlled trial on the efficacy of mindfulness-based cognitive therapy and a group version of cognitive behavioral analysis system of psychotherapy for chronically depressed patients. J Consult Clin Psych. 2015;83:951–63.

20. Chiesa A, Castagner V, Andrisano C, Serretti A, Mandelli L, Porcelli S, et al. Mindfulness-based cognitive therapy vs. psycho-education for patients with major depression

For peer review only

For peer review only - http://bmjopen.bmj.com/site/about/guidelines.xhtml

who did not achieve remission following antidepressant treatment. Psychiat Res. 2015;226:474–83.

21. Garcia A, Yáñez AM, Bennasar-Veny M, Navarro C, Salva J, Ibarra O, et al. Efficacy of an adjuvant non-face-to-face multimodal lifestyle modification program for patients with treatment-resistant major depression: A randomized controlled trial. Psychiatry Res. 2023;319:114975.

22. Foroughi A, Sadeghi K, Parvizifard A, Moghadam AP, Davarinejad O, Farnia V, et al. The effectiveness of mindfulness-based cognitive therapy for reducing rumination and improving mindfulness and self-compassion in patients with treatment-resistant depression. Trends Psychiatry Psychotherapy. 2020;42:138–46.

23. Mathew T, Nordström K. Comparison of One-Step and Two-Step Meta-Analysis Models Using Individual Patient Data. Biom J. 2010;52:271–87.

24. Clarke MJ. Individual patient data meta-analyses. Best Pr Res Clin Obstet Gynaecol. 2005;19:47–55.

25. Riley RD, Lambert PC, Abo-Zaid G. Meta-analysis of individual participant data: rationale, conduct, and reporting. BMJ. 2010;340 feb05 1:c221.

26. Jones AP, Riley RD, Williamson PR, Whitehead A. Meta-analysis of individual patient data versus aggregate data from longitudinal clinical trials. Clin Trials. 2009;6:16–27.

27. Cooper H, Patall EA. The Relative Benefits of Meta-Analysis Conducted With Individual Participant Data Versus Aggregated Data. Psychol Methods. 2009;14:165–76.

28. Stewart LA, Clarke M, Rovers M, Riley RD, Simmonds M, Stewart G, et al. Preferred Reporting Items for a Systematic Review and Meta-analysis of Individual Participant Data: The PRISMA-IPD Statement. Jama. 2015;313:1657–65.

29. Löfholm CA, Brännström L, Olsson M, Hansson K. Treatment-as-usual in effectiveness studies: What is it and does it matter? Int J Soc Welf. 2013;22:25–34.

30. Kabat-Zinn J. Mindfulness-Based Interventions in Context: Past, Present, and Future. Clinical Psychology: Science and Practice. 2003;10:144–56.

31. Association AP. Diagnostic and statistical manual of mental disorders (5th ed.). Arlington, VA: Author; 2013.

32. Association AP. Diagnostic and statistical manual of mental disorders (4th ed.). Washington, DC: Author; 2000.

33. Korten NCM, Comijs HC, Lamers F, Penninx BWJH. Early and late onset depression in young and middle aged adults: Differential symptomatology, characteristics and risk factors? J Affect Disord. 2012;138:259–67.

For peer review only

For peer review only - http://bmjopen.bmj.com/site/about/guidelines.xhtml

34. Devanand DP, Adorno E, Cheng J, Burt T, Pelton GH, Roose SP, et al. Late onset dysthymic disorder and major depression differ from early onset dysthymic disorder and major depression in elderly outpatients. J Affect Disord. 2004;78:259–67.

35. Wahl I, Löwe B, Bjorner JB, Fischer F, Langs G, Voderholzer U, et al. Standardization of depression measurement: a common metric was developed for 11 self-report depression measures. J Clin Epidemiology. 2014;67:73–86.

36. Rush AJ, Trivedi MH, Ibrahim HM, Carmody TJ, Arnow B, Klein DN, et al. The 16-Item quick inventory of depressive symptomatology (QIDS), clinician rating (QIDS-C), and self-report (QIDS-SR): a psychometric evaluation in patients with chronic major depression. Biol Psychiatry. 2003;54:573–83.

37. Kroenke K, Spitzer RL, Williams JBW. The PHQ-9: Validity of a brief depression severity measure. J Gen Intern Med. 2001;16:606–13.

38. Löwe B, Unützer J, Callahan CM, Perkins AJ, Kroenke K. Monitoring Depression Treatment Outcomes With the Patient Health Questionnaire-9. Med Care. 2004;42:1194–201.

39. Hudgens S, Floden L, Blackowicz M, Jamieson C, Popova V, Fedgchin M, et al. Meaningful Change in Depression Symptoms Assessed with the Patient Health Questionnaire (PHQ-9) and Montgomery-Åsberg Depression Rating Scale (MADRS) Among Patients with Treatment Resistant Depression in Two, Randomized, Double-blind, Active-controlled Trials of Esketamine Nasal Spray Combined With a New Oral Antidepressant. J Affect Disord. 2021;281:767–75.

40. Turkoz I, Alphs L, Singh J, Jamieson C, Daly E, Shawi M, et al. Clinically meaningful changes on depressive symptom measures and patient-reported outcomes in patients with treatment-resistant depression. Acta Psychiatr Scand. 2021;143:253–63.

41. Hölzel L, Härter M, Reese C, Kriston L. Risk factors for chronic depression — A systematic review. J Affect Disorders. 2011;129:1–13.

42. Seedat S, Scott KM, Angermeyer MC, Berglund P, Bromet EJ, Brugha TS, et al. Cross-National Associations Between Gender and Mental Disorders in the World Health Organization World Mental Health Surveys. Arch Gen Psychiatry. 2009;66:785–95.

43. Buckman JEJ, Saunders R, Stott J, Arundell L-L, O'Driscoll C, Davies MR, et al. Role of age, gender and marital status in prognosis for adults with depression: An individual patient data meta-analysis. Epidemiology Psychiatr Sci. 2021;30:e42.

44. Trivedi MH, Rush AJ, Wisniewski SR, Nierenberg AA, Warden D, Ritz L, et al. Evaluation of Outcomes With Citalopram for Depression Using Measurement-Based Care in STARD: Implications for Clinical Practice. Am J Psychiatry. 2006;163:28–40.

45. Oosten AJ van, Mens K van, Blonk RWB, Burdorf A, Tiemens B. The relationship between having a job and the outcome of brief therapy in patients with common mental disorders. BMC Psychiatry. 2023;23:910.

For peer review only

For peer review only - http://bmjopen.bmj.com/site/about/guidelines.xhtml

46. Nanni V, Uher R, Danese A. Childhood Maltreatment Predicts Unfavorable Course of Illness and Treatment Outcome in Depression: A Meta-Analysis. Am J Psychiat. 2012;169:141–51.

47. Klein DN, Schatzberg AF, McCullough JP, Dowling F, Goodman D, Howland RH, et al. Age of onset in chronic major depression: relation to demographic and clinical variables, family history, and treatment response. J Affect Disord. 1999;55:149–57.

48. Bromberger JT, Schott L, Kravitz HM, Joffe H. Risk factors for major depression during midlife among a community sample of women with and without prior major depression: are they the same or different? Psychol Med. 2015;45:1653–64.

49. Myklebost SB, Gjestad R, Inal Y, Hammar Å, Nordgreen T. Predictors of Treatment Response to an Internet-Delivered Intervention Targeting Residual Cognitive Symptoms After Major Depressive Disorder. Front Psychiatry. 2022;13:795698.

50. Courtney DB, Watson P, Krause KR, Chan BWC, Bennett K, Gunlicks-Stoessel M, et al. Predictors, Moderators, and Mediators Associated With Treatment Outcome in Randomized Clinical Trials Among Adolescents With Depression. JAMA Netw Open. 2022;5:e2146331.

51. Cuijpers P, Sijbrandij M, Koole SL, Andersson G, Beekman AT, Reynolds CF. Adding psychotherapy to antidepressant medication in depression and anxiety disorders: a meta-analysis. World Psychiatry. 2014;13:56–67.

52. DeRubeis RJ, Hollon SD, Amsterdam JD, Shelton RC, Young PR, Salomon RM, et al. Cognitive Therapy vs Medications in the Treatment of Moderate to Severe Depression. Arch Gen Psychiatry. 2005;62:409–16.

53. Button KS, Wiles NJ, Lewis G, Peters TJ, Kessler D. Factors associated with differential response to online cognitive behavioural therapy. Soc Psychiatry Psychiatr Epidemiology. 2012;47:827–33.

54. Furlan AD, Pennick V, Bombardier C, Tulder M van, Group EB Cochrane Back Review. 2009 Updated Method Guidelines for Systematic Reviews in the Cochrane Back Review Group. Spine. 2009;34:1929–41.

55. Sterne JAC, Savović J, Page MJ, Elbers RG, Blencowe NS, Boutron I, et al. RoB 2: a revised tool for assessing risk of bias in randomised trials. Bmj. 2019;366:l4898.

56. Lunn D, Jackson C, Best N, Thomas A, Spiegelhalter D. The BUGS Book, A Practical Introduction to Bayesian Analysis. 2019. https://doi.org/10.1201/b13613.

57. Goligher EC, Heath A, Harhay MO. Bayesian statistics for clinical research. Lancet. 2024;404:1067–76.

58. Higgins JPT, Thompson SG, Deeks JJ, Altman DG. Measuring inconsistency in meta-analyses. BMJ. 2003;327:557–60.

59. Higgins JPT, Thompson SG. Quantifying heterogeneity in a meta-analysis. Stat Med. 2002;21:1539–58.

For peer review only - http://bmjopen.bmj.com/site/about/guidelines.xhtml

60. Harrer M, Cuijpers P, Furukawa TA, Ebert DD. Doing Meta-Analysis with R, A Hands-On Guide. 2022. https://doi.org/10.1201/9781003107347.

61. Harrer M. Heterogeneity Prior Justification. 2022. https://osf.io/3tfm6. Accessed 16 May 2025.

62. Furukawa TA, Leucht S. How to Obtain NNT from Cohen's d: Comparison of Two Methods. PLoS ONE. 2011;6:e19070.

63. Han S, Zhou X. Defining estimands in clinical trials: A unified procedure. Stat Med. 2023;42:1869–87.

64. Buuren van, Groothuis-Oudshoorn K. mice: multivariate imputation by chained equations in R. Journal of Statistical Software. 2011;45:1–67.

65. Resche-Rigon M, White IR. Multiple imputation by chained equations for systematically and sporadically missing multilevel data. Stat Methods Méd Res. 2018;27:1634–49.

66. Zhou X, Reiter JP. A Note on Bayesian Inference After Multiple Imputation. Am Stat. 2010;64:159–63.

67. Burgess S, White IR, Resche-Rigon M, Wood AM. Combining multiple imputation and meta-analysis with individual participant data. Stat Med. 2013;32:4499–514.

For peer review only

APPENDIX

Search Terms

Database	Search Term
Web of Science	(“MDE” OR “major depressive episode” OR “MDD” OR “major depressive disorder” OR “major depression” OR “depression” OR “dysthymia” OR “dysthymic”) AND (“chronic” OR “persistent” OR “treatment-resistant” OR “non-responders” OR “non-response” OR “non-remitted” OR “dysthymia” OR “dysthymic”) AND (“MBCT” OR "mindfulness-based cognitive therapy") AND (“randomized controlled trial” OR “randomised controlled trial” OR “randomized-controlled trial” OR “randomised-controlled trial” OR “RCT” OR “randomized” or “randomised” OR “randomly allocated” OR “randomly assigned”)
Scopus	(“MDE” OR “major depressive episode” OR “MDD” OR “major depressive disorder” OR “major depression” OR “depression” OR “dysthymia” OR “dysthymic”) AND (“chronic” OR “persistent” OR “treatment-resistant” OR “non-responders” OR “non-response” OR “non-remitted” OR “dysthymia” OR “dysthymic”) AND (“MBCT” OR "mindfulness-based cognitive therapy") AND (“randomized controlled trial” OR “randomised controlled trial” OR “randomized-controlled trial” OR “randomised-controlled trial” OR “RCT” OR “randomized” or “randomised” OR “randomly allocated” OR “randomly assigned”)
PubMed	(“MDE” OR “major depressive episode” OR “MDD” OR “major depressive disorder” OR “major depression” OR “depression” OR “dysthymia” OR “dysthymic”) AND (“chronic” OR “persistent” OR “treatment-resistant” OR “non-responders” OR “non-response” OR “non-remitted” OR “dysthymia” OR “dysthymic”) AND (“MBCT” OR "mindfulness-based cognitive therapy") AND (“randomized controlled trial” OR “randomised controlled trial” OR “randomized-controlled trial” OR “randomised-controlled trial” OR “RCT” OR “randomized” or “randomised” OR “randomly allocated” OR “randomly assigned”)
PsycInfo	(“MDE” OR “major depressive episode” OR “MDD” OR “major depressive disorder” OR “major depression” OR “depression” OR “dysthymia” OR “dysthymic”) **AND** (“chronic” OR “persistent” OR “treatment-resistant” OR “non-responders” OR “non-response” OR “non-remitted” OR “dysthymia” OR “dysthymic”) **AND** (“MBCT” OR "mindfulness-based cognitive therapy") **AND** (“randomized controlled trial” OR “randomised controlled trial” OR “randomized-controlled trial” OR “randomised-controlled trial” OR “RCT” OR “randomized” or “randomised” OR “randomly allocated” OR “randomly assigned”)
Embase	(‘MDE’ OR ‘major depressive episode’ OR ‘MDD’ OR ‘major depressive disorder’ OR ‘major depression’ OR ‘depression’ OR ‘dysthymia’ OR ‘dysthymic’) **AND** (‘chronic’ OR ‘persistent’ OR ‘treatment-resistant’ OR ‘non-responders’ OR ‘non-response’ OR ‘non-remitted’ OR ‘dysthymia’ OR ‘dysthymic’) **AND** (‘MBCT’ OR ‘mindfulness-based cognitive therapy’) **AND** (‘randomized controlled trial’ OR ‘randomised controlled trial’ OR ‘randomized-controlled trial’ OR ‘randomised-controlled trial’ OR ‘RCT’ OR ‘randomized’ or ‘randomised’ OR ‘randomly allocated’ OR ‘randomly assigned’)

For peer review only - http://bmjopen.bmj.com/site/about/guidelines.xhtml

Cochrane Controlled Trials	(“MDE” OR “major depressive episode” OR “MDD” OR “major depressive disorder” OR “major depression” OR “depression” OR “dysthymia” OR “dysthymic”) AND (“chronic” OR “persistent” OR “treatment-resistant” OR “non-responders” OR “non-response” OR “non-remitted” OR “dysthymia” OR “dysthymic”) AND (“MBCT” OR "mindfulness-based cognitive therapy") AND (“randomized controlled trial” OR “randomised controlled trial” OR “randomized-controlled trial” OR “randomised-controlled trial” OR “RCT” OR “randomized” or “randomised” OR “randomly allocated” OR “randomly assigned”)

For peer review only

BMJ Open

Efficacy and Moderators of Mindfulness-Based Cognitive Therapy (MBCT) in 'Difficult to Treat' Depression: Protocol for a Systematic Review and Individual Patient Data Meta-Analysis of Randomised Controlled Trials

Journal:	*BMJ Open*
Manuscript ID	bmjopen-2025-106350.R1
Article Type:	Protocol
Date Submitted by the Author:	12-Sep-2025
Complete List of Authors:	Barnhofer, Thorsten; University of Surrey,<br>Niemi, Maria; Karolinska Institutet, Department of Global Public Health<br>Michalak, Johannes; Witten/Herdecke University, Department of Psychology and Psychotherapy<br>Velana, Maria; Witten/Herdecke University, Department of Psychology and Psychotherapy<br>Williams, J Mark; University of Oxford, Department of Psychiatry<br>Chiesa, Alberto; Università di Bologna<br>Eisendrath, Stuart; University of California at San Francisco<br>Delucchi, Kevin; University of California at San Francisco<br>Segal, Zindel; University of Toronto - Scarborough, Graduate Department of Psychological Clinical Science<br>Cladder-Micus, Mira; Radboud University Nijmegen<br>Speckens, Anne; Radboudumc Instituut voor Wetenschappelijk Onderwijs en Opleidingen, Psychiatry<br>Foroughi, Ali Akbar; Kermanshah University of Medical Sciences (KUMS), Department of Clinical Psychology<br>García-Toro, M; Research Network on Chronicity Primary Care and Health Promotion; University of the Balearic Islands; Health Research Institute of the Balearic Islands; University of the Balearic Islands, Department of Medicine<br>Montero-Marin, Jesus; Research & Innovation Unit Parc Sanitari Sant Joan de Déu; University of Oxford, Department of Psychiatry; Consortium for Biomedical Research in Epidemiology and Public Health<br>dunn, barney; University of Exeter<br>Strauss, Clara; Sussex Partnership NHS Foundation Trust; University of Sussex<br>Ruths, Florian; South London and Maudsley NHS Foundation Trust<br>Ryan, Mary; Royal College of Psychiatrists<br>Harrer, Mathias; Technical University of Munich, Department of Psychiatry and Psychotherapy; World Health Organization, Department of Clinical, Neuro and Developmental Psychology; Vrije Universiteit
**Primary Subject Heading**:	Mental health
Secondary Subject Heading:	Mental health

Keywords:	Meta-Analysis, Depression & mood disorders < PSYCHIATRY, Mindfulness

SCHOLARONE™
Manuscripts

Title

Efficacy and Moderators of Mindfulness-Based Cognitive Therapy (MBCT) in 'Difficult to Treat' Depression: Protocol for a Systematic Review and Individual Patient Data Meta-Analysis of Randomised Controlled Trials

Running Title

MBCT in 'Difficult to Treat' Depression

Authors and Affiliations

Thorsten Barnhofer [1], Maria Niemi [2, *], Johannes Michalak [3], Maria Velana [3], J. Mark G. Williams [4], Alberto Chiesa [5], Stuart Eisendrath [6], Kevin Delucchi [6], Zindel V. Segal [7], Mira Cladder-Micus [8], Anne Speckens [9], Ali Akbar Foroughi [10], Mauro Garcia-Toro [11, 12, 13, 14], Jesus Montero-Marin [15, 4, 16], Barnaby D. Dunn [17], Clara Strauss [18, 19], Florian A. Ruths [20], Mary Ryan [21], Mathias Harrer [22, 23, 24]

[1] School of Psychology, University of Surrey, Guildford, UK, t.barnhofer@surrey.ac.uk

[2] Department of Global Public Health, Karolinska Institutet, Stockholm, Sweden, maria.niemi@ki.se

[3] Department of Psychology and Psychotherapy, Witten/Herdecke University, Witten, Germany, Maria.Velana@uni-wh.de , Johannes.Michalak@uni-wh.de

[4] Department of Psychiatry, University of Oxford, Oxford, UK, mark.williams@psych.ox.ac.uk, jesus.monteromarin@psych.ox.ac.uk

[5] Institute of Psychiatry, Università di Bologna, Bologna, Italy, chiesa@scuola-spc.it

[6] Weill Institute for Neurosciences, University of California at San Francisco, San Francisco, USA, stuart.eisendrath@ucsf.edu, kevin.delucchi@ucsf.edu

[7] Graduate Department of Psychological Clinical Science, University of Toronto - Scarborough, Toronto, Canada, zindel.segal@utoronto.ca

For peer review only - http://bmjopen.bmj.com/site/about/guidelines.xhtml

[8] Behavioural Science Institute, Radboud University Nijmegen, Nijmegen, The Netherlands,

Mira.Cladder-Micus@radboudumc.nl

[9] Department of Psychiatry, Radboudumc, Nijmegen, The Netherlands,

anne.speckens@radboudumc.nl

[10] Department of Clinical Psychology, Kermanshah University of Medical Sciences (KUMS), Hermanshah, Iran, foroughi_2002@yahoo.com

[11] Research Network on Chronicity, Primary Care and Health Promotion (RICAPPS), Carlos III Health Institute, Madrid, Spain

[12] University Institute of Health Science Research (IUNICS), University of the Balearic Islands, Spain, mauro.garcia@uib.es

[13] Health Research Institute of the Balearic Islands (IdISBa), Palma, Spain

[14] Department of Medicine, University of the Balearic Islands, Palma, Spain

[15] Research & Innovation Unit, Parc Sanitari Sant Joan de Déu, Sant Boi de Llobregat, Barcelona, Spain

[16] Consortium for Biomedical Research in Epidemiology and Public Health (CIBER Epidemiology and Public Health-CIBERESP), Madrid, Spain

[17] Mood Disorders Centre, University of Exeter, Exeter, UK, b.d.dunn@exeter.ac.uk

[18] Sussex Partnership NHS Foundation Trust, Hove, UK, clara.strauss@nhs.net

[19] Sussex Mental Health Research Centre, University of Sussex, Brighton, UK

[20] South London and Maudsley NHS Foundation Trust, London, UK,

Florian.Ruths@slam.nhs.uk

[21] Royal College of Psychiatrists, London, UK, mary@highbag.co.uk

[22] Department of Psychiatry and Psychotherapy, School of Medicine and Health, Technical University of Munich, Munich, Germany

[23] Department of Clinical, Neuro and Developmental Psychology, World Health Organization

For peer review only

[24] Collaborating Center for Research and Dissemination of Psychological Interventions, Amsterdam, Public Health Research Institute, Vrije Universiteit, Amsterdam, The Netherlands, mathias.harrer@tum.de

* Corresponding author:

Associate Professor Maria Niemi, Department of Global Public Health, Karolinska Institutet, Stockholm, Sweden, maria.niemi@ki.se

For peer review only

Word Count

Abstract: 300

Main text: 4110

Keywords

Major Depression, Difficult-to-Treat, Mindfulness-Based Cognitive Therapy, Independent Patient Data, Meta-Analysis

For peer review only - http://bmjopen.bmj.com/site/about/guidelines.xhtml

ABSTRACT

Introduction: About 30% of depressed patients suffer from a protracted course in which the disorder continues to cause significant burden despite treatment efforts. While originally developed for relapse prevention, Mindfulness-Based Cognitive Therapy (MBCT) has increasingly been investigated in depressed patients with such ‘difficult-to-treat’ courses. This is a protocol for an individual participant data (IPD) meta-analysis aiming to determine efficacy and potential moderators of MBCT treatment effects in this group based on evidence from randomised controlled trials.

Methods and analysis: Systematic searches in PubMed, Web of Science, Scopus, PsycINFO, EMBASE, and the Cochrane Controlled Trials Register for randomised controlled trials were completed on 17 June 2024. Authors of identified studies have contributed IPD and data extractions have been completed. An update search will be conducted immediately before the start of data analyses. We will investigate the following outcomes: (a) self-reported and observer-reported severity of depression symptomatology, (b) remission, (c) clinically meaningful improvement and deterioration. One-stage and two-stage IPD meta-analyses will be conducted with one-stage models using the observed IPD from all studies simultaneously as the primary approach. One-stage IPD models will include stratified study intercepts and error terms as well as random effects to capture between-study heterogeneity. Moderator analyses will test treatment-covariate interactions for both individual patient level and study level characteristics.

Ethics and dissemination: The results will inform understanding of the use of MBCT in patients with current ‘difficult-to-treat’ depression and will contribute to arguments in favour of or against implementing MBCT as a treatment for this group. They will be published in a peer reviewed journal and made available to stakeholders in accessible formats. No local ethical review was necessary following consultation with the Ethics and Governance Board

For peer review only - http://bmjopen.bmj.com/site/about/guidelines.xhtml

of the University of Surrey. Guidance on patient data storage and management will be adhered to throughout.

PROSPERO registration number: CRD42022332039

For peer review only

For peer review only - http://bmjopen.bmj.com/site/about/guidelines.xhtml

STRENGTHS AND LIMITATIONS OF THIS STUDY

- The chosen approach using IPD has several advantages over conventional meta-analyses including the possibility to assess whether individual participant level characteristics influence treatment effects with more specificity and power
- Limitations of the approach include the fact that inconsistencies across studies regarding the format and type of variables reported may restrict our ability to investigate moderators of treatment effects
- Transformation of outcome measures to a common metric across all studies may come with a small loss of information.

For peer review only

INTRODUCTION

Major Depressive Disorder (MDD) is highly prevalent, debilitating [1] , and in about 30% of patients takes a recurrent or chronic course in which established treatments fail to bring sustained remission [2] . Recently framed under the broader heuristic of difficult-to-treat depression (DTD) [3, 4] , defined as "depression that continues to cause significant burden despite usual treatment efforts", such courses are associated with continuing functional impairment, reduced quality of life, and significantly increased risk for chronic physical and neurodegenerative disorders [5, 6] . Finding effective treatments for DTD remains an important challenge for depression research [7] .

While treatment approaches for DTD have traditionally been dominated by pharmacological strategies [8] , it is now increasingly acknowledged that effective management requires a broader emphasis on working with symptoms, maximising function and minimizing burden [4] . Psychological interventions have an important contribution to make in this context [9] and emerging evidence suggests that Mindfulness-Based Cognitive Therapy (MBCT [10]), an eight-week treatment that combines elements of cognitive therapy with mindfulness meditation, may be suited for this purpose [11] . Using a mental training approach, the intervention aims to provide patients with portable skills that enable them to disengage from habitual maladaptive responses and respond more adaptively to negative mood and stress.

MBCT was originally introduced for the prevention of relapse in patients who are currently in remission and has been proven to be effective for this purpose [12] , particularly in patients with highly recurrent courses (a use that is endorsed by treatment guidelines internationally). Early trials restricted the intervention to patients who were currently in full remission as it was assumed that intensive mindfulness practice may be too demanding for those who are suffering from current symptoms [13, 14] . However, as patients who are at

For peer review only - http://bmjopen.bmj.com/site/about/guidelines.xhtml

high risk of relapse are likely to show residual symptoms between episodes, this restriction was later loosened, and analyses across studies for relapse prevention show that treatment effects increase, rather than decrease, with level of residual symptoms [12] . From a theoretical perspective, this observation is in line with the view that mindfulness skills serve to buffer the effects of negative mood and stress [15, 16] , which implies that effects should become more visible under conditions of higher symptoms or stress.

Based on this reasoning, research has extended the use of MBCT to patients with current depression described as 'difficult-to-treat'. Preliminary studies investigating MBCT in treatment-resistant and chronic depression were published in 2008 [17] and 2009 [18] and several definitive randomised controlled trials have been conducted since then [19–24] , suggesting that evidence has accumulated to a point where the use of MBCT as a treatment for patients with current DTD can now be considered for guideline endorsement and wider implementation. It seems timely therefore to bring data from existing randomised controlled trials together and analyse outcome across studies.

For this purpose, we have pooled individual patient data (IPD) from these studies to conduct meta-analyses with one-stage random effects meta-analysis of independent patient data (IPD) as our primary approach given its advantages over conventional aggregate data meta-analyses [25–28] . The main aim of the analyses is to establish efficacy of MBCT for patients with DTD as compared to the control conditions utilised in the existing trials. Given the need for both effective symptom reduction and the maintenance of gains, we will aim to determine immediate effects at the end of the eight-week MBCT intervention (post-treatment) and effects in the longer-term after patients have finished the intervention and are free to continue engaging in mindfulness practice by themselves (follow-up).

An important advantage of IPD meta-analysis is that it allows to examine moderator or subgroup questions across the pooled sample and can thus serve to overcome limitations in

For peer review only - http://bmjopen.bmj.com/site/about/guidelines.xhtml

sample size and power for answering these questions in the original studies [29] . Information about potential moderators of treatment effects, such as severity of depression at entry to treatment or a history of bipolar disorder, is important as it can potentially help with implementation by providing more precise information about relative indications or contraindications of the intervention. A further aim of this IPD meta-analysis is therefore to explore effects of potential individual patient level (e.g. sociodemographic and clinical characteristics) and study level (e.g. delivery format) moderators of treatment effects in the pooled data set.

As the clinical concept of DTD does not link to an objective way of assessment, the current meta-analysis will include studies that have used a range of conventional definitions falling under the wider concept of DTD, including selecting participants based on treatment non-response/remission, treatment resistance or the presence of chronic depression. Subgroup analyses will allow us to determine the extent to which effects found in the aggregated data generalise across these conventional categories. Given that DTD reflects a wider construct encompassing a large and heterogeneous group of patients with different characteristics and different treatment histories, we will aim to carefully describe the sample both on the meta- and the study-level.

METHODS

General approach

This IPD meta-analysis is registered as part of a broader project on PROSPERO (registration number: CRD42022332039; date of registration in PROSPERO: 24 May 2022) and any key changes or amendments will be documented there. In addition to the direct comparisons between MBCT and control conditions, the wider project also includes studies of an individual psychotherapeutic intervention, Cognitive Behavioral Analysis System of Psychotherapy, and considers a wider range of outcomes, including both beneficial and adverse

For peer review only - http://bmjopen.bmj.com/site/about/guidelines.xhtml

treatment effects. The current protocol specifies procedures for the comparison between MBCT and the control conditions utilized in the existing MBCT trials only as we assume that this comparison is of particular interest given the need for establishing further treatment options for DTD. The moderator and sensitivity analyses described here were prespecified in this protocol prior to data analysis but were not detailed in the initial PROSPERO entry. They are included to address theoretically and clinically relevant questions, and all analyses (including null results) will be reported to minimise selective reporting. The protocol is registered retrospectively as searches to identify eligible papers were already conducted in 2024, with the last search having been performed on 17 June 2024 and an update search planned immediately before the start of data analysis. Corresponding authors of the selected studies were contacted and asked to provide raw data from their studies. The Preferred Reporting Items for Systematic Reviews and Meta-Analyses IPD statement [30] will be followed for the reporting of this study.

Eligibility criteria

Types of studies to be included

This IPD meta-analysis will include only peer-reviewed RCTs, published in English. There will be no restriction on the type of recruitment settings. The restriction to English-language publications is due to feasibility constraints and is recognised as a potential source of language bias.

Type of intervention

Included studies will have investigated the effects of manualised mindfulness-based cognitive therapy (MBCT) alone or as an adjunct treatment to treatment-as-usual (TAU) [31]. MBCT is a psychological group-based intervention that combines intensive training in mindfulness meditation with elements of cognitive behavioural therapy (CBT) for depression. Mindfulness can be operationally defined as non-judgemental awareness of present moment

For peer review only - http://bmjopen.bmj.com/site/about/guidelines.xhtml

experience [32]. As MBCT was originally designed to prevent relapse rather than to reduce symptoms during a current episode, we will accept studies that introduce minor adaptations to the delivery of the manualised approach (e.g. incorporating specific support for managing current depressive symptoms, particularly around the engagement in regular practice, and rephrasing psycho-educational components to address current depression rather than relapse, without altering the structure or core content of the manual). Other types of mindfulness-based interventions will not be included. MBCT studies will be considered regardless of delivery format (e.g. physical group setting versus videoconference).

Types of comparators

This may include TAU for depression, including pharmacological and psychological treatments (e.g., CBT) or active controls. The latter will include established interventions as well as control interventions designed to mimic effects of mindfulness-based interventions in some or all aspects with the exception of the core element of mindfulness.

Participants

We will include studies that recruited adults with current Major Depressive Disorder (MDD) [33, 34] and studies which included mixed groups of patients with residual depressive symptoms or full episodes in the context of a chronic or treatment-resistant course of the disorder. In the latter case, participants with only residual symptoms will be excluded from the primary analysis approach (one-stage meta-analysis). As DTD does not link to an objective way of assessment, we will opt for a maximally inclusive interpretation of the term and include any studies that selected patients based on previous treatment non-response/remission, treatment-resistance or chronicity. We will exclude studies with participants below the age of 18 and studies that have exclusively recruited participants over the age of 65 given that the factors involved in the onset, maintenance and recurrence of

For peer review only - http://bmjopen.bmj.com/site/about/guidelines.xhtml

depression in these age groups can differ from those in adults within this age bracket [35, 36] .

Outcomes

The main type of outcome will be severity of depressive symptomatology as assessed using standard depression outcome measures. Standard depression outcome measures may include self-report measures and observer-rated measures. If both types of measures are available, we will investigate effects on both types of outcome measures. If several measures are available for a single study, preference will be given to the measure that was used as the primary outcome of the original study. To compare outcomes across studies using different outcome measures, we will transform scores from these measures into standardised scores using the common metric developed by Wahl et al. [37] . Where a measure is not part of the common metric approach, we will transform the measure into one that is part of the common metric using published conversion tables [38] , and then derive the common metric.

To facilitate clinical interpretation of results, we will derive dichotomous outcomes reflecting remission, improvement and deterioration. To apply a common rule for all studies, common metric scores will be converted to the PHQ-9 and dichotomous outcomes computed based on this measure. Following conventions for the PHQ-9 used in pragmatic trials and health services, remission will be defined as a score of less than 10 [39, 40], i.e. a shift from "caseness" to a non-case score. Definition of clinically meaningful change will follow suggestions from recent trials in treatment-resistant depression, which has indicated a threshold of 6 or more points [41, 42] . We will therefore define clinically meaningful improvement as a reduction of 6 or more points and clinically meaningful deterioration as an increase of 6 or more points on the PHQ-9.

Secondary outcomes such as mindfulness, self-compassion and quality of life will be collected and may be used for future analysis.

For peer review only - http://bmjopen.bmj.com/site/about/guidelines.xhtml

Moderators

We will also investigate potential moderators of treatment response, i.e. factors that determine whether a person responds better to MBCT as compared to the control condition or vice versa. These will include potential moderators on the individual patient level (e.g. sociodemographic characteristics, history of childhood trauma, and clinical characteristics) and on the study level. We will include patient level characteristics in the analyses, if they are reported across a sufficient number of studies and datasets (see statistical analysis section for criteria).

Sociodemographic and clinical characteristics that we aim to investigate include age [43] , gender [44] , marital status [45] , education [46] , employment [47] , childhood adversity [48] , age at onset of depression [49] , number of previous episodes [50] , duration of current episode [51] , current suicidality [52] , current antidepressants [53] , past antidepressants and treatment-resistance to antidepressants [54] , co-morbid anxiety disorder, comorbid obsessive-compulsive disorder, co-morbid post-traumatic stress disorder, co-morbid substance abuse or dependence, co-morbid eating disorder, co-morbid personality disorder [43], and level of depressive symptoms at entry [55] . Study level characteristics will include type of delivery (remote versus face-to-face), type of control condition (TAU, active control), type of DTD (non-response/remission, treatment-resistance, chronic depression) and study quality.

Timing of outcome assessments

As follow-up periods of the studies differ, we will group follow-up periods in the following categories: 8 to 10 weeks post-randomisation (post-treatment), 11 to 34 weeks post-randomisation (follow-up 1), and 35 to 60 weeks post-randomisation (follow-up 2).

For peer review only - http://bmjopen.bmj.com/site/about/guidelines.xhtml

Searches and study selection

We searched PubMed, Web of Science, Scopus, PsychINFO, EMBASE, and the Cochrane Controlled Trials Register using index and free terms, jointly with Boolean operators, on four tiers, namely: (1) depressive disorder, (2) non-response, treatment resistance and chronicity, (3) mindfulness-based cognitive therapy, and (4) randomised-controlled trial. The search terms used for the different databases are shown in the appendix. We did not search grey literature or trial registries beyond the Cochrane Controlled Trials Register, and we did not consult experts outside of the research team.

We searched for any study published up until the day of the search, on 17 June 2024. There was no lower time limit. Initial screening of titles was performed by two independent reviewers (TB, JM), with the publication abstract being obtained if selected by at least one reviewer. Abstracts were screened by the same two independent reviewers, with disagreements about eligibility being referred to a third reviewer whose decision was final. An update search will be conducted immediately before the start of data analysis.

Risk of bias assessment

The validity of the studies will be assessed using the Risk of Bias tool by the Cochrane Collaboration (version 2) [56, 57] . Possible sources of bias assessed by the tool are (1) random sequence generation, (2) allocation concealment, (3) blinding of outcome assessors, (4) incomplete outcome data, (5) selective outcome reporting, and (6) other threats to validity including lack of compliance and different timing of outcome assessments. Data from the published papers of the trials will be evaluated by two independent assessors. Agreement rate will be reported, and any disagreements will be solved through discussion. A sensitivity analysis will be performed excluding studies judged to be at high risk of bias, or where risk of bias is unclear.

For peer review only

For peer review only - http://bmjopen.bmj.com/site/about/guidelines.xhtml

Patient and public involvement

A lived experience advisory group, led by MR and consisting of experts who have been involved in previous trials of MBCT, will be consulted for the interpretation of study results and their dissemination. A coproduced lay summary of results will be written for dissemination alongside papers for peer review journals.

IPD data collection and aggregation

All corresponding authors of the articles selected for inclusion were contacted via email by TB with an invitation to participate in the review and to share the IPD from their primary study. The standard letter described all relevant details of the study including its purpose, main research questions, and the variables of interest and ended with a request to share the raw data from the study. Corresponding authors of studies were invited to serve as collaborators for the project. If corresponding authors did not respond within four weeks, we sent a second email request. If this had failed, a second author was contacted. Apart from one study, whose authors could not be contacted, none of the selected studies required contact attempts beyond a second author and the responding authors agreed to share their data.

Data checking and integrity

Authors were requested to provide a codebook or data dictionary explaining variable names, coding of values, measurement units etc. After accepting the invitation to collaborate and signing the data transfer agreement, the study authors shared their IPD via secure data transfer portal at the University of Surrey. Data is stored securely on University of Surrey servers in accordance with the European Union's General Data Protection Regulation Act and the UK's Data Protection Act 2018.

TB and MH will take primary responsibility for preparing and extracting outcome data and other characteristics, with MV independently checking extractions for accuracy. Received datasets will be reviewed to assess completeness and checked against the reported

For peer review only

data from relevant study publications. The numbers of participants, overall and by arm, will be confirmed to correspond with the numbers in the study publication. Ranges of values will be inspected for clearly erroneous data. Baseline participant demographic data will be inspected by arm, with the aim of checking that the IPD matches the data reported in the publication, in terms of the numbers of participants with reported data, and overall aggregated values for each variable. Discrepancies will be referred to the study authors for investigation and any remaining discrepancies will be recorded. For each included trial, we will extract information on funding sources and any reported conflicts of interest, and this information will be presented alongside other study characteristics.

Although the main search and initial receipt of IPD occurred before the submission of this protocol for publication, the protocol was registered in PROSPERO (CRD42022332039) beforehand. No data analysis or outcome-driven decisions were made prior to finalizing the protocol, and the methods described here were pre-specified prior to data inspection.

Data harmonization

Once datasets from the individual studies have been checked and outcomes standardised, they will be merged into the final dataset. The final dataset for analysis will be checked again for accuracy by a researcher in the study team.

STATISTICAL ANALYSIS

To finalise the selection of moderator variables, we will determine presence of these variables across the individual data sets and decide on ways to standardise these variables where needed, for example by collapsing categories. Potential moderator variables will be included in the analyses if the variable has at least 40% available data in the final dataset and has been recorded by at least 4 studies.

To investigate the efficacy of MBCT compared to control conditions and test moderating effects of individual patient level and study level characteristics, we will conduct

For peer review only

one-stage and two-stage IPD meta-analyses (IPD-MAs). Random-effects models will be used throughout because we expect high statistical heterogeneity due to variation in control groups (treatment as usual, active controls) and study populations (treatment non-responders/remitters, treatment-resistance, chronic depression).

The main research question of the study (whether MBCT is efficacious compared to control conditions) will be addressed via a maximum of three pairwise comparisons: (1) MBCT versus treatment-as-usual controls, (2) MBCT versus active controls, and (3) MBCT versus all controls.

By default, all analyses will be conducted in a Bayesian framework using JAGS (Just Another Gibbs Sampler)/BUGS (Bayesian analysis Using Gibbs Sampling) [58]. We chose a Bayesian approach because it offers greater flexibility in the parametrization of the (one-stage) IPD-MA models, and because this allows for a more intuitive interpretation of results based on posterior tail probabilities [59] . Across all analyses, findings will be considered "significant" when the credibility interval of the relevant effect does not include zero.

Two-stage random effects meta-analysis

We will first conduct a conventional meta-analysis using data extracted from the published papers. We will calculate Hedges' g to determine the effect sizes of the difference between the intervention and the control conditions reported in the papers. To investigate heterogeneity of effect sizes, we will calculate I^2, the percentage of variation not attributable to sampling error, as well as 95% prediction intervals (PIs) around the pooled effect [60, 61] . Leave-one-out analyses will be conducted to identify potential outliers and/or influential studies. Small-study effects will be investigated visually using funnel plot inspection and the Egger's test for asymmetry. A weakly informative Half-Cauchy (0,0.5) prior will be used for the between-study heterogeneity variance τ^2 [62], and flat normal priors for all other

For peer review only - http://bmjopen.bmj.com/site/about/guidelines.xhtml

parameters. A detailed justification for this prior setup has been derived in previous work [63].

One-stage random effects meta-analysis using IPD

One-stage IPD meta-analysis models will be used as the primary analytic approach. To analyse the effects on common metrics-converted symptom severity scores, an identity-link linear mixed effects model with stratified intercepts and trial-specific (heteroscedastic) error terms will be used. Let y_{ik} be the depressive symptom severity of patient *i* in trial *k* (with *k*=1, …, *K* studies included in the meta-analysis). The one-stage IPD can then be defined as:

$$y_{ik} = \alpha_k + \theta T_{ik} + u_k T_{ik} + \beta_k\left(X_{ik} - \bar{X}_{ik}\right) + \varepsilon_{ik} \qquad (1)$$

$$u_k \sim \mathcal{N}\left(0,\tau_\theta^2\right) \qquad \varepsilon_{ik} \sim \mathcal{N}\left(0,\sigma_k^2\right)$$

Where T_{ik} is the treatment indicator, X_{ik} is the symptom severity score at baseline, $\bar{X}_{ik}$ is the mean of X for each specific trial, α_k is the stratified trial intercept, and u_k is the trial-specific random slope of the average treatment effect θ. For this model, priors mirroring the two-stage approach will be used throughout, with a uniform distribution assigned to σ_k^2.

The same model specification, but with a logit link, will be used to model the effects on binary outcomes, including remission, clinically meaningful improvement and clinically meaningful deterioration based on PHQ-9 thresholds:

$$\log_e\left(\frac{\pi_{ik}}{1-\pi_{ik}}\right) = \alpha_k + \theta T_{ik} + u_k T_{ik} + \beta_k\left(X_{ik} - \bar{X}_{ik}\right) + \varepsilon_{ik} \qquad (2)$$

$$y_{ik} \sim \text{Bern}(\pi_{ik}) \qquad u_k \sim \mathcal{N}\left(0,\tau_\theta^2\right) \qquad \varepsilon_{ik} \sim \mathcal{N}\left(0,\sigma_k^2\right)$$

Where y_{ik} now represents the patient's binary outcome of interest. Numbers needed to treat (*NNT*) corresponding to the estimated SMD values will be calculated using the

For peer review only - http://bmjopen.bmj.com/site/about/guidelines.xhtml
For peer review only

method by Furukawa and Leucht [64], and as the inverse of the absolute risk reduction otherwise.

Moderator analyses

We will explore potential moderators of treatment effect by adding individual participant level and study level treatment-covariate interactions into the main IPD-MA model. To optimise power, these analyses will be done on treatment effects at post-treatment as all studies can provide data for this timepoint.

Missing data

We will record the percentage of individual participant missing data for baseline characteristics and outcomes in each of the studies included in the meta-analyses. All analyses will be conducted according to the "intention-to-treat" principle, so that analyses target a treatment policy estimand [65]. Missing data will be handled using multiple imputation (fully conditional specification; MICE algorithm [66] under the missing at random (MAR) assumption. For the main effectiveness analysis, multilevel two-stage imputation models with heteroscedastic errors will be used to account for the nested data structure [67]. Highly collinear variables will be removed as predictors, as well as variables with systematically missing information ("structural zeros"). A total of m=50 imputation sets will be generated. A separate imputation model will be constructed for each focal moderator, including auxiliary variables with a maximum of 10% missingness. Imputations will be generated separately for each of the treatment groups (intervention, control) to account for treatment-covariate interactions. All analysis models will be fitted in the multiply imputed data. Parameter estimates and test statistics will then be aggregated by mixing the draws from the posterior distribution of each completed dataset [68]. Sensitivity analyses will be performed to compare results using observed and imputed data [69] .

For peer review only

For peer review only - http://bmjopen.bmj.com/site/about/guidelines.xhtml

Subgroup analyses

We plan to examine the efficacy of MBCT and potential moderators of treatment outcomes in subgroups defined by the conventional descriptive categories used by the individual studies to define their populations, distinguishing between studies that recruited patients characterised as treatment non-responders/remitters, treatment-resistant, or as suffering from chronic depression. These analyses will be based on subsamples created from the final IPD dataset.

Sensitivity analyses

As a sensitivity analysis, we will repeat all main effectiveness analyses using equivalent frequentist (one- and two-stage) approaches. Additionally, we will conduct sensitivity analyses to investigate whether sources of heterogeneity affect the overall effect size estimate and robustness of our findings. This will include exploring the role of risk of bias and other potential study level sources of heterogeneity that may become obvious after data have been examined. These analyses will be conducted using a two-stage IPD-MA model.

DISCUSSION

This IPD meta-analysis will provide estimates of efficacy for the use of MBCT in DTD and offer comprehensive information about potential moderators of treatment effects. As such it will contribute to arguments in favour of or against implementing MBCT as a treatment for DTD and may help to decide whether MBCT is a recommendable treatment option for this group. This is important given the need to improve the care and management of DTD and the increasing recognition that psychological therapies can have an integral role in this endeavour. Information about potential moderators of treatment effects will help to refine the understanding of who may or may not benefit from MBCT for DTD.

For peer review only - http://bmjopen.bmj.com/site/about/guidelines.xhtml

We have chosen the emerging concept of DTD as an umbrella because it provides a broad conceptualisation of depression that remains burdensome despite usual treatment attempts. Most available trials have operationalised this construct using narrower criteria such as non-response, non-remission, treatment-resistance, or chronicity, and our inclusion criteria therefore reflect how DTD has been studied to date. These characteristics represent core manifestations of DTD, and their use ensures consistency and feasibility for the planned meta-analysis. We acknowledge, however, that DTD extends beyond these dimensions, for example in its emphasis on functional outcomes, and generalisability of findings to the broader construct should be interpreted with this in mind.

The chosen approach using IPD has several advantages over conventional meta-analyses including the possibility to assess whether individual participant level characteristics influence treatment effects with more specificity and power. Limitations of the approach include the fact that inconsistencies across studies regarding the format and type of variables reported may restrict our ability to investigate moderators of treatment effects and that transformation of outcome measures to a common metric across all studies may come with a small loss of information. To address this latter point, the current study will complement one-stage IPD analyses with a conventional two-stage approach. A more general concern that is often raised for IPD meta-analyses is inclusion bias as it may be difficult to obtain IPD for all selected studies.

This IPD meta-analysis is registered as part of a broader project that examines a wider range of interventions and outcomes, including both beneficial and adverse effects. The present protocol specifies procedures for the comparison between MBCT and control conditions in MBCT trials, as we assume that this comparison is of particular interest given the need to establish interventions for DTD. To our knowledge, this is the first IPD meta-analysis to investigate the effects of MBCT in DTD and assess moderators of treatment effects. As

For peer review only - http://bmjopen.bmj.com/site/about/guidelines.xhtml

evidence from trials of MBCT for DTD has now accumulated to a point where the use of MBCT for DTD can be considered for endorsement in treatment guidelines, findings from this IPD meta-analysis have the potential to inform treatment recommendations and decisions regarding implementation. The results will provide clinicians, healthcare providers and people who are suffering from DTD with information on the characteristics that may make it more or less likely for patients to benefit from this treatment and thus facilitate implementation.

ETHICS AND DISSEMINATION

No local ethical review was necessary following consultation with the Ethics and Governance Board of the University of Surrey. The investigators of the primary trials have local ethical approval for sharing the data. TB and MH will be responsible for oversight of the database and analyses will be conducted by MH and TB. Patient privacy will be ensured by adhering to University of Surrey guidance on data management and storage. Exchange of data is governed by inter-institutional data sharing agreements. All data were pseudonymised (de-identified) before sharing, shared through secure upload facilities and are stored on secure servers. The data set will not be open access. Researchers of good standing may receive access pending on institutional approvals on data transfer.

We will disseminate the work in peer-reviewed journals, provide lay summaries and discuss findings with patient and lived experience groups. Other means of communication to disseminate the results will include conference presentations and social media announcements.

CONTRIBUTIONS

TB, MN, JM, MV and MH conceptualised and designed the study. TB developed the search strategy and contacted the primary authors, who contributed to further refinement of the design and approach of the study. TB, MV and MH are responsible for building the

For peer review only - http://bmjopen.bmj.com/site/about/guidelines.xhtml

database. MH provides statistical expertise and is responsible for data analyses. TB drafted the protocol manuscript with statistical input by MH, which was critically revised by all authors. All authors read and approved the final version. TB serves as the guarantor of the review.

FUNDING STATEMENT

This research received no specific grant from any funding agency in the public, commercial or not-for-profit sectors.

ACKNOWLEDGMENTS

JMM has a ‘Miguel Servet’ research contract from the ISCIII (CP21/00080), and he is grateful to the Spanish CIBER of Epidemiology and Public Health (CBERESP CB22/02/00052< ISCIII) for their support.

COMPETING INTERESTS STATEMENT

TB is the co-author of a book on mindfulness-based interventions and regularly offers workshops on mindfulness-based interventions. He is the co-investigator of a programme grant evaluating an adapted MBCT course for adolescents experiencing depression.

MN is author of a book chapter on mindfulness-based interventions and offers workshops on mindfulness-based interventions.

JM is the director of the Achtsamkeitsinstitut Ruhr (an institute offering mindfulness training) and principal investigator of several DFG (German Science Foundation) research projects. He receives royalties from mindfulness books he has authored.

MV reports no conflicts of interest.

CC is the co-author of a book on mindfulness-based interventions.

JMGW co-developed MBCT, and was founding Director of the University of Oxford Mindfulness Centre. He receives royalties from books describing the MBCT program. He also receives honoraria from lectures and training workshops on mindfulness.

For peer review only - http://bmjopen.bmj.com/site/about/guidelines.xhtml

AC leads a professional practice (Istituto Mente Corpo) that offers courses of mindfulness-based interventions.

SE is the author of book on Mindfulness-Based Cognitive Therapy for the treatment of chronic and treatment-resistant depression.

KD declares no conflicts of interest.

ZS received book royalties from Guilford Press, workshop fees from the Centre for Mindfulness studies, and revenue from online sales at Mindful Noggin Inc., which are all related to his work as a co-founder of Mindfulness-Based Cognitive Therapy (MBCT).

MCM declares no conflicts of interest.

AS is the founder and director of the Radboudumc Expertise Centre for Mindfulness and co-investigator of a number of studies examining the effectiveness and implementation of mindfulness-based interventions for patients with psychological or somatic disorders and health care professionals.

AAF declares no conflicts of interest.

MGT declares no conflicts of interest.

JMM is associated with the University of Oxford Mindfulness Research Centre.

BDD is the lead of the University of Exeter AccEPT clinic, which offers courses of MBCT.

CS is a member of a training organisation that has been commissioned by NHS England to deliver MBCT training across NHS Talking Therapies services in England and co-leads the Sussex Mindfulness Centre. CS is a co-investigator of a programme grant evaluating an adapted MBCT course for adolescents experiencing depression and co-investigator on a grant evaluating an adapted MBCT course for NHS staff.

FAR regularly provides workshops on mindfulness-based interventions.

MR declares no conflicts of interest.

For peer review only - http://bmjopen.bmj.com/site/about/guidelines.xhtml

MH serves as a statistical consultant for HelloBetter/GetOn Institut für Gesundheitstrainings GmbH, a company that implements digital mental health therapeutics in routine care.

REFERENCES

1. Collaborators G 2019 MD. Global, regional, and national burden of 12 mental disorders in 204 countries and territories, 1990–2019: a systematic analysis for the Global Burden of Disease Study 2019. Lancet Psychiatry. 2022;9:137–50. https://doi.org/10.1016/s2215-0366(21)00395-3.

2. McIntyre RS, Alsuwaidan M, Baune BT, Berk M, Demyttenaere K, Goldberg JF, et al. Treatment-resistant depression: definition, prevalence, detection, management, and investigational interventions. World Psychiatry. 2023;22:394–412. https://doi.org/10.1002/wps.21120.

3. Rush AJ, Sackeim HA, Conway CR, Bunker MT, Hollon SD, Demyttenaere K, et al. Clinical research challenges posed by difficult-to-treat depression. Psychol Med. 2022;52:419–32. https://doi.org/10.1017/s0033291721004943.

4. McAllister-Williams RH, Arango C, Blier P, Demyttenaere K, Falkai P, Gorwood P, et al. The identification, assessment and management of difficult-to-treat depression: An international consensus statement. J Affect Disord. 2020;267:264–82. https://doi.org/10.1016/j.jad.2020.02.023.

5. Jaffe DH, Rive B, Denee TR. The humanistic and economic burden of treatment-resistant depression in Europe: a cross-sectional study. BMC Psychiatry. 2019;19:247. https://doi.org/10.1186/s12888-019-2222-4.

6. Bhattacharya R, Shen C, Sambamoorthi U. Excess risk of chronic physical conditions associated with depression and anxiety. BMC Psychiatry. 2014;14. https://doi.org/10.1186/1471-244x-14-10.

7. Moeller SB, Gbyl K, Hjorthøj C, Andreasen M, Austin SF, Buchholtz PE, et al. Treatment of difficult-to-treat depression – clinical guideline for selected interventions. Nord J Psychiatry. 2022;76:177–88. https://doi.org/10.1080/08039488.2021.1952303.

8. Markowitz JC, Wright JH, Peeters F, Thase ME, Kocsis JH, Sudak DM. The Neglected Role of Psychotherapy for Treatment-Resistant Depression. Am J Psychiatry. 2022;179:90–3. https://doi.org/10.1176/appi.ajp.2021.21050535.

9. McHugh RK, Whitton SW, Peckham AD, Welge JA, Otto MW. Patient Preference for Psychological vs Pharmacologic Treatment of Psychiatric Disorders: A Meta-Analytic Review. J Clin Psychiatry. 2013;74:595–602. https://doi.org/10.4088/jcp.12r07757.

For peer review only

For peer review only - http://bmjopen.bmj.com/site/about/guidelines.xhtml

10. Segal ZV, Williams JMG, Teasdale J. Mindfulness-Based Cognitive Therapy for Depression. 2013.

11. Barnhofer T, Dunn BD, Strauss C, Ruths FA, Barrett B, Ryan M, et al. Mindfulness-based cognitive therapy versus treatment as usual after non-remission with NHS Talking Therapies high-intensity psychological therapy for depression: a UK-based clinical effectiveness and cost-effectiveness randomised, controlled, superiority trial. Lancet Psychiatry. 2025;12:433–46. https://doi.org/10.1016/s2215-0366(25)00105-1.

12. Kuyken W, Warren FC, Taylor RS, Whalley B, Crane C, Bondolfi G, et al. Efficacy of Mindfulness-Based Cognitive Therapy in Prevention of Depressive Relapse: An Individual Patient Data Meta-analysis From Randomized Trials. Jama Psychiat. 2016;73:565. https://doi.org/10.1001/jamapsychiatry.2016.0076.

13. Ma SH, Teasdale JD. Mindfulness-Based Cognitive Therapy for Depression: Replication and Exploration of Differential Relapse Prevention Effects. J Consult Clin Psych. 2004;72:31–40. https://doi.org/10.1037/0022-006x.72.1.31.

14. Teasdale JD, Segal ZV, Williams JMG, Ridgeway VA, Soulsby JM, Lau MA. Prevention of relapse/recurrence in major depression by mindfulness-based cognitive therapy. J Consult Clin Psych. 2000;68:615–23. https://doi.org/10.1037/0022-006x.68.4.615.

15. Creswell JD, Lindsay EK. How Does Mindfulness Training Affect Health? A Mindfulness Stress Buffering Account. Current Directions in Psychological Science. 2014;23. https://doi.org/10.1177/0963721414547415.

16. Lindsay EK, Creswell DJ. Mechanisms of mindfulness training: Monitor and Acceptance Theory (MAT). Clinical Psychology Review. 2017;51. https://doi.org/10.1016/j.cpr.2016.10.011.

17. Eisendrath SJ, Delucchi K, Bitner R, Fenimore P, Smit M, McLane M. Mindfulness-Based Cognitive Therapy for Treatment-Resistant Depression: A Pilot Study. Psychother Psychosom. 2008;77:319–20. https://doi.org/10.1159/000142525.

18. Barnhofer T, Crane C, Hargus E, Amarasinghe M, Winder R, Williams JMG. Mindfulness-based cognitive therapy as a treatment for chronic depression: A preliminary study. Behav Res Ther. 2009;47:366–73. https://doi.org/10.1016/j.brat.2009.01.019.

19. Eisendrath SJ, Gillung E, Delucchi KL, Segal ZV, Nelson JC, McInnes LA, et al. A Randomized Controlled Trial of Mindfulness-Based Cognitive Therapy for Treatment-Resistant Depression. Psychother Psychosom. 2016;85:99–110. https://doi.org/10.1159/000442260.

20. Cladder-Micus MB, Speckens AEM, Vrijsen JN, Donders ART, Becker ES, Spijker J. Mindfulness-based cognitive therapy for patients with chronic, treatment-resistant depression: A pragmatic randomized controlled trial. Depress Anxiety. 2018;35:914–24. https://doi.org/10.1002/da.22788.

21. Michalak J, Schultze M, Heidenreich T, Schramm E. A randomized controlled trial on the efficacy of mindfulness-based cognitive therapy and a group version of cognitive behavioral

For peer review only

analysis system of psychotherapy for chronically depressed patients. J Consult Clin Psych. 2015;83:951–63. https://doi.org/10.1037/ccp0000042.

22. Chiesa A, Castagner V, Andrisano C, Serretti A, Mandelli L, Porcelli S, et al. Mindfulness-based cognitive therapy vs. psycho-education for patients with major depression who did not achieve remission following antidepressant treatment. Psychiat Res. 2015;226:474–83. https://doi.org/10.1016/j.psychres.2015.02.003.

23. Garcia A, Yáñez AM, Bennasar-Veny M, Navarro C, Salva J, Ibarra O, et al. Efficacy of an adjuvant non-face-to-face multimodal lifestyle modification program for patients with treatment-resistant major depression: A randomized controlled trial. Psychiatry Res. 2023;319:114975. https://doi.org/10.1016/j.psychres.2022.114975.

24. Foroughi A, Sadeghi K, Parvizifard A, Moghadam AP, Davarinejad O, Farnia V, et al. The effectiveness of mindfulness-based cognitive therapy for reducing rumination and improving mindfulness and self-compassion in patients with treatment-resistant depression. Trends Psychiatry Psychotherapy. 2020;42:138–46. https://doi.org/10.1590/2237-6089-2019-0016.

25. Mathew T, Nordström K. Comparison of One-Step and Two-Step Meta-Analysis Models Using Individual Patient Data. Biom J. 2010;52:271–87. https://doi.org/10.1002/bimj.200900143.

26. Clarke MJ. Individual patient data meta-analyses. Best Pr Res Clin Obstet Gynaecol. 2005;19:47–55. https://doi.org/10.1016/j.bpobgyn.2004.10.011.

27. Riley RD, Lambert PC, Abo-Zaid G. Meta-analysis of individual participant data: rationale, conduct, and reporting. BMJ. 2010;340 feb05 1:c221. https://doi.org/10.1136/bmj.c221.

28. Jones AP, Riley RD, Williamson PR, Whitehead A. Meta-analysis of individual patient data versus aggregate data from longitudinal clinical trials. Clin Trials. 2009;6:16–27. https://doi.org/10.1177/1740774508100984.

29. Cooper H, Patall EA. The Relative Benefits of Meta-Analysis Conducted With Individual Participant Data Versus Aggregated Data. Psychol Methods. 2009;14:165–76. https://doi.org/10.1037/a0015565.

30. Stewart LA, Clarke M, Rovers M, Riley RD, Simmonds M, Stewart G, et al. Preferred Reporting Items for a Systematic Review and Meta-analysis of Individual Participant Data: The PRISMA-IPD Statement. Jama. 2015;313:1657–65. https://doi.org/10.1001/jama.2015.3656.

31. Löfholm CA, Brännström L, Olsson M, Hansson K. Treatment-as-usual in effectiveness studies: What is it and does it matter? Int J Soc Welf. 2013;22:25–34. https://doi.org/10.1111/j.1468-2397.2012.00870.x.

32. Kabat-Zinn J. Mindfulness-Based Interventions in Context: Past, Present, and Future. Clinical Psychology: Science and Practice. 2003;10:144–56. https://doi.org/10.1093/clipsy.bpg016.

For peer review only

For peer review only - http://bmjopen.bmj.com/site/about/guidelines.xhtml

33. Association AP. Diagnostic and statistical manual of mental disorders (5th ed.). Arlington, VA: Author; 2013.

34. Association AP. Diagnostic and statistical manual of mental disorders (4th ed.). Washington, DC: Author; 2000.

35. Korten NCM, Comijs HC, Lamers F, Penninx BWJH. Early and late onset depression in young and middle aged adults: Differential symptomatology, characteristics and risk factors? J Affect Disord. 2012;138:259–67. https://doi.org/10.1016/j.jad.2012.01.042.

36. Devanand DP, Adorno E, Cheng J, Burt T, Pelton GH, Roose SP, et al. Late onset dysthymic disorder and major depression differ from early onset dysthymic disorder and major depression in elderly outpatients. J Affect Disord. 2004;78:259–67. https://doi.org/10.1016/s0165-0327(02)00307-5.

37. Wahl I, Löwe B, Bjorner JB, Fischer F, Langs G, Voderholzer U, et al. Standardization of depression measurement: a common metric was developed for 11 self-report depression measures. J Clin Epidemiology. 2014;67:73–86. https://doi.org/10.1016/j.jclinepi.2013.04.019.

38. Rush AJ, Trivedi MH, Ibrahim HM, Carmody TJ, Arnow B, Klein DN, et al. The 16-Item quick inventory of depressive symptomatology (QIDS), clinician rating (QIDS-C), and self-report (QIDS-SR): a psychometric evaluation in patients with chronic major depression. Biol Psychiatry. 2003;54:573–83. https://doi.org/10.1016/s0006-3223(02)01866-8.

39. Kroenke K, Spitzer RL, Williams JBW. The PHQ-9: Validity of a brief depression severity measure. J Gen Intern Med. 2001;16:606–13. https://doi.org/10.1046/j.1525-1497.2001.016009606.x.

40. Löwe B, Unützer J, Callahan CM, Perkins AJ, Kroenke K. Monitoring Depression Treatment Outcomes With the Patient Health Questionnaire-9. Med Care. 2004;42:1194–201. https://doi.org/10.1097/00005650-200412000-00006.

41. Hudgens S, Floden L, Blackowicz M, Jamieson C, Popova V, Fedgchin M, et al. Meaningful Change in Depression Symptoms Assessed with the Patient Health Questionnaire (PHQ-9) and Montgomery-Åsberg Depression Rating Scale (MADRS) Among Patients with Treatment Resistant Depression in Two, Randomized, Double-blind, Active-controlled Trials of Esketamine Nasal Spray Combined With a New Oral Antidepressant. J Affect Disord. 2021;281:767–75. https://doi.org/10.1016/j.jad.2020.11.066.

42. Turkoz I, Alphs L, Singh J, Jamieson C, Daly E, Shawi M, et al. Clinically meaningful changes on depressive symptom measures and patient-reported outcomes in patients with treatment-resistant depression. Acta Psychiatr Scand. 2021;143:253–63. https://doi.org/10.1111/acps.13260.

43. Hölzel L, Härter M, Reese C, Kriston L. Risk factors for chronic depression — A systematic review. J Affect Disorders. 2011;129:1–13. https://doi.org/10.1016/j.jad.2010.03.025.

For peer review only - http://bmjopen.bmj.com/site/about/guidelines.xhtml

44. Seedat S, Scott KM, Angermeyer MC, Berglund P, Bromet EJ, Brugha TS, et al. Cross-National Associations Between Gender and Mental Disorders in the World Health Organization World Mental Health Surveys. Arch Gen Psychiatry. 2009;66:785–95. https://doi.org/10.1001/archgenpsychiatry.2009.36.

45. Buckman JEJ, Saunders R, Stott J, Arundell L-L, O'Driscoll C, Davies MR, et al. Role of age, gender and marital status in prognosis for adults with depression: An individual patient data meta-analysis. Epidemiology Psychiatr Sci. 2021;30:e42. https://doi.org/10.1017/s2045796021000342.

46. Trivedi MH, Rush AJ, Wisniewski SR, Nierenberg AA, Warden D, Ritz L, et al. Evaluation of Outcomes With Citalopram for Depression Using Measurement-Based Care in STARD: Implications for Clinical Practice. Am J Psychiatry. 2006;163:28–40. https://doi.org/10.1176/appi.ajp.163.1.28.

47. Oosten AJ van, Mens K van, Blonk RWB, Burdorf A, Tiemens B. The relationship between having a job and the outcome of brief therapy in patients with common mental disorders. BMC Psychiatry. 2023;23:910. https://doi.org/10.1186/s12888-023-05418-z.

48. Nanni V, Uher R, Danese A. Childhood Maltreatment Predicts Unfavorable Course of Illness and Treatment Outcome in Depression: A Meta-Analysis. Am J Psychiat. 2012;169:141–51. https://doi.org/10.1176/appi.ajp.2011.11020335.

49. Klein DN, Schatzberg AF, McCullough JP, Dowling F, Goodman D, Howland RH, et al. Age of onset in chronic major depression: relation to demographic and clinical variables, family history, and treatment response. J Affect Disord. 1999;55:149–57. https://doi.org/10.1016/s0165-0327(99)00020-8.

50. Bromberger JT, Schott L, Kravitz HM, Joffe H. Risk factors for major depression during midlife among a community sample of women with and without prior major depression: are they the same or different? Psychol Med. 2015;45:1653–64. https://doi.org/10.1017/s0033291714002773.

51. Myklebost SB, Gjestad R, Inal Y, Hammar Å, Nordgreen T. Predictors of Treatment Response to an Internet-Delivered Intervention Targeting Residual Cognitive Symptoms After Major Depressive Disorder. Front Psychiatry. 2022;13:795698. https://doi.org/10.3389/fpsyt.2022.795698.

52. Courtney DB, Watson P, Krause KR, Chan BWC, Bennett K, Gunlicks-Stoessel M, et al. Predictors, Moderators, and Mediators Associated With Treatment Outcome in Randomized Clinical Trials Among Adolescents With Depression. JAMA Netw Open. 2022;5:e2146331. https://doi.org/10.1001/jamanetworkopen.2021.46331.

53. Cuijpers P, Sijbrandij M, Koole SL, Andersson G, Beekman AT, Reynolds CF. Adding psychotherapy to antidepressant medication in depression and anxiety disorders: a meta-analysis. World Psychiatry. 2014;13:56–67. https://doi.org/10.1002/wps.20089.

54. DeRubeis RJ, Hollon SD, Amsterdam JD, Shelton RC, Young PR, Salomon RM, et al. Cognitive Therapy vs Medications in the Treatment of Moderate to Severe Depression. Arch Gen Psychiatry. 2005;62:409–16. https://doi.org/10.1001/archpsyc.62.4.409.

For peer review only - http://bmjopen.bmj.com/site/about/guidelines.xhtml

55. Button KS, Wiles NJ, Lewis G, Peters TJ, Kessler D. Factors associated with differential response to online cognitive behavioural therapy. Soc Psychiatry Psychiatr Epidemiology. 2012;47:827–33. https://doi.org/10.1007/s00127-011-0389-1.

56. Furlan AD, Pennick V, Bombardier C, Tulder M van, Group EB Cochrane Back Review. 2009 Updated Method Guidelines for Systematic Reviews in the Cochrane Back Review Group. Spine. 2009;34:1929–41. https://doi.org/10.1097/brs.0b013e3181b1c99f.

57. Sterne JAC, Savović J, Page MJ, Elbers RG, Blencowe NS, Boutron I, et al. RoB 2: a revised tool for assessing risk of bias in randomised trials. Bmj. 2019;366:l4898. https://doi.org/10.1136/bmj.l4898.

58. Lunn D, Jackson C, Best N, Thomas A, Spiegelhalter D. The BUGS Book, A Practical Introduction to Bayesian Analysis. 2019. https://doi.org/10.1201/b13613.

59. Goligher EC, Heath A, Harhay MO. Bayesian statistics for clinical research. Lancet. 2024;404:1067–76. https://doi.org/10.1016/s0140-6736(24)01295-9.

60. Higgins JPT, Thompson SG, Deeks JJ, Altman DG. Measuring inconsistency in meta-analyses. BMJ. 2003;327:557–60. https://doi.org/10.1136/bmj.327.7414.557.

61. Higgins JPT, Thompson SG. Quantifying heterogeneity in a meta-analysis. Stat Med. 2002;21:1539–58. https://doi.org/10.1002/sim.1186.

62. Harrer M, Cuijpers P, Furukawa TA, Ebert DD. Doing Meta-Analysis with R, A Hands-On Guide. 2022. https://doi.org/10.1201/9781003107347.

63. Harrer M. Heterogeneity Prior Justification. 2022. https://osf.io/3tfm6. Accessed 16 May 2025.

64. Furukawa TA, Leucht S. How to Obtain NNT from Cohen's d: Comparison of Two Methods. PLoS ONE. 2011;6:e19070. https://doi.org/10.1371/journal.pone.0019070.

65. Han S, Zhou X. Defining estimands in clinical trials: A unified procedure. Stat Med. 2023;42:1869–87. https://doi.org/10.1002/sim.9702.

66. Buuren van, Groothuis-Oudshoorn K. mice: multivariate imputation by chained equations in R. Journal of Statistical Software. 2011;45:1–67.

67. Resche-Rigon M, White IR. Multiple imputation by chained equations for systematically and sporadically missing multilevel data. Stat Methods Méd Res. 2018;27:1634–49. https://doi.org/10.1177/0962280216666564.

68. Zhou X, Reiter JP. A Note on Bayesian Inference After Multiple Imputation. Am Stat. 2010;64:159–63. https://doi.org/10.1198/tast.2010.09109.

69. Burgess S, White IR, Resche-Rigon M, Wood AM. Combining multiple imputation and meta-analysis with individual participant data. Stat Med. 2013;32:4499–514. https://doi.org/10.1002/sim.5844.

For peer review only

For peer review only - http://bmjopen.bmj.com/site/about/guidelines.xhtml

For peer review only

For peer review only - http://bmjopen.bmj.com/site/about/guidelines.xhtml

APPENDIX

Search Terms

Database	Search Term
Web of Science	(“MDE” OR “major depressive episode” OR “MDD” OR “major depressive disorder” OR “major depression” OR “depression” OR “dysthymia” OR “dysthymic”) AND (“chronic” OR “persistent” OR “treatment-resistant” OR “non-responders” OR “non-response” OR “non-remitted” OR “dysthymia” OR “dysthymic”) AND (“MBCT” OR "mindfulness-based cognitive therapy") AND (“randomized controlled trial” OR “randomised controlled trial” OR “randomized-controlled trial” OR “randomised-controlled trial” OR “RCT” OR “randomized” or “randomised” OR “randomly allocated” OR “randomly assigned”)
Scopus	(“MDE” OR “major depressive episode” OR “MDD” OR “major depressive disorder” OR “major depression” OR “depression” OR “dysthymia” OR “dysthymic”) AND (“chronic” OR “persistent” OR “treatment-resistant” OR “non-responders” OR “non-response” OR “non-remitted” OR “dysthymia” OR “dysthymic”) AND (“MBCT” OR "mindfulness-based cognitive therapy") AND (“randomized controlled trial” OR “randomised controlled trial” OR “randomized-controlled trial” OR “randomised-controlled trial” OR “RCT” OR “randomized” or “randomised” OR “randomly allocated” OR “randomly assigned”)
PubMed	(“MDE” OR “major depressive episode” OR “MDD” OR “major depressive disorder” OR “major depression” OR “depression” OR “dysthymia” OR “dysthymic”) AND (“chronic” OR “persistent” OR “treatment-resistant” OR “non-responders” OR “non-response” OR “non-remitted” OR “dysthymia” OR “dysthymic”) AND (“MBCT” OR "mindfulness-based cognitive therapy") AND (“randomized controlled trial” OR “randomised controlled trial” OR “randomized-controlled trial” OR “randomised-controlled trial” OR “RCT” OR “randomized” or “randomised” OR “randomly allocated” OR “randomly assigned”)
PsycInfo	(“MDE” OR “major depressive episode” OR “MDD” OR “major depressive disorder” OR “major depression” OR “depression” OR “dysthymia” OR “dysthymic”) **AND** (“chronic” OR “persistent” OR “treatment-resistant” OR “non-responders” OR “non-response” OR “non-remitted” OR “dysthymia” OR “dysthymic”) **AND** (“MBCT” OR "mindfulness-based cognitive therapy") **AND** (“randomized controlled trial” OR “randomised controlled trial” OR “randomized-controlled trial” OR “randomised-controlled trial” OR “RCT” OR “randomized” or “randomised” OR “randomly allocated” OR “randomly assigned”)
Embase	(‘MDE’ OR ‘major depressive episode’ OR ‘MDD’ OR ‘major depressive disorder’ OR ‘major depression’ OR ‘depression’ OR ‘dysthymia’ OR ‘dysthymic’) **AND** (‘chronic’ OR ‘persistent’ OR ‘treatment-resistant’ OR ‘non-responders’ OR ‘non-response’ OR ‘non-remitted’ OR ‘dysthymia’ OR ‘dysthymic’) **AND** (‘MBCT’ OR ‘mindfulness-based cognitive therapy’) **AND** (‘randomized controlled trial’ OR ‘randomised controlled trial’ OR ‘randomized-controlled trial’ OR ‘randomised-controlled trial’ OR ‘RCT’ OR ‘randomized’ or ‘randomised’ OR ‘randomly allocated’ OR ‘randomly assigned’)

For peer review only - http://bmjopen.bmj.com/site/about/guidelines.xhtml

Cochrane Controlled Trials	(“MDE” OR “major depressive episode” OR “MDD” OR “major depressive disorder” OR “major depression” OR “depression” OR “dysthymia” OR “dysthymic”) AND (“chronic” OR “persistent” OR “treatment-resistant” OR “non-responders” OR “non-response” OR “non-remitted” OR “dysthymia” OR “dysthymic”) AND (“MBCT” OR "mindfulness-based cognitive therapy") AND (“randomized controlled trial” OR “randomised controlled trial” OR “randomized-controlled trial” OR “randomised-controlled trial” OR “RCT” OR “randomized” or “randomised” OR “randomly allocated” OR “randomly assigned”)

For peer review only

For peer review only - http://bmjopen.bmj.com/site/about/guidelines.xhtml

BMJ Open

Efficacy and Moderators of Mindfulness-Based Cognitive Therapy (MBCT) in 'Difficult to Treat' Depression: Protocol for a Systematic Review and Individual Patient Data Meta-Analysis of Randomised Controlled Trials

Journal:	*BMJ Open*
Manuscript ID	bmjopen-2025-106350.R2
Article Type:	Protocol
Date Submitted by the Author:	08-Oct-2025
Complete List of Authors:	Barnhofer, Thorsten; University of Surrey,<br>Niemi, Maria; Karolinska Institutet, Department of Global Public Health<br>Michalak, Johannes; Witten/Herdecke University, Department of Psychology and Psychotherapy<br>Velana, Maria; Witten/Herdecke University, Department of Psychology and Psychotherapy<br>Williams, J Mark; University of Oxford, Department of Psychiatry<br>Chiesa, Alberto; Università di Bologna<br>Eisendrath, Stuart; University of California at San Francisco<br>Delucchi, Kevin; University of California at San Francisco<br>Segal, Zindel; University of Toronto - Scarborough, Graduate Department of Psychological Clinical Science<br>Cladder-Micus, Mira; Radboud University Nijmegen<br>Speckens, Anne; Radboudumc Instituut voor Wetenschappelijk Onderwijs en Opleidingen, Psychiatry<br>Foroughi, Ali Akbar; Kermanshah University of Medical Sciences (KUMS), Department of Clinical Psychology<br>García-Toro, M; Research Network on Chronicity Primary Care and Health Promotion; University of the Balearic Islands; Health Research Institute of the Balearic Islands; University of the Balearic Islands, Department of Medicine<br>Montero-Marin, Jesus; Research & Innovation Unit Parc Sanitari Sant Joan de Déu; University of Oxford, Department of Psychiatry; Consortium for Biomedical Research in Epidemiology and Public Health<br>dunn, barney; University of Exeter<br>Strauss, Clara; Sussex Partnership NHS Foundation Trust; University of Sussex<br>Ruths, Florian; South London and Maudsley NHS Foundation Trust<br>Ryan, Mary; Royal College of Psychiatrists<br>Harrer, Mathias; Technical University of Munich, Department of Psychiatry and Psychotherapy; World Health Organization, Department of Clinical, Neuro and Developmental Psychology; Vrije Universiteit
**Primary Subject Heading**:	Mental health
Secondary Subject Heading:	Mental health

Keywords:	Meta-Analysis, Depression & mood disorders < PSYCHIATRY, Mindfulness

SCHOLARONE™
Manuscripts

Title

Efficacy and Moderators of Mindfulness-Based Cognitive Therapy (MBCT) in 'Difficult to Treat' Depression: Protocol for a Systematic Review and Individual Patient Data Meta-Analysis of Randomised Controlled Trials

Running Title

MBCT in 'Difficult to Treat' Depression

Authors and Affiliations

Thorsten Barnhofer [1], Maria Niemi [2, *], Johannes Michalak [3], Maria Velana [3], Catherine Crane [4], J. Mark G. Williams [4], Alberto Chiesa [5], Stuart Eisendrath [6], Kevin Delucchi [6], Zindel V. Segal [7], Mira Cladder-Micus [8], Anne Speckens [9], Ali Akbar Foroughi [10], Mauro Garcia-Toro [11, 12, 13, 14], Jesus Montero-Marin [15, 4, 16], Barnaby D. Dunn [17], Clara Strauss [18, 19], Florian A. Ruths [20], Mary Ryan [21], Mathias Harrer [22, 23, 24]

[1] School of Psychology, University of Surrey, Guildford, UK, t.barnhofer@surrey.ac.uk

[2] Department of Global Public Health, Karolinska Institutet, Stockholm, Sweden, maria.niemi@ki.se

[3] Department of Psychology and Psychotherapy, Witten/Herdecke University, Witten, Germany, Maria.Velana@uni-wh.de , Johannes.Michalak@uni-wh.de

[4] Department of Psychiatry, University of Oxford, Oxford, UK, catherine.crane@psych.ox.ac.uk, mark.williams@psych.ox.ac.uk, jesus.monteromarin@psych.ox.ac.uk

[5] Institute of Psychiatry, Università di Bologna, Bologna, Italy, chiesa@scuola-spc.it

[6] Weill Institute for Neurosciences, University of California at San Francisco, San Francisco, USA, stuart.eisendrath@ucsf.edu, kevin.delucchi@ucsf.edu

For peer review only - http://bmjopen.bmj.com/site/about/guidelines.xhtml

[7] Graduate Department of Psychological Clinical Science, University of Toronto - Scarborough, Toronto, Canada, zindel.segal@utoronto.ca

[8] Behavioural Science Institute, Radboud University Nijmegen, Nijmegen, The Netherlands, Mira.Cladder-Micus@radboudumc.nl

[9] Department of Psychiatry, Radboudumc, Nijmegen, The Netherlands, anne.speckens@radboudumc.nl

[10] Department of Clinical Psychology, Kermanshah University of Medical Sciences (KUMS), Hermanshah, Iran, foroughi_2002@yahoo.com

[11] Research Network on Chronicity, Primary Care and Health Promotion (RICAPPS), Carlos III Health Institute, Madrid, Spain

[12] University Institute of Health Science Research (IUNICS), University of the Balearic Islands, Spain, mauro.garcia@uib.es

[13] Health Research Institute of the Balearic Islands (IdISBa), Palma, Spain

[14] Department of Medicine, University of the Balearic Islands, Palma, Spain

[15] Research & Innovation Unit, Parc Sanitari Sant Joan de Déu, Sant Boi de Llobregat, Barcelona, Spain

[16] Consortium for Biomedical Research in Epidemiology and Public Health (CIBER Epidemiology and Public Health-CIBERESP), Madrid, Spain

[17] Mood Disorders Centre, University of Exeter, Exeter, UK, b.d.dunn@exeter.ac.uk

[18] Sussex Partnership NHS Foundation Trust, Hove, UK, clara.strauss@nhs.net

[19] Sussex Mental Health Research Centre, University of Sussex, Brighton, UK

[20] South London and Maudsley NHS Foundation Trust, London, UK, Florian.Ruths@slam.nhs.uk

[21] Royal College of Psychiatrists, London, UK, mary@highbag.co.uk

For peer review only - http://bmjopen.bmj.com/site/about/guidelines.xhtml

[22] Department of Psychiatry and Psychotherapy, School of Medicine and Health, Technical University of Munich, Munich, Germany

[23] Department of Clinical, Neuro and Developmental Psychology, World Health Organization

[24] Collaborating Center for Research and Dissemination of Psychological Interventions, Amsterdam, Public Health Research Institute, Vrije Universiteit, Amsterdam, The Netherlands, mathias.harrer@tum.de

* Corresponding author:

Associate Professor Maria Niemi, Department of Global Public Health, Karolinska Institutet, Stockholm, Sweden, maria.niemi@ki.se

Word Count

Abstract: 300

Main text: 4110

Keywords

Major Depression, Difficult-to-Treat, Mindfulness-Based Cognitive Therapy, Independent Patient Data, Meta-Analysis

For peer review only

ABSTRACT

Introduction: About 30% of depressed patients suffer from a protracted course in which the disorder continues to cause significant burden despite treatment efforts. While originally developed for relapse prevention, Mindfulness-Based Cognitive Therapy (MBCT) has increasingly been investigated in depressed patients with such 'difficult-to-treat' courses. This is a protocol for an individual participant data (IPD) meta-analysis aiming to determine efficacy and potential moderators of MBCT treatment effects in this group based on evidence from randomised controlled trials.

Methods and analysis: Systematic searches in PubMed, Web of Science, Scopus, PsycINFO, EMBASE, and the Cochrane Controlled Trials Register for randomised controlled trials were completed on 17 June 2024. Authors of identified studies have contributed IPD and data extractions have been completed. An update search will be conducted immediately before the start of data analyses. We will investigate the following outcomes: (a) self-reported and observer-reported severity of depression symptomatology, (b) remission, (c) clinically meaningful improvement and deterioration. One-stage and two-stage IPD meta-analyses will be conducted with one-stage models using the observed IPD from all studies simultaneously as the primary approach. One-stage IPD models will include stratified study intercepts and error terms as well as random effects to capture between-study heterogeneity. Moderator analyses will test treatment-covariate interactions for both individual patient level and study level characteristics.

Ethics and dissemination: The results will inform understanding of the use of MBCT in patients with current 'difficult-to-treat' depression and will contribute to arguments in favour of or against implementing MBCT as a treatment for this group. They will be published in a peer reviewed journal and made available to stakeholders in accessible formats. No local ethical review was necessary following consultation with the Ethics and Governance Board

For peer review only - http://bmjopen.bmj.com/site/about/guidelines.xhtml

of the University of Surrey. Guidance on patient data storage and management will be adhered to throughout.

PROSPERO registration number: CRD42022332039

For peer review only

For peer review only - http://bmjopen.bmj.com/site/about/guidelines.xhtml

STRENGTHS AND LIMITATIONS OF THIS STUDY

- The chosen approach using IPD has several advantages over conventional meta-analyses including the possibility to assess whether individual participant level characteristics influence treatment effects with more specificity and power
- Limitations of the approach include the fact that inconsistencies across studies regarding the format and type of variables reported may restrict our ability to investigate moderators of treatment effects
- Transformation of outcome measures to a common metric across all studies may come with a small loss of information.

For peer review only - http://bmjopen.bmj.com/site/about/guidelines.xhtml

INTRODUCTION

Major Depressive Disorder (MDD) is highly prevalent, debilitating [1] , and in about 30% of patients takes a recurrent or chronic course in which established treatments fail to bring sustained remission [2] . Treatment resistance has traditionally been defined in narrow operational terms (e.g. non-response after two adequate pharmacological treatments), but such definitions vary across studies and exclude many patients with persistent, impairing depression. The broader construct of difficult-to-treat depression (DTD) was recently introduced to capture the true spectrum within a single heuristic, providing a clinically meaningful framework that better reflects the overlap between these groups and the challenges faced in practice [3, 4]. Defined as “depression that continues to cause significant burden despite usual treatment efforts”, such courses are associated with continuing functional impairment, reduced quality of life, and significantly increased risk for chronic physical and neurodegenerative disorders [5, 6] . Finding effective treatments for DTD remains an important challenge for depression research [7] .

While treatment approaches for DTD have traditionally been dominated by pharmacological strategies [8] , it is now increasingly acknowledged that effective management requires a broader emphasis on working with symptoms, maximising function and minimizing burden [4] . Psychological interventions have an important contribution to make in this context [9] and emerging evidence suggests that Mindfulness-Based Cognitive Therapy (MBCT [10]), an eight-week treatment that combines elements of cognitive therapy with mindfulness meditation, may be suited for this purpose [11] . Using a mental training approach, the intervention aims to provide patients with portable skills that enable them to disengage from habitual maladaptive responses and respond more adaptively to negative mood and stress.

For peer review only - http://bmjopen.bmj.com/site/about/guidelines.xhtml

MBCT was originally introduced for the prevention of relapse in patients who are currently in remission and has been proven to be effective for this purpose [12] , particularly in patients with highly recurrent courses (a use that is endorsed by treatment guidelines internationally). Early trials restricted the intervention to patients who were currently in full remission as it was assumed that intensive mindfulness practice may be too demanding for those who are suffering from current symptoms [13, 14] . However, as patients who are at high risk of relapse are likely to show residual symptoms between episodes, this restriction was later loosened, and analyses across studies for relapse prevention show that treatment effects increase, rather than decrease, with level of residual symptoms [12] . From a theoretical perspective, this observation is in line with the view that mindfulness skills serve to buffer the effects of negative mood and stress [15, 16] , which implies that effects should become more visible under conditions of higher symptoms or stress.

Based on this reasoning, research has extended the use of MBCT to patients with current depression described as 'difficult-to-treat'. Preliminary studies investigating MBCT in treatment-resistant and chronic depression were published in 2008 [17] and 2009 [18] and several definitive randomised controlled trials have been conducted since then [19–24] , suggesting that evidence has accumulated to a point where the use of MBCT as a treatment for patients with current DTD can now be considered for guideline endorsement and wider implementation. However, conclusions from individual studies are contrained by heterogeneous definitions and settings, variable comparators and follow-up durations, inconsistent outcome reporting, and limited power for moderator analyses. It seems timely therefore to bring data from existing randomised controlled trials together and analyse outcome across studies.

For this purpose, we have pooled individual patient data (IPD) from these studies to conduct meta-analyses with one-stage random effects meta-analysis of independent patient

For peer review only

data (IPD) as our primary approach given its advantages over conventional aggregate data meta-analyses [25–28] . This IPD meta-analysis will apply a consistent DTD framework, harmonise outcomes, estimate overall effects using one-stage models, and test prespecified patient-level moderators, with comprehensive risk-of-bias and small-study assessments. The main aim of the analyses is to establish efficacy of MBCT for patients with DTD as compared to the control conditions utilised in the existing trials. Given the need for both effective symptom reduction and the maintenance of gains, we will aim to determine immediate effects at the end of the eight-week MBCT intervention (post-treatment) and effects in the longer-term after patients have finished the intervention and are free to continue engaging in mindfulness practice by themselves (follow-up).

An important advantage of IPD meta-analysis is that it allows to examine moderator or subgroup questions across the pooled sample and can thus serve to overcome limitations in sample size and power for answering these questions in the original studies [29] . Information about potential moderators of treatment effects, such as severity of depression at entry to treatment or a history of bipolar disorder, is important as it can potentially help with implementation by providing more precise information about relative indications or contraindications of the intervention. A further aim of this IPD meta-analysis is therefore to explore effects of potential individual patient level (e.g. sociodemographic and clinical characteristics) and study level (e.g. delivery format) moderators of treatment effects in the pooled data set.

As the clinical concept of DTD does not link to an objective way of assessment, the current meta-analysis will include studies that have used a range of conventional definitions falling under the wider concept of DTD, including selecting participants based on treatment non-response/remission, treatment resistance or the presence of chronic depression. Subgroup analyses will allow us to determine the extent to which effects found in the aggregated data

For peer review only - http://bmjopen.bmj.com/site/about/guidelines.xhtml

generalise across these conventional categories. Given that DTD reflects a wider construct encompassing a large and heterogeneous group of patients with different characteristics and different treatment histories, we will aim to carefully describe the sample both on the meta- and the study-level.

METHODS

General approach

This IPD meta-analysis is registered as part of a broader project on PROSPERO (registration number: CRD42022332039; date of registration in PROSPERO: 24 May 2022) and any key changes or amendments will be documented there. In addition to the direct comparisons between MBCT and control conditions, the wider project also includes studies of an individual psychotherapeutic intervention, Cognitive Behavioral Analysis System of Psychotherapy, and considers a wider range of outcomes, including both beneficial and adverse treatment effects. The current protocol specifies procedures for the comparison between MBCT and the control conditions utilized in the existing MBCT trials only as we assume that this comparison is of particular interest given the need for establishing further treatment options for DTD. The moderator and sensitivity analyses described here were prespecified in this protocol prior to data analysis but were not detailed in the initial PROSPERO entry. They are included to address theoretically and clinically relevant questions, and all analyses (including null results) will be reported to minimise selective reporting. The protocol is registered retrospectively as searches to identify eligible papers were already conducted in 2024, with the last search having been performed on 17 June 2024 and an update search planned immediately before the start of data analysis. Corresponding authors of the selected studies were contacted and asked to provide raw data from their studies. The Preferred Reporting Items for Systematic Reviews and Meta-Analyses IPD statement [30] will be followed for the reporting of this study.

For peer review only - http://bmjopen.bmj.com/site/about/guidelines.xhtml

Eligibility criteria

Types of studies to be included

This IPD meta-analysis will include only peer-reviewed RCTs, published in English. There will be no restriction on the type of recruitment settings. The restriction to English-language publications is due to feasibility constraints and is recognised as a potential source of language bias.

Type of intervention

Included studies will have investigated the effects of manualised mindfulness-based cognitive therapy (MBCT) alone or as an adjunct treatment to treatment-as-usual (TAU) [31]. MBCT is a psychological group-based intervention that combines intensive training in mindfulness meditation with elements of cognitive behavioural therapy (CBT) for depression. Mindfulness can be operationally defined as non-judgemental awareness of present moment experience [32]. As MBCT was originally designed to prevent relapse rather than to reduce symptoms during a current episode, we will accept studies that introduce minor adaptations to the *delivery* of the manualised approach (e.g. incorporating specific support for managing current depressive symptoms, particularly around the engagement in regular practice, and rephrasing psycho-educational components to address current depression rather than relapse, *without altering the structure or core content of the manual*). Other types of mindfulness-based interventions will not be included. MBCT studies will be considered regardless of delivery format (e.g. physical group setting versus videoconference).

Types of comparators

This may include TAU for depression, including pharmacological and psychological treatments (e.g., CBT) or active controls. The latter will include established interventions as well as control interventions designed to mimic effects of mindfulness-based interventions in some or all aspects with the exception of the core element of mindfulness.

For peer review only - http://bmjopen.bmj.com/site/about/guidelines.xhtml

Participants

We will include studies that recruited adults with current Major Depressive Disorder (MDD) [33, 34] and studies which included mixed groups of patients with residual depressive symptoms or full episodes in the context of a chronic or treatment-resistant course of the disorder. In the latter case, participants with only residual symptoms will be excluded from the primary analysis approach (one-stage meta-analysis). As DTD does not link to an objective way of assessment, we will opt for a maximally inclusive interpretation of the term and include any studies that selected patients based on previous treatment non-response/remission, treatment-resistance or chronicity. We will exclude studies with participants below the age of 18 and studies that have exclusively recruited participants over the age of 65 given that the factors involved in the onset, maintenance and recurrence of depression in these age groups can differ from those in adults within this age bracket [35, 36] .

Outcomes

The main type of outcome will be severity of depressive symptomatology as assessed using standard depression outcome measures. Standard depression outcome measures may include self-report measures and observer-rated measures. If both types of measures are available, we will investigate effects on both types of outcome measures. If several measures are available for a single study, preference will be given to the measure that was used as the primary outcome of the original study. To compare outcomes across studies using different outcome measures, we will transform scores from these measures into standardised scores using the common metric developed by Wahl et al. [37] . Where a measure is not part of the common metric approach, we will transform the measure into one that is part of the common metric using published conversion tables [38] , and then derive the common metric.

For peer review only - http://bmjopen.bmj.com/site/about/guidelines.xhtml

To facilitate clinical interpretation of results, we will derive dichotomous outcomes reflecting remission, improvement and deterioration. To apply a common rule for all studies, common metric scores will be converted to the PHQ-9 and dichotomous outcomes computed based on this measure. Following conventions for the PHQ-9 used in pragmatic trials and health services, remission will be defined as a score of less than 10 [39, 40], i.e. a shift from “caseness” to a non-case score. Definition of clinically meaningful change will follow suggestions from recent trials in treatment-resistant depression, which has indicated a threshold of 6 or more points [41, 42] . We will therefore define clinically meaningful improvement as a reduction of 6 or more points and clinically meaningful deterioration as an increase of 6 or more points on the PHQ-9.

A more detailed analysis of negative effects including suicidality is covered in a separate protocol covering a network meta-analysis of MBCT and another individual psychotherapeutic intervention (Michalak et al., in submission). Secondary outcomes such as mindfulness, self-compassion and quality of life will be collected and may be used for future analysis.

Moderators

We will also investigate potential moderators of treatment response, i.e. factors that determine whether a person responds better to MBCT as compared to the control condition or vice versa. These will include potential moderators on the individual patient level (e.g. sociodemographic characteristics, history of childhood trauma, and clinical characteristics) and on the study level. We will include patient level characteristics in the analyses, if they are reported across a sufficient number of studies and datasets (see statistical analysis section for criteria).

Sociodemographic and clinical characteristics that we aim to investigate include age [43] , gender [44] , marital status [45] , education [46] , employment [47] , childhood

For peer review only - http://bmjopen.bmj.com/site/about/guidelines.xhtml
For peer review only

adversity [48] , age at onset of depression [49] , number of previous episodes [50] , duration of current episode [51] , current suicidality [52] , current antidepressants [53] , past antidepressants and treatment-resistance to antidepressants [54] , co-morbid anxiety disorder, comorbid obsessive-compulsive disorder, co-morbid post-traumatic stress disorder, co-morbid substance abuse or dependence, co-morbid eating disorder, co-morbid personality disorder [43], and level of depressive symptoms at entry [55] . Study level characteristics will include type of delivery (remote versus face-to-face), type of control condition (TAU, active control), type of DTD (non-response/remission, treatment-resistance, chronic depression) and study quality.

Timing of outcome assessments

As follow-up periods of the studies differ, we will group follow-up periods in the following categories: 8 to 10 weeks post-randomisation (post-treatment), 11 to 34 weeks post-randomisation (follow-up 1), and 35 to 60 weeks post-randomisation (follow-up 2).

Searches and study selection

We searched PubMed, Web of Science, Scopus, PsychINFO, EMBASE, and the Cochrane Controlled Trials Register using index and free terms, jointly with Boolean operators, on four tiers, namely: (1) depressive disorder, (2) non-response, treatment resistance and chronicity, (3) mindfulness-based cognitive therapy, and (4) randomised-controlled trial. The search terms used for the different databases are shown in the appendix. We did not search grey literature or trial registries beyond the Cochrane Controlled Trials Register, and we did not consult experts outside of the research team.

We searched for any study published up until the day of the search, on 17 June 2024. There was no lower time limit. Initial screening of titles was performed by two independent reviewers (TB, JM), with the publication abstract being obtained if selected by at least one reviewer. Abstracts were screened by the same two independent reviewers, with

For peer review only - http://bmjopen.bmj.com/site/about/guidelines.xhtml

disagreements about eligibility being referred to a third reviewer whose decision was final. An update search will be conducted immediately before the start of data analysis.

Risk of bias assessment

The validity of the studies will be assessed using the Risk of Bias tool by the Cochrane Collaboration (version 2) [56, 57] . Possible sources of bias assessed by the tool are (1) random sequence generation, (2) allocation concealment, (3) blinding of outcome assessors, (4) incomplete outcome data, (5) selective outcome reporting, and (6) other threats to validity including lack of compliance and different timing of outcome assessments. Data from the published papers of the trials will be evaluated by two independent assessors. Agreement rate will be reported, and any disagreements will be solved through discussion. A sensitivity analysis will be performed excluding studies judged to be at high risk of bias, or where risk of bias is unclear.

Patient and public involvement

A lived experience advisory group, led by MR and consisting of experts who have been involved in previous trials of MBCT, will be consulted for the interpretation of study results and their dissemination. A coproduced lay summary of results will be written for dissemination alongside papers for peer review journals.

IPD data collection and aggregation

All corresponding authors of the articles selected for inclusion were contacted via email by TB with an invitation to participate in the review and to share the IPD from their primary study. The standard letter described all relevant details of the study including its purpose, main research questions, and the variables of interest and ended with a request to share the raw data from the study. Corresponding authors of studies were invited to serve as collaborators for the project. If corresponding authors did not respond within four weeks, we sent a second email request. If this had failed, a second author was contacted. Apart from one

For peer review only - http://bmjopen.bmj.com/site/about/guidelines.xhtml

study, whose authors could not be contacted, none of the selected studies required contact attempts beyond a second author and the responding authors agreed to share their data.

Data checking and integrity

Authors were requested to provide a codebook or data dictionary explaining variable names, coding of values, measurement units etc. After accepting the invitation to collaborate and signing the data transfer agreement, the study authors shared their IPD via secure data transfer portal at the University of Surrey. Data is stored securely on University of Surrey servers in accordance with the European Union's General Data Protection Regulation Act and the UK's Data Protection Act 2018.

TB and MH will take primary responsibility for preparing and extracting outcome data and other characteristics, with MV independently checking extractions for accuracy. Received datasets will be reviewed to assess completeness and checked against the reported data from relevant study publications. The numbers of participants, overall and by arm, will be confirmed to correspond with the numbers in the study publication. Ranges of values will be inspected for clearly erroneous data. Baseline participant demographic data will be inspected by arm, with the aim of checking that the IPD matches the data reported in the publication, in terms of the numbers of participants with reported data, and overall aggregated values for each variable. Discrepancies will be referred to the study authors for investigation and any remaining discrepancies will be recorded. For each included trial, we will extract information on funding sources and any reported conflicts of interest, and this information will be presented alongside other study characteristics.

Although the main search and initial receipt of IPD occurred before the submission of this protocol for publication, the protocol was registered in PROSPERO (CRD42022332039) beforehand. No data analysis or outcome-driven decisions were made prior to finalizing the protocol, and the methods described here were pre-specified prior to data inspection.

For peer review only - http://bmjopen.bmj.com/site/about/guidelines.xhtml

Data harmonization

Once datasets from the individual studies have been checked and outcomes standardised, they will be merged into the final dataset. The final dataset for analysis will be checked again for accuracy by a researcher in the study team.

STATISTICAL ANALYSIS

To finalise the selection of moderator variables, we will determine presence of these variables across the individual data sets and decide on ways to standardise these variables where needed, for example by collapsing categories. Potential moderator variables will be included in the analyses if the variable has at least 40% available data in the final dataset and has been recorded by at least 4 studies.

To investigate the efficacy of MBCT compared to control conditions and test moderating effects of individual patient level and study level characteristics, we will conduct one-stage and two-stage IPD meta-analyses (IPD-MAs). Random-effects models will be used throughout because we expect high statistical heterogeneity due to variation in control groups (treatment as usual, active controls) and study populations (treatment non-responders/remitters, treatment-resistance, chronic depression).

The main research question of the study (whether MBCT is efficacious compared to control conditions) will be addressed via a maximum of three pairwise comparisons: (1) MBCT versus treatment-as-usual controls, (2) MBCT versus active controls, and (3) MBCT versus all controls.

By default, all analyses will be conducted in a Bayesian framework using JAGS (Just Another Gibbs Sampler)/BUGS (Bayesian analysis Using Gibbs Sampling) [58]. We chose a Bayesian approach because it offers greater flexibility in the parametrization of the (one-stage) IPD-MA models, and because this allows for a more intuitive interpretation of results

For peer review only - http://bmjopen.bmj.com/site/about/guidelines.xhtml

based on posterior tail probabilities [59] . Across all analyses, findings will be considered "significant" when the credibility interval of the relevant effect does not include zero.

Two-stage random effects meta-analysis

We will first conduct a conventional meta-analysis using data extracted from the published papers. We will calculate Hedges' g to determine the effect sizes of the difference between the intervention and the control conditions reported in the papers. To investigate heterogeneity of effect sizes, we will calculate I^2, the percentage of variation not attributable to sampling error, as well as 95% prediction intervals (PIs) around the pooled effect [60, 61] . Leave-one-out analyses will be conducted to identify potential outliers and/or influential studies. Small-study effects will be investigated visually using funnel plot inspection and the Egger's test for asymmetry. A weakly informative Half-Cauchy (0,0.5) prior will be used for the between-study heterogeneity variance τ^2 [62], and flat normal priors for all other parameters. A detailed justification for this prior setup has been derived in previous work [63].

One-stage random effects meta-analysis using IPD

One-stage IPD meta-analysis models will be used as the primary analytic approach. To analyse the effects on common metrics-converted symptom severity scores, an identity-link linear mixed effects model with stratified intercepts and trial-specific (heteroscedastic) error terms will be used. Let y_{ik} be the depressive symptom severity of patient i in trial k (with k=1, …, K studies included in the meta-analysis). The one-stage IPD can then be defined as:

$$y_{ik} = \alpha_k + \theta T_{ik} + u_k T_{ik} + \beta_k \left(X_{ik} - \overline{X}_{ik}\right) + \varepsilon_{ik} \tag{1}$$

$$u_k \sim \mathcal{N}\left(0,\tau_\theta^2\right) \qquad \varepsilon_{ik} \sim \mathcal{N}\left(0,\sigma_k^2\right)$$

Where T_{ik} is the treatment indicator, X_{ik} is the symptom severity score at baseline, $\overline{X}_{ik}$ is the mean of X for each specific trial, α_k is the stratified trial intercept, and u_k is the

For peer review only - http://bmjopen.bmj.com/site/about/guidelines.xhtml

trial-specific random slope of the average treatment effect θ. For this model, priors mirroring the two-stage approach will be used throughout, with a uniform distribution assigned to σ_k^2.

The same model specification, but with a logit link, will be used to model the effects on binary outcomes, including remission, clinically meaningful improvement and clinically meaningful deterioration based on PHQ-9 thresholds:

$$\log_e\left(\frac{\pi_{ik}}{1-\pi_{ik}}\right) = \alpha_k + \theta T_{ik} + u_k T_{ik} + \beta_k\left(X_{ik} - \bar{X}_{ik}\right) + \varepsilon_{ik}$$
$$y_{ik} \sim \text{Bern}(\pi_{ik}) \quad u_k \sim \mathcal{N}\left(0,\tau_\theta^2\right) \quad \varepsilon_{ik} \sim \mathcal{N}\left(0,\sigma_k^2\right) \qquad (2)$$

Where y_{ik} now represents the patient's binary outcome of interest. Numbers needed to treat (*NNT*) corresponding to the estimated SMD values will be calculated using the method by Furukawa and Leucht [64], and as the inverse of the absolute risk reduction otherwise.

Moderator analyses

We will explore potential moderators of treatment effect by adding individual participant level and study level treatment-covariate interactions into the main IPD-MA model. To optimise power, these analyses will be done on treatment effects at post-treatment as all studies can provide data for this timepoint.

Missing data

We will record the percentage of individual participant missing data for baseline characteristics and outcomes in each of the studies included in the meta-analyses. All analyses will be conducted according to the "intention-to-treat" principle, so that analyses target a treatment policy estimand [65]. Missing data will be handled using multiple imputation (fully conditional specification; MICE algorithm [66] under the missing at random (MAR) assumption. For the main effectiveness analysis, multilevel two-stage imputation models with heteroscedastic errors will be used to account for the nested data

For peer review only - http://bmjopen.bmj.com/site/about/guidelines.xhtml

structure [67]. Highly collinear variables will be removed as predictors, as well as variables with systematically missing information ("structural zeros"). A total of m=50 imputation sets will be generated. A separate imputation model will be constructed for each focal moderator, including auxiliary variables with a maximum of 10% missingness. Imputations will be generated separately for each of the treatment groups (intervention, control) to account for treatment-covariate interactions. All analysis models will be fitted in the multiply imputed data. Parameter estimates and test statistics will then be aggregated by mixing the draws from the posterior distribution of each completed dataset [68]. Sensitivity analyses will be performed to compare results using observed and imputed data [69] .

Subgroup analyses

We plan to examine the efficacy of MBCT and potential moderators of treatment outcomes in subgroups defined by the conventional descriptive categories used by the individual studies to define their populations, distinguishing between studies that recruited patients characterised as treatment non-responders/remitters, treatment-resistant, or as suffering from chronic depression. These analyses will be based on subsamples created from the final IPD dataset.

Sensitivity analyses

As a sensitivity analysis, we will repeat all main effectiveness analyses using equivalent frequentist (one- and two-stage) approaches. Additionally, we will conduct sensitivity analyses to investigate whether sources of heterogeneity affect the overall effect size estimate and robustness of our findings. This will include exploring the role of risk of bias and other potential study level sources of heterogeneity that may become obvious after data have been examined. These analyses will be conducted using a two-stage IPD-MA model.

DISCUSSION

For peer review only - http://bmjopen.bmj.com/site/about/guidelines.xhtml

For peer review only

This IPD meta-analysis will provide estimates of efficacy for the use of MBCT in DTD and offer comprehensive information about potential moderators of treatment effects. As such it will contribute to arguments in favour of or against implementing MBCT as a treatment for DTD and may help to decide whether MBCT is a recommendable treatment option for this group. This is important given the need to improve the care and management of DTD and the increasing recognition that psychological therapies can have an integral role in this endeavour. Information about potential moderators of treatment effects will help to refine the understanding of who may or may not benefit from MBCT for DTD.

We have chosen the emerging concept of DTD as an umbrella because it provides a broad conceptualisation of depression that remains burdensome despite usual treatment attempts. Most available trials have operationalised this construct using narrower criteria such as non-response, non-remission, treatment-resistance, or chronicity, and our inclusion criteria therefore reflect how DTD has been studied to date. These characteristics represent core manifestations of DTD, and their use ensures consistency and feasibility for the planned meta-analysis. We acknowledge, however, that DTD extends beyond these dimensions, for example in its emphasis on functional outcomes, and generalisability of findings to the broader construct should be interpreted with this in mind.

The chosen approach using IPD has several advantages over conventional meta-analyses including the possibility to assess whether individual participant level characteristics influence treatment effects with more specificity and power. Limitations of the approach include the fact that inconsistencies across studies regarding the format and type of variables reported may restrict our ability to investigate moderators of treatment effects and that transformation of outcome measures to a common metric across all studies may come with a small loss of information. To address this latter point, the current study will complement one-stage IPD analyses with a conventional two-stage approach. A more general concern that is

For peer review only - http://bmjopen.bmj.com/site/about/guidelines.xhtml

often raised for IPD meta-analyses is inclusion bias as it may be difficult to obtain IPD for all selected studies.

This IPD meta-analysis is registered as part of a broader project that examines a wider range of interventions and outcomes, including both beneficial and adverse effects. The present protocol specifies procedures for the comparison between MBCT and control conditions in MBCT trials, as we assume that this comparison is of particular interest given the need to establish interventions for DTD. To our knowledge, this is the first IPD meta-analysis to investigate the effects of MBCT in DTD and assess moderators of treatment effects. As evidence from trials of MBCT for DTD has now accumulated to a point where the use of MBCT for DTD can be considered for endorsement in treatment guidelines, findings from this IPD meta-analysis have the potential to inform treatment recommendations and decisions regarding implementation. The results will provide clinicians, healthcare providers and people who are suffering from DTD with information on the characteristics that may make it more or less likely for patients to benefit from this treatment and thus facilitate implementation.

ETHICS AND DISSEMINATION

No local ethical review was necessary following consultation with the Ethics and Governance Board of the University of Surrey. The investigators of the primary trials have local ethical approval for sharing the data. TB and MH will be responsible for oversight of the database and analyses will be conducted by MH and TB. Patient privacy will be ensured by adhering to University of Surrey guidance on data management and storage. Exchange of data is governed by inter-institutional data sharing agreements. All data were pseudonymised (de-identified) before sharing, shared through secure upload facilities and are stored on secure servers. The data set will not be open access. Researchers of good standing may receive access pending on institutional approvals on data transfer.

For peer review only - http://bmjopen.bmj.com/site/about/guidelines.xhtml

We will disseminate the work in peer-reviewed journals, provide lay summaries and discuss findings with patient and lived experience groups. Other means of communication to disseminate the results will include conference presentations and social media announcements.

CONTRIBUTIONS

TB, MN, JM, MV and MH conceptualised and designed the study. TB developed the search strategy and contacted the primary authors, who contributed to further refinement of the design and approach of the study. TB, MV and MH are responsible for building the database. MH provides statistical expertise and is responsible for data analyses. TB drafted the protocol manuscript with statistical input by MH, which was critically revised by all authors. All authors read and approved the final version. TB serves as the guarantor of the review.

FUNDING STATEMENT

This research received no specific grant from any funding agency in the public, commercial or not-for-profit sectors.

ACKNOWLEDGMENTS

JMM has a ‘Miguel Servet’ research contract from the ISCIII (CP21/00080), and he is grateful to the Spanish CIBER of Epidemiology and Public Health (CBERESP CB22/02/00052< ISCIII) for their support.

COMPETING INTERESTS STATEMENT

TB is the co-author of a book on mindfulness-based interventions and regularly offers workshops on mindfulness-based interventions. He is the co-investigator of a programme grant evaluating an adapted MBCT course for adolescents experiencing depression.

MN is author of a book chapter on mindfulness-based interventions and offers workshops on mindfulness-based interventions.

For peer review only - http://bmjopen.bmj.com/site/about/guidelines.xhtml

JM is the director of the Achtsamkeitsinstitut Ruhr (an institute offering mindfulness training) and principal investigator of several DFG (German Science Foundation) research projects. He receives royalties from mindfulness books he has authored.

MV reports no conflicts of interest.

CC is the co-author of a book on mindfulness-based interventions.

JMGW co-developed MBCT, and was founding Director of the University of Oxford Mindfulness Centre. He receives royalties from books describing the MBCT program. He also receives honoraria from lectures and training workshops on mindfulness.

AC leads a professional practice (Istituto Mente Corpo) that offers courses of mindfulness-based interventions.

SE is the author of book on Mindfulness-Based Cognitive Therapy for the treatment of chronic and treatment-resistant depression.

KD declares no conflicts of interest.

ZS received book royalties from Guilford Press, workshop fees from the Centre for Mindfulness studies, and revenue from online sales at Mindful Noggin Inc., which are all related to his work as a co-founder of Mindfulness-Based Cognitive Therapy (MBCT).

MCM declares no conflicts of interest.

AS is the founder and director of the Radboudumc Expertise Centre for Mindfulness and co-investigator of a number of studies examining the effectiveness and implementation of mindfulness-based interventions for patients with psychological or somatic disorders and health care professionals.

AAF declares no conflicts of interest.

MGT declares no conflicts of interest.

JMM is associated with the University of Oxford Mindfulness Research Centre.

For peer review only - http://bmjopen.bmj.com/site/about/guidelines.xhtml

BDD is the lead of the University of Exeter AccEPT clinic, which offers courses of MBCT.

CS is a member of a training organisation that has been commissioned by NHS England to deliver MBCT training across NHS Talking Therapies services in England and co-leads the Sussex Mindfulness Centre. CS is a co-investigator of a programme grant evaluating an adapted MBCT course for adolescents experiencing depression and co-investigator on a grant evaluating an adapted MBCT course for NHS staff.

FAR regularly provides workshops on mindfulness-based interventions.

MR declares no conflicts of interest.

MH serves as a statistical consultant for HelloBetter/GetOn Institut für Gesundheitstrainings GmbH, a company that implements digital mental health therapeutics in routine care.

REFERENCES

1. Collaborators G 2019 MD. Global, regional, and national burden of 12 mental disorders in 204 countries and territories, 1990–2019: a systematic analysis for the Global Burden of Disease Study 2019. Lancet Psychiatry. 2022;9:137–50. https://doi.org/10.1016/s2215-0366(21)00395-3.

2. McIntyre RS, Alsuwaidan M, Baune BT, Berk M, Demyttenaere K, Goldberg JF, et al. Treatment-resistant depression: definition, prevalence, detection, management, and investigational interventions. World Psychiatry. 2023;22:394–412. https://doi.org/10.1002/wps.21120.

3. Rush AJ, Sackeim HA, Conway CR, Bunker MT, Hollon SD, Demyttenaere K, et al. Clinical research challenges posed by difficult-to-treat depression. Psychol Med. 2022;52:419–32. https://doi.org/10.1017/s0033291721004943.

4. McAllister-Williams RH, Arango C, Blier P, Demyttenaere K, Falkai P, Gorwood P, et al. The identification, assessment and management of difficult-to-treat depression: An international consensus statement. J Affect Disord. 2020;267:264–82. https://doi.org/10.1016/j.jad.2020.02.023.

For peer review only - http://bmjopen.bmj.com/site/about/guidelines.xhtml

5. Jaffe DH, Rive B, Denee TR. The humanistic and economic burden of treatment-resistant depression in Europe: a cross-sectional study. BMC Psychiatry. 2019;19:247. https://doi.org/10.1186/s12888-019-2222-4.

6. Bhattacharya R, Shen C, Sambamoorthi U. Excess risk of chronic physical conditions associated with depression and anxiety. BMC Psychiatry. 2014;14. https://doi.org/10.1186/1471-244x-14-10.

7. Moeller SB, Gbyl K, Hjorthøj C, Andreasen M, Austin SF, Buchholtz PE, et al. Treatment of difficult-to-treat depression – clinical guideline for selected interventions. Nord J Psychiatry. 2022;76:177–88. https://doi.org/10.1080/08039488.2021.1952303.

8. Markowitz JC, Wright JH, Peeters F, Thase ME, Kocsis JH, Sudak DM. The Neglected Role of Psychotherapy for Treatment-Resistant Depression. Am J Psychiatry. 2022;179:90–3. https://doi.org/10.1176/appi.ajp.2021.21050535.

9. McHugh RK, Whitton SW, Peckham AD, Welge JA, Otto MW. Patient Preference for Psychological vs Pharmacologic Treatment of Psychiatric Disorders: A Meta-Analytic Review. J Clin Psychiatry. 2013;74:595–602. https://doi.org/10.4088/jcp.12r07757.

10. Segal ZV, Williams JMG, Teasdale J. Mindfulness-Based Cognitive Therapy for Depression. 2013.

11. Barnhofer T, Dunn BD, Strauss C, Ruths FA, Barrett B, Ryan M, et al. Mindfulness-based cognitive therapy versus treatment as usual after non-remission with NHS Talking Therapies high-intensity psychological therapy for depression: a UK-based clinical effectiveness and cost-effectiveness randomised, controlled, superiority trial. Lancet Psychiatry. 2025;12:433–46. https://doi.org/10.1016/s2215-0366(25)00105-1.

12. Kuyken W, Warren FC, Taylor RS, Whalley B, Crane C, Bondolfi G, et al. Efficacy of Mindfulness-Based Cognitive Therapy in Prevention of Depressive Relapse: An Individual Patient Data Meta-analysis From Randomized Trials. Jama Psychiat. 2016;73:565. https://doi.org/10.1001/jamapsychiatry.2016.0076.

13. Ma SH, Teasdale JD. Mindfulness-Based Cognitive Therapy for Depression: Replication and Exploration of Differential Relapse Prevention Effects. J Consult Clin Psych. 2004;72:31–40. https://doi.org/10.1037/0022-006x.72.1.31.

14. Teasdale JD, Segal ZV, Williams JMG, Ridgeway VA, Soulsby JM, Lau MA. Prevention of relapse/recurrence in major depression by mindfulness-based cognitive therapy. J Consult Clin Psych. 2000;68:615–23. https://doi.org/10.1037/0022-006x.68.4.615.

15. Creswell JD, Lindsay EK. How Does Mindfulness Training Affect Health? A Mindfulness Stress Buffering Account. Current Directions in Psychological Science. 2014;23. https://doi.org/10.1177/0963721414547415.

16. Lindsay EK, Creswell DJ. Mechanisms of mindfulness training: Monitor and Acceptance Theory (MAT). Clinical Psychology Review. 2017;51. https://doi.org/10.1016/j.cpr.2016.10.011.

For peer review only - http://bmjopen.bmj.com/site/about/guidelines.xhtml

17. Eisendrath SJ, Delucchi K, Bitner R, Fenimore P, Smit M, McLane M. Mindfulness-Based Cognitive Therapy for Treatment-Resistant Depression: A Pilot Study. Psychother Psychosom. 2008;77:319–20. https://doi.org/10.1159/000142525.

18. Barnhofer T, Crane C, Hargus E, Amarasinghe M, Winder R, Williams JMG. Mindfulness-based cognitive therapy as a treatment for chronic depression: A preliminary study. Behav Res Ther. 2009;47:366–73. https://doi.org/10.1016/j.brat.2009.01.019.

19. Eisendrath SJ, Gillung E, Delucchi KL, Segal ZV, Nelson JC, McInnes LA, et al. A Randomized Controlled Trial of Mindfulness-Based Cognitive Therapy for Treatment-Resistant Depression. Psychother Psychosom. 2016;85:99–110. https://doi.org/10.1159/000442260.

20. Cladder-Micus MB, Speckens AEM, Vrijsen JN, Donders ART, Becker ES, Spijker J. Mindfulness-based cognitive therapy for patients with chronic, treatment-resistant depression: A pragmatic randomized controlled trial. Depress Anxiety. 2018;35:914–24. https://doi.org/10.1002/da.22788.

21. Michalak J, Schultze M, Heidenreich T, Schramm E. A randomized controlled trial on the efficacy of mindfulness-based cognitive therapy and a group version of cognitive behavioral analysis system of psychotherapy for chronically depressed patients. J Consult Clin Psych. 2015;83:951–63. https://doi.org/10.1037/ccp0000042.

22. Chiesa A, Castagner V, Andrisano C, Serretti A, Mandelli L, Porcelli S, et al. Mindfulness-based cognitive therapy vs. psycho-education for patients with major depression who did not achieve remission following antidepressant treatment. Psychiat Res. 2015;226:474–83. https://doi.org/10.1016/j.psychres.2015.02.003.

23. Garcia A, Yáñez AM, Bennasar-Veny M, Navarro C, Salva J, Ibarra O, et al. Efficacy of an adjuvant non-face-to-face multimodal lifestyle modification program for patients with treatment-resistant major depression: A randomized controlled trial. Psychiatry Res. 2023;319:114975. https://doi.org/10.1016/j.psychres.2022.114975.

24. Foroughi A, Sadeghi K, Parvizifard A, Moghadam AP, Davarinejad O, Farnia V, et al. The effectiveness of mindfulness-based cognitive therapy for reducing rumination and improving mindfulness and self-compassion in patients with treatment-resistant depression. Trends Psychiatry Psychotherapy. 2020;42:138–46. https://doi.org/10.1590/2237-6089-2019-0016.

25. Mathew T, Nordström K. Comparison of One-Step and Two-Step Meta-Analysis Models Using Individual Patient Data. Biom J. 2010;52:271–87. https://doi.org/10.1002/bimj.200900143.

26. Clarke MJ. Individual patient data meta-analyses. Best Pr Res Clin Obstet Gynaecol. 2005;19:47–55. https://doi.org/10.1016/j.bpobgyn.2004.10.011.

27. Riley RD, Lambert PC, Abo-Zaid G. Meta-analysis of individual participant data: rationale, conduct, and reporting. BMJ. 2010;340 feb05 1:c221. https://doi.org/10.1136/bmj.c221.

For peer review only

28. Jones AP, Riley RD, Williamson PR, Whitehead A. Meta-analysis of individual patient data versus aggregate data from longitudinal clinical trials. Clin Trials. 2009;6:16–27. https://doi.org/10.1177/1740774508100984.

29. Cooper H, Patall EA. The Relative Benefits of Meta-Analysis Conducted With Individual Participant Data Versus Aggregated Data. Psychol Methods. 2009;14:165–76. https://doi.org/10.1037/a0015565.

30. Stewart LA, Clarke M, Rovers M, Riley RD, Simmonds M, Stewart G, et al. Preferred Reporting Items for a Systematic Review and Meta-analysis of Individual Participant Data: The PRISMA-IPD Statement. Jama. 2015;313:1657–65. https://doi.org/10.1001/jama.2015.3656.

31. Löfholm CA, Brännström L, Olsson M, Hansson K. Treatment-as-usual in effectiveness studies: What is it and does it matter? Int J Soc Welf. 2013;22:25–34. https://doi.org/10.1111/j.1468-2397.2012.00870.x.

32. Kabat-Zinn J. Mindfulness-Based Interventions in Context: Past, Present, and Future. Clinical Psychology: Science and Practice. 2003;10:144–56. https://doi.org/10.1093/clipsy.bpg016.

33. Association AP. Diagnostic and statistical manual of mental disorders (5th ed.). Arlington, VA: Author; 2013.

34. Association AP. Diagnostic and statistical manual of mental disorders (4th ed.). Washington, DC: Author; 2000.

35. Korten NCM, Comijs HC, Lamers F, Penninx BWJH. Early and late onset depression in young and middle aged adults: Differential symptomatology, characteristics and risk factors? J Affect Disord. 2012;138:259–67. https://doi.org/10.1016/j.jad.2012.01.042.

36. Devanand DP, Adorno E, Cheng J, Burt T, Pelton GH, Roose SP, et al. Late onset dysthymic disorder and major depression differ from early onset dysthymic disorder and major depression in elderly outpatients. J Affect Disord. 2004;78:259–67. https://doi.org/10.1016/s0165-0327(02)00307-5.

37. Wahl I, Löwe B, Bjorner JB, Fischer F, Langs G, Voderholzer U, et al. Standardization of depression measurement: a common metric was developed for 11 self-report depression measures. J Clin Epidemiology. 2014;67:73–86. https://doi.org/10.1016/j.jclinepi.2013.04.019.

38. Rush AJ, Trivedi MH, Ibrahim HM, Carmody TJ, Arnow B, Klein DN, et al. The 16-Item quick inventory of depressive symptomatology (QIDS), clinician rating (QIDS-C), and self-report (QIDS-SR): a psychometric evaluation in patients with chronic major depression. Biol Psychiatry. 2003;54:573–83. https://doi.org/10.1016/s0006-3223(02)01866-8.

39. Kroenke K, Spitzer RL, Williams JBW. The PHQ-9: Validity of a brief depression severity measure. J Gen Intern Med. 2001;16:606–13. https://doi.org/10.1046/j.1525-1497.2001.016009606.x.

For peer review only - http://bmjopen.bmj.com/site/about/guidelines.xhtml

40. Löwe B, Unützer J, Callahan CM, Perkins AJ, Kroenke K. Monitoring Depression Treatment Outcomes With the Patient Health Questionnaire-9. Med Care. 2004;42:1194–201. https://doi.org/10.1097/00005650-200412000-00006.

41. Hudgens S, Floden L, Blackowicz M, Jamieson C, Popova V, Fedgchin M, et al. Meaningful Change in Depression Symptoms Assessed with the Patient Health Questionnaire (PHQ-9) and Montgomery-Åsberg Depression Rating Scale (MADRS) Among Patients with Treatment Resistant Depression in Two, Randomized, Double-blind, Active-controlled Trials of Esketamine Nasal Spray Combined With a New Oral Antidepressant. J Affect Disord. 2021;281:767–75. https://doi.org/10.1016/j.jad.2020.11.066.

42. Turkoz I, Alphs L, Singh J, Jamieson C, Daly E, Shawi M, et al. Clinically meaningful changes on depressive symptom measures and patient-reported outcomes in patients with treatment-resistant depression. Acta Psychiatr Scand. 2021;143:253–63. https://doi.org/10.1111/acps.13260.

43. Hölzel L, Härter M, Reese C, Kriston L. Risk factors for chronic depression — A systematic review. J Affect Disorders. 2011;129:1–13. https://doi.org/10.1016/j.jad.2010.03.025.

44. Seedat S, Scott KM, Angermeyer MC, Berglund P, Bromet EJ, Brugha TS, et al. Cross-National Associations Between Gender and Mental Disorders in the World Health Organization World Mental Health Surveys. Arch Gen Psychiatry. 2009;66:785–95. https://doi.org/10.1001/archgenpsychiatry.2009.36.

45. Buckman JEJ, Saunders R, Stott J, Arundell L-L, O'Driscoll C, Davies MR, et al. Role of age, gender and marital status in prognosis for adults with depression: An individual patient data meta-analysis. Epidemiology Psychiatr Sci. 2021;30:e42. https://doi.org/10.1017/s2045796021000342.

46. Trivedi MH, Rush AJ, Wisniewski SR, Nierenberg AA, Warden D, Ritz L, et al. Evaluation of Outcomes With Citalopram for Depression Using Measurement-Based Care in STARD: Implications for Clinical Practice. Am J Psychiatry. 2006;163:28–40. https://doi.org/10.1176/appi.ajp.163.1.28.

47. Oosten AJ van, Mens K van, Blonk RWB, Burdorf A, Tiemens B. The relationship between having a job and the outcome of brief therapy in patients with common mental disorders. BMC Psychiatry. 2023;23:910. https://doi.org/10.1186/s12888-023-05418-z.

48. Nanni V, Uher R, Danese A. Childhood Maltreatment Predicts Unfavorable Course of Illness and Treatment Outcome in Depression: A Meta-Analysis. Am J Psychiat. 2012;169:141–51. https://doi.org/10.1176/appi.ajp.2011.11020335.

49. Klein DN, Schatzberg AF, McCullough JP, Dowling F, Goodman D, Howland RH, et al. Age of onset in chronic major depression: relation to demographic and clinical variables, family history, and treatment response. J Affect Disord. 1999;55:149–57. https://doi.org/10.1016/s0165-0327(99)00020-8.

50. Bromberger JT, Schott L, Kravitz HM, Joffe H. Risk factors for major depression during midlife among a community sample of women with and without prior major depression: are

For peer review only

they the same or different? Psychol Med. 2015;45:1653–64. https://doi.org/10.1017/s0033291714002773.

51. Myklebost SB, Gjestad R, Inal Y, Hammar Å, Nordgreen T. Predictors of Treatment Response to an Internet-Delivered Intervention Targeting Residual Cognitive Symptoms After Major Depressive Disorder. Front Psychiatry. 2022;13:795698. https://doi.org/10.3389/fpsyt.2022.795698.

52. Courtney DB, Watson P, Krause KR, Chan BWC, Bennett K, Gunlicks-Stoessel M, et al. Predictors, Moderators, and Mediators Associated With Treatment Outcome in Randomized Clinical Trials Among Adolescents With Depression. JAMA Netw Open. 2022;5:e2146331. https://doi.org/10.1001/jamanetworkopen.2021.46331.

53. Cuijpers P, Sijbrandij M, Koole SL, Andersson G, Beekman AT, Reynolds CF. Adding psychotherapy to antidepressant medication in depression and anxiety disorders: a meta-analysis. World Psychiatry. 2014;13:56–67. https://doi.org/10.1002/wps.20089.

54. DeRubeis RJ, Hollon SD, Amsterdam JD, Shelton RC, Young PR, Salomon RM, et al. Cognitive Therapy vs Medications in the Treatment of Moderate to Severe Depression. Arch Gen Psychiatry. 2005;62:409–16. https://doi.org/10.1001/archpsyc.62.4.409.

55. Button KS, Wiles NJ, Lewis G, Peters TJ, Kessler D. Factors associated with differential response to online cognitive behavioural therapy. Soc Psychiatry Psychiatr Epidemiology. 2012;47:827–33. https://doi.org/10.1007/s00127-011-0389-1.

56. Furlan AD, Pennick V, Bombardier C, Tulder M van, Group EB Cochrane Back Review. 2009 Updated Method Guidelines for Systematic Reviews in the Cochrane Back Review Group. Spine. 2009;34:1929–41. https://doi.org/10.1097/brs.0b013e3181b1c99f.

57. Sterne JAC, Savović J, Page MJ, Elbers RG, Blencowe NS, Boutron I, et al. RoB 2: a revised tool for assessing risk of bias in randomised trials. Bmj. 2019;366:l4898. https://doi.org/10.1136/bmj.l4898.

58. Lunn D, Jackson C, Best N, Thomas A, Spiegelhalter D. The BUGS Book, A Practical Introduction to Bayesian Analysis. 2019. https://doi.org/10.1201/b13613.

59. Goligher EC, Heath A, Harhay MO. Bayesian statistics for clinical research. Lancet. 2024;404:1067–76. https://doi.org/10.1016/s0140-6736(24)01295-9.

60. Higgins JPT, Thompson SG, Deeks JJ, Altman DG. Measuring inconsistency in meta-analyses. BMJ. 2003;327:557–60. https://doi.org/10.1136/bmj.327.7414.557.

61. Higgins JPT, Thompson SG. Quantifying heterogeneity in a meta-analysis. Stat Med. 2002;21:1539–58. https://doi.org/10.1002/sim.1186.

62. Harrer M, Cuijpers P, Furukawa TA, Ebert DD. Doing Meta-Analysis with R, A Hands-On Guide. 2022. https://doi.org/10.1201/9781003107347.

63. Harrer M. Heterogeneity Prior Justification. 2022. https://osf.io/3tfm6. Accessed 16 May 2025.

For peer review only

For peer review only - http://bmjopen.bmj.com/site/about/guidelines.xhtml

64. Furukawa TA, Leucht S. How to Obtain NNT from Cohen's d: Comparison of Two Methods. PLoS ONE. 2011;6:e19070. https://doi.org/10.1371/journal.pone.0019070.

65. Han S, Zhou X. Defining estimands in clinical trials: A unified procedure. Stat Med. 2023;42:1869–87. https://doi.org/10.1002/sim.9702.

66. Buuren van, Groothuis-Oudshoorn K. mice: multivariate imputation by chained equations in R. Journal of Statistical Software. 2011;45:1–67.

67. Resche-Rigon M, White IR. Multiple imputation by chained equations for systematically and sporadically missing multilevel data. Stat Methods Méd Res. 2018;27:1634–49. https://doi.org/10.1177/0962280216666564.

68. Zhou X, Reiter JP. A Note on Bayesian Inference After Multiple Imputation. Am Stat. 2010;64:159–63. https://doi.org/10.1198/tast.2010.09109.

69. Burgess S, White IR, Resche-Rigon M, Wood AM. Combining multiple imputation and meta-analysis with individual participant data. Stat Med. 2013;32:4499–514. https://doi.org/10.1002/sim.5844.

For peer review only

APPENDIX

Search Terms

Database	Search Term
Web of Science	(“MDE” OR “major depressive episode” OR “MDD” OR “major depressive disorder” OR “major depression” OR “depression” OR “dysthymia” OR “dysthymic”) AND (“chronic” OR “persistent” OR “treatment-resistant” OR “non-responders” OR “non-response” OR “non-remitted” OR “dysthymia” OR “dysthymic”) AND (“MBCT” OR "mindfulness-based cognitive therapy") AND (“randomized controlled trial” OR “randomised controlled trial” OR “randomized-controlled trial” OR “randomised-controlled trial” OR “RCT” OR “randomized” or “randomised” OR “randomly allocated” OR “randomly assigned”)
Scopus	(“MDE” OR “major depressive episode” OR “MDD” OR “major depressive disorder” OR “major depression” OR “depression” OR “dysthymia” OR “dysthymic”) AND (“chronic” OR “persistent” OR “treatment-resistant” OR “non-responders” OR “non-response” OR “non-remitted” OR “dysthymia” OR “dysthymic”) AND (“MBCT” OR "mindfulness-based cognitive therapy") AND (“randomized controlled trial” OR “randomised controlled trial” OR “randomized-controlled trial” OR “randomised-controlled trial” OR “RCT” OR “randomized” or “randomised” OR “randomly allocated” OR “randomly assigned”)
PubMed	(“MDE” OR “major depressive episode” OR “MDD” OR “major depressive disorder” OR “major depression” OR “depression” OR “dysthymia” OR “dysthymic”) AND (“chronic” OR “persistent” OR “treatment-resistant” OR “non-responders” OR “non-response” OR “non-remitted” OR “dysthymia” OR “dysthymic”) AND (“MBCT” OR "mindfulness-based cognitive therapy") AND (“randomized controlled trial” OR “randomised controlled trial” OR “randomized-controlled trial” OR “randomised-controlled trial” OR “RCT” OR “randomized” or “randomised” OR “randomly allocated” OR “randomly assigned”)
PsycInfo	(“MDE” OR “major depressive episode” OR “MDD” OR “major depressive disorder” OR “major depression” OR “depression” OR “dysthymia” OR “dysthymic”) **AND** (“chronic” OR “persistent” OR “treatment-resistant” OR “non-responders” OR “non-response” OR “non-remitted” OR “dysthymia” OR “dysthymic”) **AND** (“MBCT” OR "mindfulness-based cognitive therapy") **AND** (“randomized controlled trial” OR “randomised controlled trial” OR “randomized-controlled trial” OR “randomised-controlled trial” OR “RCT” OR “randomized” or “randomised” OR “randomly allocated” OR “randomly assigned”)
Embase	(‘MDE’ OR ‘major depressive episode’ OR ‘MDD’ OR ‘major depressive disorder’ OR ‘major depression’ OR ‘depression’ OR ‘dysthymia’ OR ‘dysthymic’) **AND** (‘chronic’ OR ‘persistent’ OR ‘treatment-resistant’ OR ‘non-responders’ OR ‘non-response’ OR ‘non-remitted’ OR ‘dysthymia’ OR ‘dysthymic’) **AND** (‘MBCT’ OR ‘mindfulness-based cognitive therapy’) **AND** (‘randomized controlled trial’ OR ‘randomised controlled trial’ OR ‘randomized-controlled trial’ OR ‘randomised-controlled trial’ OR ‘RCT’ OR ‘randomized’ or ‘randomised’ OR ‘randomly allocated’ OR ‘randomly assigned’)

For peer review only - http://bmjopen.bmj.com/site/about/guidelines.xhtml

Cochrane Controlled Trials	(“MDE” OR “major depressive episode” OR “MDD” OR “major depressive disorder” OR “major depression” OR “depression” OR “dysthymia” OR “dysthymic”) AND (“chronic” OR “persistent” OR “treatment-resistant” OR “non-responders” OR “non-response” OR “non-remitted” OR “dysthymia” OR “dysthymic”) AND (“MBCT” OR "mindfulness-based cognitive therapy") AND (“randomized controlled trial” OR “randomised controlled trial” OR “randomized-controlled trial” OR “randomised-controlled trial” OR “RCT” OR “randomized” or “randomised” OR “randomly allocated” OR “randomly assigned”)

For peer review only

For peer review only - http://bmjopen.bmj.com/site/about/guidelines.xhtml

BMJ Open

Efficacy and Moderators of Mindfulness-Based Cognitive Therapy (MBCT) in 'Difficult to Treat' Depression: Protocol for a Systematic Review and Individual Patient Data Meta-Analysis of Randomised Controlled Trials

Journal:	*BMJ Open*
Manuscript ID	bmjopen-2025-106350.R3
Article Type:	Protocol
Date Submitted by the Author:	12-Nov-2025
Complete List of Authors:	Barnhofer, Thorsten; University of Surrey,<br>Niemi, Maria; Karolinska Institutet, Department of Global Public Health<br>Michalak, Johannes; Witten/Herdecke University, Department of Psychology and Psychotherapy<br>Velana, Maria; Witten/Herdecke University, Department of Psychology and Psychotherapy<br>Williams, J Mark; University of Oxford, Department of Psychiatry<br>Chiesa, Alberto; Università di Bologna<br>Eisendrath, Stuart; University of California at San Francisco<br>Delucchi, Kevin; University of California at San Francisco<br>Segal, Zindel; University of Toronto - Scarborough, Graduate Department of Psychological Clinical Science<br>Cladder-Micus, Mira; Radboud University Nijmegen<br>Speckens, Anne; Radboudumc Instituut voor Wetenschappelijk Onderwijs en Opleidingen, Psychiatry<br>Foroughi, Ali Akbar; Kermanshah University of Medical Sciences (KUMS), Department of Clinical Psychology<br>García-Toro, M; Research Network on Chronicity Primary Care and Health Promotion; University of the Balearic Islands; Health Research Institute of the Balearic Islands; University of the Balearic Islands, Department of Medicine<br>Montero-Marin, Jesus; Research & Innovation Unit Parc Sanitari Sant Joan de Déu; University of Oxford, Department of Psychiatry; Consortium for Biomedical Research in Epidemiology and Public Health<br>dunn, barney; University of Exeter<br>Strauss, Clara; Sussex Partnership NHS Foundation Trust; University of Sussex<br>Ruths, Florian; South London and Maudsley NHS Foundation Trust<br>Ryan, Mary; Royal College of Psychiatrists<br>Harrer, Mathias; Technical University of Munich, Department of Psychiatry and Psychotherapy; World Health Organization, Department of Clinical, Neuro and Developmental Psychology; Vrije Universiteit
**Primary Subject Heading**:	Mental health
Secondary Subject Heading:	Mental health

Keywords:	Meta-Analysis, Depression & mood disorders < PSYCHIATRY, Mindfulness

SCHOLARONE™
Manuscripts

Title

Efficacy and Moderators of Mindfulness-Based Cognitive Therapy (MBCT) in 'Difficult to Treat' Depression: Protocol for a Systematic Review and Individual Patient Data Meta-Analysis of Randomised Controlled Trials

Running Title

MBCT in 'Difficult to Treat' Depression

Authors and Affiliations

Thorsten Barnhofer [1], Maria Niemi [2, *], Johannes Michalak [3], Maria Velana [3], J. Mark G. Williams [4], Alberto Chiesa [5], Stuart Eisendrath [6], Kevin Delucchi [6], Zindel V. Segal [7], Mira Cladder-Micus [8], Anne Speckens [9], Ali Akbar Foroughi [10], Mauro Garcia-Toro [11, 12, 13, 14], Jesus Montero-Marin [15, 4, 16], Barnaby D. Dunn [17], Clara Strauss [18, 19], Florian A. Ruths [20], Mary Ryan [21], Mathias Harrer [22, 23]

[1] School of Psychology, University of Surrey, Guildford, UK, t.barnhofer@surrey.ac.uk

[2] Department of Global Public Health, Karolinska Institutet, Stockholm, Sweden, maria.niemi@ki.se

[3] Department of Psychology and Psychotherapy, Witten/Herdecke University, Witten, Germany, Maria.Velana@uni-wh.de , Johannes.Michalak@uni-wh.de

[4] Department of Psychiatry, University of Oxford, Oxford, UK, catherine.crane@psych.ox.ac.uk, mark.williams@psych.ox.ac.uk, jesus.monteromarin@psych.ox.ac.uk

[5] Institute of Psychiatry, Università di Bologna, Bologna, Italy, chiesa@scuola-spc.it

[6] Weill Institute for Neurosciences, University of California at San Francisco, San Francisco, USA, stuart.eisendrath@ucsf.edu, kevin.delucchi@ucsf.edu

For peer review only - http://bmjopen.bmj.com/site/about/guidelines.xhtml

[7] Graduate Department of Psychological Clinical Science, University of Toronto - Scarborough, Toronto, Canada, zindel.segal@utoronto.ca

[8] Behavioural Science Institute, Radboud University Nijmegen, Nijmegen, The Netherlands, Mira.Cladder-Micus@radboudumc.nl

[9] Department of Psychiatry, Radboudumc, Nijmegen, The Netherlands, anne.speckens@radboudumc.nl

[10] Department of Clinical Psychology, Kermanshah University of Medical Sciences (KUMS), Hermanshah, Iran, foroughi_2002@yahoo.com

[11] Research Network on Chronicity, Primary Care and Health Promotion (RICAPPS), Carlos III Health Institute, Madrid, Spain

[12] University Institute of Health Science Research (IUNICS), University of the Balearic Islands, Spain, mauro.garcia@uib.es

[13] Health Research Institute of the Balearic Islands (IdISBa), Palma, Spain

[14] Department of Medicine, University of the Balearic Islands, Palma, Spain

[15] Research & Innovation Unit, Parc Sanitari Sant Joan de Déu, Sant Boi de Llobregat, Barcelona, Spain

[16] Consortium for Biomedical Research in Epidemiology and Public Health (CIBER Epidemiology and Public Health-CIBERESP), Madrid, Spain

[17] Mood Disorders Centre, University of Exeter, Exeter, UK, b.d.dunn@exeter.ac.uk

[18] Sussex Partnership NHS Foundation Trust, Hove, UK, clara.strauss@nhs.net

[19] Sussex Mental Health Research Centre, University of Sussex, Brighton, UK

[20] South London and Maudsley NHS Foundation Trust, London, UK, Florian.Ruths@slam.nhs.uk

[21] Royal College of Psychiatrists, London, UK, mary@highbag.co.uk

For peer review only - http://bmjopen.bmj.com/site/about/guidelines.xhtml

[22] Department of Psychiatry and Psychotherapy, School of Medicine and Health, Technical University of Munich, Munich, Germany

[23] Department of Clinical, Neuro and Developmental Psychology, World Health Organization Collaborating Center for Research and Dissemination of Psychological Interventions, Amsterdam, Public Health Research Institute, Vrije Universiteit, Amsterdam, The Netherlands, mathias.harrer@tum.de

* Corresponding author:

Associate Professor Maria Niemi, Department of Global Public Health, Karolinska Institutet, Stockholm, Sweden, maria.niemi@ki.se

For peer review only

Word Count

Abstract: 300

Main text: 4110

Keywords

Major Depression, Difficult-to-Treat, Mindfulness-Based Cognitive Therapy, Independent Patient Data, Meta-Analysis

For peer review only - http://bmjopen.bmj.com/site/about/guidelines.xhtml

ABSTRACT

Introduction: About 30% of depressed patients suffer from a protracted course in which the disorder continues to cause significant burden despite treatment efforts. While originally developed for relapse prevention, Mindfulness-Based Cognitive Therapy (MBCT) has increasingly been investigated in depressed patients with such 'difficult-to-treat' courses. This is a protocol for an individual participant data (IPD) meta-analysis aiming to determine efficacy and potential moderators of MBCT treatment effects in this group based on evidence from randomised controlled trials.

Methods and analysis: Systematic searches in PubMed, Web of Science, Scopus, PsycINFO, EMBASE, and the Cochrane Controlled Trials Register for randomised controlled trials were completed on 17 June 2024. Authors of identified studies have contributed IPD and data extractions have been completed. An update search will be conducted immediately before the start of data analyses. We will investigate the following outcomes: (a) self-reported and observer-reported severity of depression symptomatology, (b) remission, (c) clinically meaningful improvement and deterioration. One-stage and two-stage IPD meta-analyses will be conducted with one-stage models using the observed IPD from all studies simultaneously as the primary approach. One-stage IPD models will include stratified study intercepts and error terms as well as random effects to capture between-study heterogeneity. Moderator analyses will test treatment-covariate interactions for both individual patient level and study level characteristics.

Ethics and dissemination: The results will inform understanding of the use of MBCT in patients with current 'difficult-to-treat' depression and will contribute to arguments in favour of or against implementing MBCT as a treatment for this group. They will be published in a peer reviewed journal and made available to stakeholders in accessible formats. No local ethical review was necessary following consultation with the Ethics and Governance Board

For peer review only - http://bmjopen.bmj.com/site/about/guidelines.xhtml

of the University of Surrey. Guidance on patient data storage and management will be adhered to throughout.

PROSPERO registration number: CRD42022332039

For peer review only

For peer review only - http://bmjopen.bmj.com/site/about/guidelines.xhtml

STRENGTHS AND LIMITATIONS OF THIS STUDY

- The chosen approach using IPD has several advantages over conventional meta-analyses including the possibility to assess whether individual participant level characteristics influence treatment effects with more specificity and power
- Limitations of the approach include the fact that inconsistencies across studies regarding the format and type of variables reported may restrict our ability to investigate moderators of treatment effects
- Transformation of outcome measures to a common metric across all studies may come with a small loss of information.

For peer review only

For peer review only - http://bmjopen.bmj.com/site/about/guidelines.xhtml

INTRODUCTION

Major Depressive Disorder (MDD) is highly prevalent, debilitating [1] , and in about 30% of patients takes a recurrent or chronic course in which established treatments fail to bring sustained remission [2] . Treatment resistance has traditionally been defined in narrow operational terms (e.g. non-response after two adequate pharmacological treatments), but such definitions vary across studies and exclude many patients with persistent, impairing depression. The broader construct of difficult-to-treat depression (DTD) was recently introduced to capture the true spectrum within a single heuristic, providing a clinically meaningful framework that better reflects the overlap between these groups and the challenges faced in practice [3, 4]. Defined as "depression that continues to cause significant burden despite usual treatment efforts", such courses are associated with continuing functional impairment, reduced quality of life, and significantly increased risk for chronic physical and neurodegenerative disorders [5, 6] . Finding effective treatments for DTD remains an important challenge for depression research [7] .

While treatment approaches for DTD have traditionally been dominated by pharmacological strategies [8] , it is now increasingly acknowledged that effective management requires a broader emphasis on working with symptoms, maximising function and minimizing burden [4] . Psychological interventions have an important contribution to make in this context [9] and emerging evidence suggests that Mindfulness-Based Cognitive Therapy (MBCT [10]), an eight-week treatment that combines elements of cognitive therapy with mindfulness meditation, may be suited for this purpose [11] . Using a mental training approach, the intervention aims to provide patients with portable skills that enable them to disengage from habitual maladaptive responses and respond more adaptively to negative mood and stress.

For peer review only - http://bmjopen.bmj.com/site/about/guidelines.xhtml

MBCT was originally introduced for the prevention of relapse in patients who are currently in remission and has been proven to be effective for this purpose [12] , particularly in patients with highly recurrent courses (a use that is endorsed by treatment guidelines internationally). Early trials restricted the intervention to patients who were currently in full remission as it was assumed that intensive mindfulness practice may be too demanding for those who are suffering from current symptoms [13, 14] . However, as patients who are at high risk of relapse are likely to show residual symptoms between episodes, this restriction was later loosened, and analyses across studies for relapse prevention show that treatment effects increase, rather than decrease, with level of residual symptoms [12] . From a theoretical perspective, this observation is in line with the view that mindfulness skills serve to buffer the effects of negative mood and stress [15, 16] , which implies that effects should become more visible under conditions of higher symptoms or stress.

Based on this reasoning, research has extended the use of MBCT to patients with current depression described as 'difficult-to-treat'. Preliminary studies investigating MBCT in treatment-resistant and chronic depression were published in 2008 [17] and 2009 [18] and several definitive randomised controlled trials have been conducted since then [19–24] , suggesting that evidence has accumulated to a point where the use of MBCT as a treatment for patients with current DTD can now be considered for guideline endorsement and wider implementation. However, conclusions from individual studies are contrained by heterogeneous definitions and settings, variable comparators and follow-up durations, inconsistent outcome reporting, and limited power for moderator analyses. It seems timely therefore to bring data from existing randomised controlled trials together and analyse outcome across studies.

For this purpose, we have pooled individual patient data (IPD) from these studies to conduct meta-analyses with one-stage random effects meta-analysis of independent patient

For peer review only - http://bmjopen.bmj.com/site/about/guidelines.xhtml

data (IPD) as our primary approach given its advantages over conventional aggregate data meta-analyses [25–28] . This IPD meta-analysis will apply a consistent DTD framework, harmonise outcomes, estimate overall effects using one-stage models, and test prespecified patient-level moderators, with comprehensive risk-of-bias and small-study assessments. The main aim of the analyses is to establish efficacy of MBCT for patients with DTD as compared to the control conditions utilised in the existing trials. Given the need for both effective symptom reduction and the maintenance of gains, we will aim to determine immediate effects at the end of the eight-week MBCT intervention (post-treatment) and effects in the longer-term after patients have finished the intervention and are free to continue engaging in mindfulness practice by themselves (follow-up).

An important advantage of IPD meta-analysis is that it allows to examine moderator or subgroup questions across the pooled sample and can thus serve to overcome limitations in sample size and power for answering these questions in the original studies [29] . Information about potential moderators of treatment effects, such as severity of depression at entry to treatment or a history of bipolar disorder, is important as it can potentially help with implementation by providing more precise information about relative indications or contraindications of the intervention. A further aim of this IPD meta-analysis is therefore to explore effects of potential individual patient level (e.g. sociodemographic and clinical characteristics) and study level (e.g. delivery format) moderators of treatment effects in the pooled data set.

As the clinical concept of DTD does not link to an objective way of assessment, the current meta-analysis will include studies that have used a range of conventional definitions falling under the wider concept of DTD, including selecting participants based on treatment non-response/remission, treatment resistance or the presence of chronic depression. Subgroup analyses will allow us to determine the extent to which effects found in the aggregated data

For peer review only - http://bmjopen.bmj.com/site/about/guidelines.xhtml

generalise across these conventional categories. Given that DTD reflects a wider construct encompassing a large and heterogeneous group of patients with different characteristics and different treatment histories, we will aim to carefully describe the sample both on the meta- and the study-level.

METHODS

General approach

This IPD meta-analysis is registered as part of a broader project on PROSPERO (registration number: CRD42022332039; date of registration in PROSPERO: 24 May 2022) and any key changes or amendments will be documented there. In addition to the direct comparisons between MBCT and control conditions, the wider project also includes studies of an individual psychotherapeutic intervention, Cognitive Behavioral Analysis System of Psychotherapy, and considers a wider range of outcomes, including both beneficial and adverse treatment effects. The current protocol specifies procedures for the comparison between MBCT and the control conditions utilized in the existing MBCT trials only as we assume that this comparison is of particular interest given the need for establishing further treatment options for DTD. The moderator and sensitivity analyses described here were prespecified in this protocol prior to data analysis but were not detailed in the initial PROSPERO entry. They are included to address theoretically and clinically relevant questions, and all analyses (including null results) will be reported to minimise selective reporting. The protocol is registered retrospectively as searches to identify eligible papers were already conducted in 2024, with the last search having been performed on 17 June 2024 and an update search planned immediately before the start of data analysis. Corresponding authors of the selected studies were contacted and asked to provide raw data from their studies. The Preferred Reporting Items for Systematic Reviews and Meta-Analyses IPD statement [30] will be followed for the reporting of this study.

For peer review only - http://bmjopen.bmj.com/site/about/guidelines.xhtml

Eligibility criteria

Types of studies to be included

This IPD meta-analysis will include only peer-reviewed RCTs, published in English. There will be no restriction on the type of recruitment settings. The restriction to English-language publications is due to feasibility constraints and is recognised as a potential source of language bias.

Type of intervention

Included studies will have investigated the effects of manualised mindfulness-based cognitive therapy (MBCT) alone or as an adjunct treatment to treatment-as-usual (TAU) [31]. MBCT is a psychological group-based intervention that combines intensive training in mindfulness meditation with elements of cognitive behavioural therapy (CBT) for depression. Mindfulness can be operationally defined as non-judgemental awareness of present moment experience [32]. As MBCT was originally designed to prevent relapse rather than to reduce symptoms during a current episode, we will accept studies that introduce minor adaptations to the *delivery* of the manualised approach (e.g. incorporating specific support for managing current depressive symptoms, particularly around the engagement in regular practice, and rephrasing psycho-educational components to address current depression rather than relapse, *without altering the structure or core content of the manual*). Other types of mindfulness-based interventions will not be included. MBCT studies will be considered regardless of delivery format (e.g. physical group setting versus videoconference).

Types of comparators

This may include TAU for depression, including pharmacological and psychological treatments (e.g., CBT) or active controls. The latter will include established interventions as well as control interventions designed to mimic effects of mindfulness-based interventions in some or all aspects with the exception of the core element of mindfulness.

For peer review only - http://bmjopen.bmj.com/site/about/guidelines.xhtml

Participants

We will include studies that recruited adults with current Major Depressive Disorder (MDD) [33, 34] and studies which included mixed groups of patients with residual depressive symptoms or full episodes in the context of a chronic or treatment-resistant course of the disorder. In the latter case, participants with only residual symptoms will be excluded from the primary analysis approach (one-stage meta-analysis). As DTD does not link to an objective way of assessment, we will opt for a maximally inclusive interpretation of the term and include any studies that selected patients based on previous treatment non-response/remission, treatment-resistance or chronicity. We will exclude studies with participants below the age of 18 and studies that have exclusively recruited participants over the age of 65 given that the factors involved in the onset, maintenance and recurrence of depression in these age groups can differ from those in adults within this age bracket [35, 36] .

Outcomes

The main type of outcome will be severity of depressive symptomatology as assessed using standard depression outcome measures. Standard depression outcome measures may include self-report measures and observer-rated measures. If both types of measures are available, we will investigate effects on both types of outcome measures. If several measures are available for a single study, preference will be given to the measure that was used as the primary outcome of the original study. To compare outcomes across studies using different outcome measures, we will transform scores from these measures into standardised scores using the common metric developed by Wahl et al. [37] . Where a measure is not part of the common metric approach, we will transform the measure into one that is part of the common metric using published conversion tables [38] , and then derive the common metric.

For peer review only - http://bmjopen.bmj.com/site/about/guidelines.xhtml

To facilitate clinical interpretation of results, we will derive dichotomous outcomes reflecting remission, improvement and deterioration. To apply a common rule for all studies, common metric scores will be converted to the PHQ-9 and dichotomous outcomes computed based on this measure. Following conventions for the PHQ-9 used in pragmatic trials and health services, remission will be defined as a score of less than 10 [39, 40], i.e. a shift from "caseness" to a non-case score. Definition of clinically meaningful change will follow suggestions from recent trials in treatment-resistant depression, which has indicated a threshold of 6 or more points [41, 42] . We will therefore define clinically meaningful improvement as a reduction of 6 or more points and clinically meaningful deterioration as an increase of 6 or more points on the PHQ-9.

A more detailed analysis of negative effects including suicidality is covered in a separate protocol covering a network meta-analysis of MBCT and another individual psychotherapeutic intervention (Michalak et al., in submission). Secondary outcomes such as mindfulness, self-compassion and quality of life will be collected and may be used for future analysis.

Moderators

We will also investigate potential moderators of treatment response, i.e. factors that determine whether a person responds better to MBCT as compared to the control condition or vice versa. These will include potential moderators on the individual patient level (e.g. sociodemographic characteristics, history of childhood trauma, and clinical characteristics) and on the study level. We will include patient level characteristics in the analyses, if they are reported across a sufficient number of studies and datasets (see statistical analysis section for criteria).

Sociodemographic and clinical characteristics that we aim to investigate include age [43] , gender [44] , marital status [45] , education [46] , employment [47] , childhood

For peer review only - http://bmjopen.bmj.com/site/about/guidelines.xhtml

adversity [48] , age at onset of depression [49] , number of previous episodes [50] , duration of current episode [51] , current suicidality [52] , current antidepressants [53] , past antidepressants and treatment-resistance to antidepressants [54] , co-morbid anxiety disorder, comorbid obsessive-compulsive disorder, co-morbid post-traumatic stress disorder, co-morbid substance abuse or dependence, co-morbid eating disorder, co-morbid personality disorder [43], and level of depressive symptoms at entry [55] . Study level characteristics will include type of delivery (remote versus face-to-face), type of control condition (TAU, active control), type of DTD (non-response/remission, treatment-resistance, chronic depression) and study quality.

Timing of outcome assessments

As follow-up periods of the studies differ, we will group follow-up periods in the following categories: 8 to 10 weeks post-randomisation (post-treatment), 11 to 34 weeks post-randomisation (follow-up 1), and 35 to 60 weeks post-randomisation (follow-up 2).

Searches and study selection

We searched PubMed, Web of Science, Scopus, PsychINFO, EMBASE, and the Cochrane Controlled Trials Register using index and free terms, jointly with Boolean operators, on four tiers, namely: (1) depressive disorder, (2) non-response, treatment resistance and chronicity, (3) mindfulness-based cognitive therapy, and (4) randomised-controlled trial. The search terms used for the different databases are shown in the appendix. We did not search grey literature or trial registries beyond the Cochrane Controlled Trials Register, and we did not consult experts outside of the research team.

We searched for any study published up until the day of the search, on 17 June 2024. There was no lower time limit. Initial screening of titles was performed by two independent reviewers (TB, JM), with the publication abstract being obtained if selected by at least one reviewer. Abstracts were screened by the same two independent reviewers, with

For peer review only

disagreements about eligibility being referred to a third reviewer whose decision was final. An update search will be conducted immediately before the start of data analysis.

Risk of bias assessment

The validity of the studies will be assessed using the Risk of Bias tool by the Cochrane Collaboration (version 2) [56, 57] . Possible sources of bias assessed by the tool are (1) random sequence generation, (2) allocation concealment, (3) blinding of outcome assessors, (4) incomplete outcome data, (5) selective outcome reporting, and (6) other threats to validity including lack of compliance and different timing of outcome assessments. Data from the published papers of the trials will be evaluated by two independent assessors. Agreement rate will be reported, and any disagreements will be solved through discussion. A sensitivity analysis will be performed excluding studies judged to be at high risk of bias, or where risk of bias is unclear.

Patient and public involvement

A lived experience advisory group, led by MR and consisting of experts who have been involved in previous trials of MBCT, will be consulted for the interpretation of study results and their dissemination. A coproduced lay summary of results will be written for dissemination alongside papers for peer review journals.

IPD data collection and aggregation

All corresponding authors of the articles selected for inclusion were contacted via email by TB with an invitation to participate in the review and to share the IPD from their primary study. The standard letter described all relevant details of the study including its purpose, main research questions, and the variables of interest and ended with a request to share the raw data from the study. Corresponding authors of studies were invited to serve as collaborators for the project. If corresponding authors did not respond within four weeks, we sent a second email request. If this had failed, a second author was contacted. Apart from one

For peer review only - http://bmjopen.bmj.com/site/about/guidelines.xhtml

study, whose authors could not be contacted, none of the selected studies required contact attempts beyond a second author and the responding authors agreed to share their data.

Data checking and integrity

Authors were requested to provide a codebook or data dictionary explaining variable names, coding of values, measurement units etc. After accepting the invitation to collaborate and signing the data transfer agreement, the study authors shared their IPD via secure data transfer portal at the University of Surrey. Data is stored securely on University of Surrey servers in accordance with the European Union's General Data Protection Regulation Act and the UK's Data Protection Act 2018.

TB and MH will take primary responsibility for preparing and extracting outcome data and other characteristics, with MV independently checking extractions for accuracy. Received datasets will be reviewed to assess completeness and checked against the reported data from relevant study publications. The numbers of participants, overall and by arm, will be confirmed to correspond with the numbers in the study publication. Ranges of values will be inspected for clearly erroneous data. Baseline participant demographic data will be inspected by arm, with the aim of checking that the IPD matches the data reported in the publication, in terms of the numbers of participants with reported data, and overall aggregated values for each variable. Discrepancies will be referred to the study authors for investigation and any remaining discrepancies will be recorded. For each included trial, we will extract information on funding sources and any reported conflicts of interest, and this information will be presented alongside other study characteristics.

Although the main search and initial receipt of IPD occurred before the submission of this protocol for publication, the protocol was registered in PROSPERO (CRD42022332039) beforehand. No data analysis or outcome-driven decisions were made prior to finalizing the protocol, and the methods described here were pre-specified prior to data inspection.

For peer review only - http://bmjopen.bmj.com/site/about/guidelines.xhtml

Data harmonization

Once datasets from the individual studies have been checked and outcomes standardised, they will be merged into the final dataset. The final dataset for analysis will be checked again for accuracy by a researcher in the study team.

STATISTICAL ANALYSIS

To finalise the selection of moderator variables, we will determine presence of these variables across the individual data sets and decide on ways to standardise these variables where needed, for example by collapsing categories. Potential moderator variables will be included in the analyses if the variable has at least 40% available data in the final dataset and has been recorded by at least 4 studies.

To investigate the efficacy of MBCT compared to control conditions and test moderating effects of individual patient level and study level characteristics, we will conduct one-stage and two-stage IPD meta-analyses (IPD-MAs). Random-effects models will be used throughout because we expect high statistical heterogeneity due to variation in control groups (treatment as usual, active controls) and study populations (treatment non-responders/remitters, treatment-resistance, chronic depression).

The main research question of the study (whether MBCT is efficacious compared to control conditions) will be addressed via a maximum of three pairwise comparisons: (1) MBCT versus treatment-as-usual controls, (2) MBCT versus active controls, and (3) MBCT versus all controls.

By default, all analyses will be conducted in a Bayesian framework using JAGS (Just Another Gibbs Sampler)/BUGS (Bayesian analysis Using Gibbs Sampling) [58]. We chose a Bayesian approach because it offers greater flexibility in the parametrization of the (one-stage) IPD-MA models, and because this allows for a more intuitive interpretation of results

For peer review only - http://bmjopen.bmj.com/site/about/guidelines.xhtml

based on posterior tail probabilities [59] . Across all analyses, findings will be considered "significant" when the credibility interval of the relevant effect does not include zero.

Two-stage random effects meta-analysis

We will first conduct a conventional meta-analysis using data extracted from the published papers. We will calculate Hedges' g to determine the effect sizes of the difference between the intervention and the control conditions reported in the papers. To investigate heterogeneity of effect sizes, we will calculate I^2, the percentage of variation not attributable to sampling error, as well as 95% prediction intervals (PIs) around the pooled effect [60, 61] . Leave-one-out analyses will be conducted to identify potential outliers and/or influential studies. Small-study effects will be investigated visually using funnel plot inspection and the Egger's test for asymmetry. A weakly informative Half-Cauchy (0,0.5) prior will be used for the between-study heterogeneity variance τ^2 [62], and flat normal priors for all other parameters. A detailed justification for this prior setup has been derived in previous work [63].

One-stage random effects meta-analysis using IPD

One-stage IPD meta-analysis models will be used as the primary analytic approach. To analyse the effects on common metrics-converted symptom severity scores, an identity-link linear mixed effects model with stratified intercepts and trial-specific (heteroscedastic) error terms will be used. Let y_{ik} be the depressive symptom severity of patient i in trial k (with k=1, …, K studies included in the meta-analysis). The one-stage IPD can then be defined as:

$$y_{ik} = \alpha_k + \theta T_{ik} + u_k T_{ik} + \beta_k \left(X_{ik} - \overline{X}_{ik}\right) + \varepsilon_{ik} \qquad (1)$$

$$u_k \sim \mathcal{N}\left(0,\tau_\theta^2\right) \qquad \varepsilon_{ik} \sim \mathcal{N}\left(0,\sigma_k^2\right)$$

Where T_{ik} is the treatment indicator, X_{ik} is the symptom severity score at baseline, $\overline{X}_{ik}$ is the mean of X for each specific trial, α_k is the stratified trial intercept, and u_k is the

For peer review only - http://bmjopen.bmj.com/site/about/guidelines.xhtml

trial-specific random slope of the average treatment effect θ. For this model, priors mirroring the two-stage approach will be used throughout, with a uniform distribution assigned to σ_k^2.

The same model specification, but with a logit link, will be used to model the effects on binary outcomes, including remission, clinically meaningful improvement and clinically meaningful deterioration based on PHQ-9 thresholds:

$$\log_e\left(\frac{\pi_{ik}}{1-\pi_{ik}}\right) = \alpha_k + \theta T_{ik} + u_k T_{ik} + \beta_k\left(X_{ik} - \bar{X}_{ik}\right) + \varepsilon_{ik}$$
$$y_{ik} \sim \text{Bern}(\pi_{ik}) \quad u_k \sim \mathcal{N}\left(0,\tau_\theta^2\right) \quad \varepsilon_{ik} \sim \mathcal{N}\left(0,\sigma_k^2\right) \qquad (2)$$

Where y_{ik} now represents the patient's binary outcome of interest. Numbers needed to treat (*NNT*) corresponding to the estimated SMD values will be calculated using the method by Furukawa and Leucht [64], and as the inverse of the absolute risk reduction otherwise.

Moderator analyses

We will explore potential moderators of treatment effect by adding individual participant level and study level treatment-covariate interactions into the main IPD-MA model. To optimise power, these analyses will be done on treatment effects at post-treatment as all studies can provide data for this timepoint.

Missing data

We will record the percentage of individual participant missing data for baseline characteristics and outcomes in each of the studies included in the meta-analyses. All analyses will be conducted according to the "intention-to-treat" principle, so that analyses target a treatment policy estimand [65]. Missing data will be handled using multiple imputation (fully conditional specification; MICE algorithm [66] under the missing at random (MAR) assumption. For the main effectiveness analysis, multilevel two-stage imputation models with heteroscedastic errors will be used to account for the nested data

For peer review only - http://bmjopen.bmj.com/site/about/guidelines.xhtml

structure [67]. Highly collinear variables will be removed as predictors, as well as variables with systematically missing information ("structural zeros"). A total of *m*=50 imputation sets will be generated. A separate imputation model will be constructed for each focal moderator, including auxiliary variables with a maximum of 10% missingness. Imputations will be generated separately for each of the treatment groups (intervention, control) to account for treatment-covariate interactions. All analysis models will be fitted in the multiply imputed data. Parameter estimates and test statistics will then be aggregated by mixing the draws from the posterior distribution of each completed dataset [68]. Sensitivity analyses will be performed to compare results using observed and imputed data [69] .

Subgroup analyses

We plan to examine the efficacy of MBCT and potential moderators of treatment outcomes in subgroups defined by the conventional descriptive categories used by the individual studies to define their populations, distinguishing between studies that recruited patients characterised as treatment non-responders/remitters, treatment-resistant, or as suffering from chronic depression. These analyses will be based on subsamples created from the final IPD dataset.

Sensitivity analyses

As a sensitivity analysis, we will repeat all main effectiveness analyses using equivalent frequentist (one- and two-stage) approaches. Additionally, we will conduct sensitivity analyses to investigate whether sources of heterogeneity affect the overall effect size estimate and robustness of our findings. This will include exploring the role of risk of bias and other potential study level sources of heterogeneity that may become obvious after data have been examined. These analyses will be conducted using a two-stage IPD-MA model.

DISCUSSION

For peer review only - http://bmjopen.bmj.com/site/about/guidelines.xhtml

For peer review only

This IPD meta-analysis will provide estimates of efficacy for the use of MBCT in DTD and offer comprehensive information about potential moderators of treatment effects. As such it will contribute to arguments in favour of or against implementing MBCT as a treatment for DTD and may help to decide whether MBCT is a recommendable treatment option for this group. This is important given the need to improve the care and management of DTD and the increasing recognition that psychological therapies can have an integral role in this endeavour. Information about potential moderators of treatment effects will help to refine the understanding of who may or may not benefit from MBCT for DTD.

We have chosen the emerging concept of DTD as an umbrella because it provides a broad conceptualisation of depression that remains burdensome despite usual treatment attempts. Most available trials have operationalised this construct using narrower criteria such as non-response, non-remission, treatment-resistance, or chronicity, and our inclusion criteria therefore reflect how DTD has been studied to date. These characteristics represent core manifestations of DTD, and their use ensures consistency and feasibility for the planned meta-analysis. We acknowledge, however, that DTD extends beyond these dimensions, for example in its emphasis on functional outcomes, and generalisability of findings to the broader construct should be interpreted with this in mind.

The chosen approach using IPD has several advantages over conventional meta-analyses including the possibility to assess whether individual participant level characteristics influence treatment effects with more specificity and power. Limitations of the approach include the fact that inconsistencies across studies regarding the format and type of variables reported may restrict our ability to investigate moderators of treatment effects and that transformation of outcome measures to a common metric across all studies may come with a small loss of information. To address this latter point, the current study will complement one-stage IPD analyses with a conventional two-stage approach. A more general concern that is

For peer review only - http://bmjopen.bmj.com/site/about/guidelines.xhtml

often raised for IPD meta-analyses is inclusion bias as it may be difficult to obtain IPD for all selected studies.

This IPD meta-analysis is registered as part of a broader project that examines a wider range of interventions and outcomes, including both beneficial and adverse effects. The present protocol specifies procedures for the comparison between MBCT and control conditions in MBCT trials, as we assume that this comparison is of particular interest given the need to establish interventions for DTD. To our knowledge, this is the first IPD meta-analysis to investigate the effects of MBCT in DTD and assess moderators of treatment effects. As evidence from trials of MBCT for DTD has now accumulated to a point where the use of MBCT for DTD can be considered for endorsement in treatment guidelines, findings from this IPD meta-analysis have the potential to inform treatment recommendations and decisions regarding implementation. The results will provide clinicians, healthcare providers and people who are suffering from DTD with information on the characteristics that may make it more or less likely for patients to benefit from this treatment and thus facilitate implementation.

ETHICS AND DISSEMINATION

No local ethical review was necessary following consultation with the Ethics and Governance Board of the University of Surrey. The investigators of the primary trials have local ethical approval for sharing the data. TB and MH will be responsible for oversight of the database and analyses will be conducted by MH and TB. Patient privacy will be ensured by adhering to University of Surrey guidance on data management and storage. Exchange of data is governed by inter-institutional data sharing agreements. All data were pseudonymised (de-identified) before sharing, shared through secure upload facilities and are stored on secure servers. The data set will not be open access. Researchers of good standing may receive access pending on institutional approvals on data transfer.

For peer review only - http://bmjopen.bmj.com/site/about/guidelines.xhtml

We will disseminate the work in peer-reviewed journals, provide lay summaries and discuss findings with patient and lived experience groups. Other means of communication to disseminate the results will include conference presentations and social media announcements.

CONTRIBUTIONS

TB, MN, JM, MV and MH conceptualised and designed the study. TB developed the search strategy and contacted the primary authors, who contributed to further refinement of the design and approach of the study. TB, MV and MH are responsible for building the database. MH provides statistical expertise and is responsible for data analyses. TB drafted the protocol manuscript with statistical input by MH, which was critically revised by all authors. All authors read and approved the final version. TB serves as the guarantor of the review.

FUNDING STATEMENT

This research received no specific grant from any funding agency in the public, commercial or not-for-profit sectors.

ACKNOWLEDGMENTS

JMM has a ‘Miguel Servet’ research contract from the ISCIII (CP21/00080), and he is grateful to the Spanish CIBER of Epidemiology and Public Health (CBERESP CB22/02/00052< ISCIII) for their support.

COMPETING INTERESTS STATEMENT

TB is the co-author of a book on mindfulness-based interventions and regularly offers workshops on mindfulness-based interventions. He is the co-investigator of a programme grant evaluating an adapted MBCT course for adolescents experiencing depression.

MN is author of a book chapter on mindfulness-based interventions and offers workshops on mindfulness-based interventions.

For peer review only - http://bmjopen.bmj.com/site/about/guidelines.xhtml

JM is the director of the Achtsamkeitsinstitut Ruhr (an institute offering mindfulness training) and principal investigator of several DFG (German Science Foundation) research projects. He receives royalties from mindfulness books he has authored.

MV reports no conflicts of interest.

JMGW co-developed MBCT, and was founding Director of the University of Oxford Mindfulness Centre. He receives royalties from books describing the MBCT program. He also receives honoraria from lectures and training workshops on mindfulness.

AC leads a professional practice (Istituto Mente Corpo) that offers courses of mindfulness-based interventions.

SE is the author of book on Mindfulness-Based Cognitive Therapy for the treatment of chronic and treatment-resistant depression.

KD declares no conflicts of interest.

ZS received book royalties from Guilford Press, workshop fees from the Centre for Mindfulness studies, and revenue from online sales at Mindful Noggin Inc., which are all related to his work as a co-founder of Mindfulness-Based Cognitive Therapy (MBCT).

MCM declares no conflicts of interest.

AS is the founder and director of the Radboudumc Expertise Centre for Mindfulness and co-investigator of a number of studies examining the effectiveness and implementation of mindfulness-based interventions for patients with psychological or somatic disorders and health care professionals.

AAF declares no conflicts of interest.

MGT declares no conflicts of interest.

JMM is associated with the University of Oxford Mindfulness Research Centre.

BDD is the lead of the University of Exeter AccEPT clinic, which offers courses of MBCT.

For peer review only - http://bmjopen.bmj.com/site/about/guidelines.xhtml

CS is a member of a training organisation that has been commissioned by NHS England to deliver MBCT training across NHS Talking Therapies services in England and co-leads the Sussex Mindfulness Centre. CS is a co-investigator of a programme grant evaluating an adapted MBCT course for adolescents experiencing depression and co-investigator on a grant evaluating an adapted MBCT course for NHS staff.

FAR regularly provides workshops on mindfulness-based interventions.

MR declares no conflicts of interest.

MH serves as a statistical consultant for HelloBetter/GetOn Institut für Gesundheitstrainings GmbH, a company that implements digital mental health therapeutics in routine care.

REFERENCES

1. Collaborators G 2019 MD. Global, regional, and national burden of 12 mental disorders in 204 countries and territories, 1990–2019: a systematic analysis for the Global Burden of Disease Study 2019. Lancet Psychiatry. 2022;9:137–50. https://doi.org/10.1016/s2215-0366(21)00395-3.

2. McIntyre RS, Alsuwaidan M, Baune BT, Berk M, Demyttenaere K, Goldberg JF, et al. Treatment-resistant depression: definition, prevalence, detection, management, and investigational interventions. World Psychiatry. 2023;22:394–412. https://doi.org/10.1002/wps.21120.

3. Rush AJ, Sackeim HA, Conway CR, Bunker MT, Hollon SD, Demyttenaere K, et al. Clinical research challenges posed by difficult-to-treat depression. Psychol Med. 2022;52:419–32. https://doi.org/10.1017/s0033291721004943.

4. McAllister-Williams RH, Arango C, Blier P, Demyttenaere K, Falkai P, Gorwood P, et al. The identification, assessment and management of difficult-to-treat depression: An international consensus statement. J Affect Disord. 2020;267:264–82. https://doi.org/10.1016/j.jad.2020.02.023.

5. Jaffe DH, Rive B, Denee TR. The humanistic and economic burden of treatment-resistant depression in Europe: a cross-sectional study. BMC Psychiatry. 2019;19:247. https://doi.org/10.1186/s12888-019-2222-4.

For peer review only - http://bmjopen.bmj.com/site/about/guidelines.xhtml

6. Bhattacharya R, Shen C, Sambamoorthi U. Excess risk of chronic physical conditions associated with depression and anxiety. BMC Psychiatry. 2014;14. https://doi.org/10.1186/1471-244x-14-10.

7. Moeller SB, Gbyl K, Hjorthøj C, Andreasen M, Austin SF, Buchholtz PE, et al. Treatment of difficult-to-treat depression – clinical guideline for selected interventions. Nord J Psychiatry. 2022;76:177–88. https://doi.org/10.1080/08039488.2021.1952303.

8. Markowitz JC, Wright JH, Peeters F, Thase ME, Kocsis JH, Sudak DM. The Neglected Role of Psychotherapy for Treatment-Resistant Depression. Am J Psychiatry. 2022;179:90–3. https://doi.org/10.1176/appi.ajp.2021.21050535.

9. McHugh RK, Whitton SW, Peckham AD, Welge JA, Otto MW. Patient Preference for Psychological vs Pharmacologic Treatment of Psychiatric Disorders: A Meta-Analytic Review. J Clin Psychiatry. 2013;74:595–602. https://doi.org/10.4088/jcp.12r07757.

10. Segal ZV, Williams JMG, Teasdale J. Mindfulness-Based Cognitive Therapy for Depression. 2013.

11. Barnhofer T, Dunn BD, Strauss C, Ruths FA, Barrett B, Ryan M, et al. Mindfulness-based cognitive therapy versus treatment as usual after non-remission with NHS Talking Therapies high-intensity psychological therapy for depression: a UK-based clinical effectiveness and cost-effectiveness randomised, controlled, superiority trial. Lancet Psychiatry. 2025;12:433–46. https://doi.org/10.1016/s2215-0366(25)00105-1.

12. Kuyken W, Warren FC, Taylor RS, Whalley B, Crane C, Bondolfi G, et al. Efficacy of Mindfulness-Based Cognitive Therapy in Prevention of Depressive Relapse: An Individual Patient Data Meta-analysis From Randomized Trials. Jama Psychiat. 2016;73:565. https://doi.org/10.1001/jamapsychiatry.2016.0076.

13. Ma SH, Teasdale JD. Mindfulness-Based Cognitive Therapy for Depression: Replication and Exploration of Differential Relapse Prevention Effects. J Consult Clin Psych. 2004;72:31–40. https://doi.org/10.1037/0022-006x.72.1.31.

14. Teasdale JD, Segal ZV, Williams JMG, Ridgeway VA, Soulsby JM, Lau MA. Prevention of relapse/recurrence in major depression by mindfulness-based cognitive therapy. J Consult Clin Psych. 2000;68:615–23. https://doi.org/10.1037/0022-006x.68.4.615.

15. Creswell JD, Lindsay EK. How Does Mindfulness Training Affect Health? A Mindfulness Stress Buffering Account. Current Directions in Psychological Science. 2014;23. https://doi.org/10.1177/0963721414547415.

16. Lindsay EK, Creswell DJ. Mechanisms of mindfulness training: Monitor and Acceptance Theory (MAT). Clinical Psychology Review. 2017;51. https://doi.org/10.1016/j.cpr.2016.10.011.

17. Eisendrath SJ, Delucchi K, Bitner R, Fenimore P, Smit M, McLane M. Mindfulness-Based Cognitive Therapy for Treatment-Resistant Depression: A Pilot Study. Psychother Psychosom. 2008;77:319–20. https://doi.org/10.1159/000142525.

For peer review only - http://bmjopen.bmj.com/site/about/guidelines.xhtml

18. Barnhofer T, Crane C, Hargus E, Amarasinghe M, Winder R, Williams JMG. Mindfulness-based cognitive therapy as a treatment for chronic depression: A preliminary study. Behav Res Ther. 2009;47:366–73. https://doi.org/10.1016/j.brat.2009.01.019.

19. Eisendrath SJ, Gillung E, Delucchi KL, Segal ZV, Nelson JC, McInnes LA, et al. A Randomized Controlled Trial of Mindfulness-Based Cognitive Therapy for Treatment-Resistant Depression. Psychother Psychosom. 2016;85:99–110. https://doi.org/10.1159/000442260.

20. Cladder-Micus MB, Speckens AEM, Vrijsen JN, Donders ART, Becker ES, Spijker J. Mindfulness-based cognitive therapy for patients with chronic, treatment-resistant depression: A pragmatic randomized controlled trial. Depress Anxiety. 2018;35:914–24. https://doi.org/10.1002/da.22788.

21. Michalak J, Schultze M, Heidenreich T, Schramm E. A randomized controlled trial on the efficacy of mindfulness-based cognitive therapy and a group version of cognitive behavioral analysis system of psychotherapy for chronically depressed patients. J Consult Clin Psych. 2015;83:951–63. https://doi.org/10.1037/ccp0000042.

22. Chiesa A, Castagner V, Andrisano C, Serretti A, Mandelli L, Porcelli S, et al. Mindfulness-based cognitive therapy vs. psycho-education for patients with major depression who did not achieve remission following antidepressant treatment. Psychiat Res. 2015;226:474–83. https://doi.org/10.1016/j.psychres.2015.02.003.

23. Garcia A, Yáñez AM, Bennasar-Veny M, Navarro C, Salva J, Ibarra O, et al. Efficacy of an adjuvant non-face-to-face multimodal lifestyle modification program for patients with treatment-resistant major depression: A randomized controlled trial. Psychiatry Res. 2023;319:114975. https://doi.org/10.1016/j.psychres.2022.114975.

24. Foroughi A, Sadeghi K, Parvizifard A, Moghadam AP, Davarinejad O, Farnia V, et al. The effectiveness of mindfulness-based cognitive therapy for reducing rumination and improving mindfulness and self-compassion in patients with treatment-resistant depression. Trends Psychiatry Psychotherapy. 2020;42:138–46. https://doi.org/10.1590/2237-6089-2019-0016.

25. Mathew T, Nordström K. Comparison of One-Step and Two-Step Meta-Analysis Models Using Individual Patient Data. Biom J. 2010;52:271–87. https://doi.org/10.1002/bimj.200900143.

26. Clarke MJ. Individual patient data meta-analyses. Best Pr Res Clin Obstet Gynaecol. 2005;19:47–55. https://doi.org/10.1016/j.bpobgyn.2004.10.011.

27. Riley RD, Lambert PC, Abo-Zaid G. Meta-analysis of individual participant data: rationale, conduct, and reporting. BMJ. 2010;340 feb05 1:c221. https://doi.org/10.1136/bmj.c221.

28. Jones AP, Riley RD, Williamson PR, Whitehead A. Meta-analysis of individual patient data versus aggregate data from longitudinal clinical trials. Clin Trials. 2009;6:16–27. https://doi.org/10.1177/1740774508100984.

For peer review only - http://bmjopen.bmj.com/site/about/guidelines.xhtml

29. Cooper H, Patall EA. The Relative Benefits of Meta-Analysis Conducted With Individual Participant Data Versus Aggregated Data. Psychol Methods. 2009;14:165–76. https://doi.org/10.1037/a0015565.

30. Stewart LA, Clarke M, Rovers M, Riley RD, Simmonds M, Stewart G, et al. Preferred Reporting Items for a Systematic Review and Meta-analysis of Individual Participant Data: The PRISMA-IPD Statement. Jama. 2015;313:1657–65. https://doi.org/10.1001/jama.2015.3656.

31. Löfholm CA, Brännström L, Olsson M, Hansson K. Treatment-as-usual in effectiveness studies: What is it and does it matter? Int J Soc Welf. 2013;22:25–34. https://doi.org/10.1111/j.1468-2397.2012.00870.x.

32. Kabat-Zinn J. Mindfulness-Based Interventions in Context: Past, Present, and Future. Clinical Psychology: Science and Practice. 2003;10:144–56. https://doi.org/10.1093/clipsy.bpg016.

33. Association AP. Diagnostic and statistical manual of mental disorders (5th ed.). Arlington, VA: Author; 2013.

34. Association AP. Diagnostic and statistical manual of mental disorders (4th ed.). Washington, DC: Author; 2000.

35. Korten NCM, Comijs HC, Lamers F, Penninx BWJH. Early and late onset depression in young and middle aged adults: Differential symptomatology, characteristics and risk factors? J Affect Disord. 2012;138:259–67. https://doi.org/10.1016/j.jad.2012.01.042.

36. Devanand DP, Adorno E, Cheng J, Burt T, Pelton GH, Roose SP, et al. Late onset dysthymic disorder and major depression differ from early onset dysthymic disorder and major depression in elderly outpatients. J Affect Disord. 2004;78:259–67. https://doi.org/10.1016/s0165-0327(02)00307-5.

37. Wahl I, Löwe B, Bjorner JB, Fischer F, Langs G, Voderholzer U, et al. Standardization of depression measurement: a common metric was developed for 11 self-report depression measures. J Clin Epidemiology. 2014;67:73–86. https://doi.org/10.1016/j.jclinepi.2013.04.019.

38. Rush AJ, Trivedi MH, Ibrahim HM, Carmody TJ, Arnow B, Klein DN, et al. The 16-Item quick inventory of depressive symptomatology (QIDS), clinician rating (QIDS-C), and self-report (QIDS-SR): a psychometric evaluation in patients with chronic major depression. Biol Psychiatry. 2003;54:573–83. https://doi.org/10.1016/s0006-3223(02)01866-8.

39. Kroenke K, Spitzer RL, Williams JBW. The PHQ-9: Validity of a brief depression severity measure. J Gen Intern Med. 2001;16:606–13. https://doi.org/10.1046/j.1525-1497.2001.016009606.x.

40. Löwe B, Unützer J, Callahan CM, Perkins AJ, Kroenke K. Monitoring Depression Treatment Outcomes With the Patient Health Questionnaire-9. Med Care. 2004;42:1194–201. https://doi.org/10.1097/00005650-200412000-00006.

For peer review only - http://bmjopen.bmj.com/site/about/guidelines.xhtml

41. Hudgens S, Floden L, Blackowicz M, Jamieson C, Popova V, Fedgchin M, et al. Meaningful Change in Depression Symptoms Assessed with the Patient Health Questionnaire (PHQ-9) and Montgomery-Åsberg Depression Rating Scale (MADRS) Among Patients with Treatment Resistant Depression in Two, Randomized, Double-blind, Active-controlled Trials of Esketamine Nasal Spray Combined With a New Oral Antidepressant. J Affect Disord. 2021;281:767–75. https://doi.org/10.1016/j.jad.2020.11.066.

42. Turkoz I, Alphs L, Singh J, Jamieson C, Daly E, Shawi M, et al. Clinically meaningful changes on depressive symptom measures and patient-reported outcomes in patients with treatment-resistant depression. Acta Psychiatr Scand. 2021;143:253–63. https://doi.org/10.1111/acps.13260.

43. Hölzel L, Härter M, Reese C, Kriston L. Risk factors for chronic depression — A systematic review. J Affect Disorders. 2011;129:1–13. https://doi.org/10.1016/j.jad.2010.03.025.

44. Seedat S, Scott KM, Angermeyer MC, Berglund P, Bromet EJ, Brugha TS, et al. Cross-National Associations Between Gender and Mental Disorders in the World Health Organization World Mental Health Surveys. Arch Gen Psychiatry. 2009;66:785–95. https://doi.org/10.1001/archgenpsychiatry.2009.36.

45. Buckman JEJ, Saunders R, Stott J, Arundell L-L, O'Driscoll C, Davies MR, et al. Role of age, gender and marital status in prognosis for adults with depression: An individual patient data meta-analysis. Epidemiology Psychiatr Sci. 2021;30:e42. https://doi.org/10.1017/s2045796021000342.

46. Trivedi MH, Rush AJ, Wisniewski SR, Nierenberg AA, Warden D, Ritz L, et al. Evaluation of Outcomes With Citalopram for Depression Using Measurement-Based Care in STARD: Implications for Clinical Practice. Am J Psychiatry. 2006;163:28–40. https://doi.org/10.1176/appi.ajp.163.1.28.

47. Oosten AJ van, Mens K van, Blonk RWB, Burdorf A, Tiemens B. The relationship between having a job and the outcome of brief therapy in patients with common mental disorders. BMC Psychiatry. 2023;23:910. https://doi.org/10.1186/s12888-023-05418-z.

48. Nanni V, Uher R, Danese A. Childhood Maltreatment Predicts Unfavorable Course of Illness and Treatment Outcome in Depression: A Meta-Analysis. Am J Psychiat. 2012;169:141–51. https://doi.org/10.1176/appi.ajp.2011.11020335.

49. Klein DN, Schatzberg AF, McCullough JP, Dowling F, Goodman D, Howland RH, et al. Age of onset in chronic major depression: relation to demographic and clinical variables, family history, and treatment response. J Affect Disord. 1999;55:149–57. https://doi.org/10.1016/s0165-0327(99)00020-8.

50. Bromberger JT, Schott L, Kravitz HM, Joffe H. Risk factors for major depression during midlife among a community sample of women with and without prior major depression: are they the same or different? Psychol Med. 2015;45:1653–64. https://doi.org/10.1017/s0033291714002773.

For peer review only - http://bmjopen.bmj.com/site/about/guidelines.xhtml

51. Myklebost SB, Gjestad R, Inal Y, Hammar Å, Nordgreen T. Predictors of Treatment Response to an Internet-Delivered Intervention Targeting Residual Cognitive Symptoms After Major Depressive Disorder. Front Psychiatry. 2022;13:795698. https://doi.org/10.3389/fpsyt.2022.795698.

52. Courtney DB, Watson P, Krause KR, Chan BWC, Bennett K, Gunlicks-Stoessel M, et al. Predictors, Moderators, and Mediators Associated With Treatment Outcome in Randomized Clinical Trials Among Adolescents With Depression. JAMA Netw Open. 2022;5:e2146331. https://doi.org/10.1001/jamanetworkopen.2021.46331.

53. Cuijpers P, Sijbrandij M, Koole SL, Andersson G, Beekman AT, Reynolds CF. Adding psychotherapy to antidepressant medication in depression and anxiety disorders: a meta-analysis. World Psychiatry. 2014;13:56–67. https://doi.org/10.1002/wps.20089.

54. DeRubeis RJ, Hollon SD, Amsterdam JD, Shelton RC, Young PR, Salomon RM, et al. Cognitive Therapy vs Medications in the Treatment of Moderate to Severe Depression. Arch Gen Psychiatry. 2005;62:409–16. https://doi.org/10.1001/archpsyc.62.4.409.

55. Button KS, Wiles NJ, Lewis G, Peters TJ, Kessler D. Factors associated with differential response to online cognitive behavioural therapy. Soc Psychiatry Psychiatr Epidemiology. 2012;47:827–33. https://doi.org/10.1007/s00127-011-0389-1.

56. Furlan AD, Pennick V, Bombardier C, Tulder M van, Group EB Cochrane Back Review. 2009 Updated Method Guidelines for Systematic Reviews in the Cochrane Back Review Group. Spine. 2009;34:1929–41. https://doi.org/10.1097/brs.0b013e3181b1c99f.

57. Sterne JAC, Savović J, Page MJ, Elbers RG, Blencowe NS, Boutron I, et al. RoB 2: a revised tool for assessing risk of bias in randomised trials. Bmj. 2019;366:l4898. https://doi.org/10.1136/bmj.l4898.

58. Lunn D, Jackson C, Best N, Thomas A, Spiegelhalter D. The BUGS Book, A Practical Introduction to Bayesian Analysis. 2019. https://doi.org/10.1201/b13613.

59. Goligher EC, Heath A, Harhay MO. Bayesian statistics for clinical research. Lancet. 2024;404:1067–76. https://doi.org/10.1016/s0140-6736(24)01295-9.

60. Higgins JPT, Thompson SG, Deeks JJ, Altman DG. Measuring inconsistency in meta-analyses. BMJ. 2003;327:557–60. https://doi.org/10.1136/bmj.327.7414.557.

61. Higgins JPT, Thompson SG. Quantifying heterogeneity in a meta-analysis. Stat Med. 2002;21:1539–58. https://doi.org/10.1002/sim.1186.

62. Harrer M, Cuijpers P, Furukawa TA, Ebert DD. Doing Meta-Analysis with R, A Hands-On Guide. 2022. https://doi.org/10.1201/9781003107347.

63. Harrer M. Heterogeneity Prior Justification. 2022. https://osf.io/3tfm6. Accessed 16 May 2025.

64. Furukawa TA, Leucht S. How to Obtain NNT from Cohen's d: Comparison of Two Methods. PLoS ONE. 2011;6:e19070. https://doi.org/10.1371/journal.pone.0019070.

For peer review only - http://bmjopen.bmj.com/site/about/guidelines.xhtml

65. Han S, Zhou X. Defining estimands in clinical trials: A unified procedure. Stat Med. 2023;42:1869–87. https://doi.org/10.1002/sim.9702.

66. Buuren van, Groothuis-Oudshoorn K. mice: multivariate imputation by chained equations in R. Journal of Statistical Software. 2011;45:1–67.

67. Resche-Rigon M, White IR. Multiple imputation by chained equations for systematically and sporadically missing multilevel data. Stat Methods Méd Res. 2018;27:1634–49. https://doi.org/10.1177/0962280216666564.

68. Zhou X, Reiter JP. A Note on Bayesian Inference After Multiple Imputation. Am Stat. 2010;64:159–63. https://doi.org/10.1198/tast.2010.09109.

69. Burgess S, White IR, Resche-Rigon M, Wood AM. Combining multiple imputation and meta-analysis with individual participant data. Stat Med. 2013;32:4499–514. https://doi.org/10.1002/sim.5844.

For peer review only

APPENDIX

Search Terms

Database	Search Term
Web of Science	(“MDE” OR “major depressive episode” OR “MDD” OR “major depressive disorder” OR “major depression” OR “depression” OR “dysthymia” OR “dysthymic”) AND (“chronic” OR “persistent” OR “treatment-resistant” OR “non-responders” OR “non-response” OR “non-remitted” OR “dysthymia” OR “dysthymic”) AND (“MBCT” OR "mindfulness-based cognitive therapy") AND (“randomized controlled trial” OR “randomised controlled trial” OR “randomized-controlled trial” OR “randomised-controlled trial” OR “RCT” OR “randomized” or “randomised” OR “randomly allocated” OR “randomly assigned”)
Scopus	(“MDE” OR “major depressive episode” OR “MDD” OR “major depressive disorder” OR “major depression” OR “depression” OR “dysthymia” OR “dysthymic”) AND (“chronic” OR “persistent” OR “treatment-resistant” OR “non-responders” OR “non-response” OR “non-remitted” OR “dysthymia” OR “dysthymic”) AND (“MBCT” OR "mindfulness-based cognitive therapy") AND (“randomized controlled trial” OR “randomised controlled trial” OR “randomized-controlled trial” OR “randomised-controlled trial” OR “RCT” OR “randomized” or “randomised” OR “randomly allocated” OR “randomly assigned”)
PubMed	(“MDE” OR “major depressive episode” OR “MDD” OR “major depressive disorder” OR “major depression” OR “depression” OR “dysthymia” OR “dysthymic”) AND (“chronic” OR “persistent” OR “treatment-resistant” OR “non-responders” OR “non-response” OR “non-remitted” OR “dysthymia” OR “dysthymic”) AND (“MBCT” OR "mindfulness-based cognitive therapy") AND (“randomized controlled trial” OR “randomised controlled trial” OR “randomized-controlled trial” OR “randomised-controlled trial” OR “RCT” OR “randomized” or “randomised” OR “randomly allocated” OR “randomly assigned”)
PsycInfo	(“MDE” OR “major depressive episode” OR “MDD” OR “major depressive disorder” OR “major depression” OR “depression” OR “dysthymia” OR “dysthymic”) **AND** (“chronic” OR “persistent” OR “treatment-resistant” OR “non-responders” OR “non-response” OR “non-remitted” OR “dysthymia” OR “dysthymic”) **AND** (“MBCT” OR "mindfulness-based cognitive therapy") **AND** (“randomized controlled trial” OR “randomised controlled trial” OR “randomized-controlled trial” OR “randomised-controlled trial” OR “RCT” OR “randomized” or “randomised” OR “randomly allocated” OR “randomly assigned”)
Embase	(‘MDE’ OR ‘major depressive episode’ OR ‘MDD’ OR ‘major depressive disorder’ OR ‘major depression’ OR ‘depression’ OR ‘dysthymia’ OR ‘dysthymic’) **AND** (‘chronic’ OR ‘persistent’ OR ‘treatment-resistant’ OR ‘non-responders’ OR ‘non-response’ OR ‘non-remitted’ OR ‘dysthymia’ OR ‘dysthymic’) **AND** (‘MBCT’ OR ‘mindfulness-based cognitive therapy’) **AND** (‘randomized controlled trial’ OR ‘randomised controlled trial’ OR ‘randomized-controlled trial’ OR ‘randomised-controlled trial’ OR ‘RCT’ OR ‘randomized’ or ‘randomised’ OR ‘randomly allocated’ OR ‘randomly assigned’)

For peer review only - http://bmjopen.bmj.com/site/about/guidelines.xhtml

Cochrane Controlled Trials	(“MDE” OR “major depressive episode” OR “MDD” OR “major depressive disorder” OR “major depression” OR “depression” OR “dysthymia” OR “dysthymic”) AND (“chronic” OR “persistent” OR “treatment-resistant” OR “non-responders” OR “non-response” OR “non-remitted” OR “dysthymia” OR “dysthymic”) AND (“MBCT” OR "mindfulness-based cognitive therapy") AND (“randomized controlled trial” OR “randomised controlled trial” OR “randomized-controlled trial” OR “randomised-controlled trial” OR “RCT” OR “randomized” or “randomised” OR “randomly allocated” OR “randomly assigned”)

For peer review only

BMJ Open

Efficacy and Moderators of Mindfulness-Based Cognitive Therapy (MBCT) in 'Difficult to Treat' Depression: Protocol for a Systematic Review and Individual Patient Data Meta-Analysis of Randomised Controlled Trials

Journal:	*BMJ Open*
Manuscript ID	bmjopen-2025-106350.R4
Article Type:	Protocol
Date Submitted by the Author:	14-Nov-2025
Complete List of Authors:	Barnhofer, Thorsten; University of Surrey,<br>Niemi, Maria; Karolinska Institutet, Department of Global Public Health<br>Michalak, Johannes; Witten/Herdecke University, Department of Psychology and Psychotherapy<br>Velana, Maria; Witten/Herdecke University, Department of Psychology and Psychotherapy<br>Williams, J Mark; University of Oxford, Department of Psychiatry<br>Chiesa, Alberto; Università di Bologna<br>Eisendrath, Stuart; University of California at San Francisco<br>Delucchi, Kevin; University of California at San Francisco<br>Segal, Zindel; University of Toronto - Scarborough, Graduate Department of Psychological Clinical Science<br>Cladder-Micus, Mira; Radboud University Nijmegen<br>Speckens, Anne; Radboudumc Instituut voor Wetenschappelijk Onderwijs en Opleidingen, Psychiatry<br>Foroughi, Ali Akbar; Kermanshah University of Medical Sciences (KUMS), Department of Clinical Psychology<br>García-Toro, M; Research Network on Chronicity Primary Care and Health Promotion; University of the Balearic Islands; Health Research Institute of the Balearic Islands; University of the Balearic Islands, Department of Medicine<br>Montero-Marin, Jesus; Research & Innovation Unit Parc Sanitari Sant Joan de Déu; University of Oxford, Department of Psychiatry; Consortium for Biomedical Research in Epidemiology and Public Health<br>dunn, barney; University of Exeter<br>Strauss, Clara; Sussex Partnership NHS Foundation Trust; University of Sussex<br>Ruths, Florian; South London and Maudsley NHS Foundation Trust<br>Ryan, Mary; Royal College of Psychiatrists<br>Harrer, Mathias; Technical University of Munich, Department of Psychiatry and Psychotherapy; World Health Organization Collaborating Center for Research and Dissemination of Psychological Interventions, Department of Clinical, Neuro and Developmental Psychology; Vrije Universiteit
**Primary Subject Heading**:	Mental health
Secondary Subject Heading:	Mental health

Keywords:	Meta-Analysis, Depression & mood disorders < PSYCHIATRY, Mindfulness

SCHOLARONE™
Manuscripts

Title

Efficacy and Moderators of Mindfulness-Based Cognitive Therapy (MBCT) in 'Difficult to Treat' Depression: Protocol for a Systematic Review and Individual Patient Data Meta-Analysis of Randomised Controlled Trials

Running Title

MBCT in 'Difficult to Treat' Depression

Authors and Affiliations

Thorsten Barnhofer [1], Maria Niemi [2, *], Johannes Michalak [3], Maria Velana [3], J. Mark G. Williams [4], Alberto Chiesa [5], Stuart Eisendrath [6], Kevin Delucchi [6], Zindel V. Segal [7], Mira Cladder-Micus [8], Anne Speckens [9], Ali Akbar Foroughi [10], Mauro Garcia-Toro [11, 12, 13, 14], Jesus Montero-Marin [15, 4, 16], Barnaby D. Dunn [17], Clara Strauss [18, 19], Florian A. Ruths [20], Mary Ryan [21], Mathias Harrer [22, 23]

[1] School of Psychology, University of Surrey, Guildford, UK, t.barnhofer@surrey.ac.uk

[2] Department of Global Public Health, Karolinska Institutet, Stockholm, Sweden, maria.niemi@ki.se

[3] Department of Psychology and Psychotherapy, Witten/Herdecke University, Witten, Germany, Maria.Velana@uni-wh.de , Johannes.Michalak@uni-wh.de

[4] Department of Psychiatry, University of Oxford, Oxford, UK, catherine.crane@psych.ox.ac.uk, mark.williams@psych.ox.ac.uk, jesus.monteromarin@psych.ox.ac.uk

[5] Institute of Psychiatry, Università di Bologna, Bologna, Italy, chiesa@scuola-spc.it

[6] Weill Institute for Neurosciences, University of California at San Francisco, San Francisco, USA, stuart.eisendrath@ucsf.edu, kevin.delucchi@ucsf.edu

For peer review only - http://bmjopen.bmj.com/site/about/guidelines.xhtml

[7] Graduate Department of Psychological Clinical Science, University of Toronto - Scarborough, Toronto, Canada, zindel.segal@utoronto.ca

[8] Behavioural Science Institute, Radboud University Nijmegen, Nijmegen, The Netherlands, Mira.Cladder-Micus@radboudumc.nl

[9] Department of Psychiatry, Radboudumc, Nijmegen, The Netherlands, anne.speckens@radboudumc.nl

[10] Department of Clinical Psychology, Kermanshah University of Medical Sciences (KUMS), Hermanshah, Iran, foroughi_2002@yahoo.com

[11] Research Network on Chronicity, Primary Care and Health Promotion (RICAPPS), Carlos III Health Institute, Madrid, Spain

[12] University Institute of Health Science Research (IUNICS), University of the Balearic Islands, Spain, mauro.garcia@uib.es

[13] Health Research Institute of the Balearic Islands (IdISBa), Palma, Spain

[14] Department of Medicine, University of the Balearic Islands, Palma, Spain

[15] Research & Innovation Unit, Parc Sanitari Sant Joan de Déu, Sant Boi de Llobregat, Barcelona, Spain

[16] Consortium for Biomedical Research in Epidemiology and Public Health (CIBER Epidemiology and Public Health-CIBERESP), Madrid, Spain

[17] Mood Disorders Centre, University of Exeter, Exeter, UK, b.d.dunn@exeter.ac.uk

[18] Sussex Partnership NHS Foundation Trust, Hove, UK, clara.strauss@nhs.net

[19] Sussex Mental Health Research Centre, University of Sussex, Brighton, UK

[20] South London and Maudsley NHS Foundation Trust, London, UK, Florian.Ruths@slam.nhs.uk

[21] Royal College of Psychiatrists, London, UK, mary@highbag.co.uk

For peer review only

[22] Department of Psychiatry and Psychotherapy, School of Medicine and Health, Technical University of Munich, Munich, Germany

[23] Department of Clinical, Neuro and Developmental Psychology, World Health Organization Collaborating Center for Research and Dissemination of Psychological Interventions, Amsterdam, Public Health Research Institute, Vrije Universiteit, Amsterdam, The Netherlands, mathias.harrer@tum.de

* Corresponding author:

Associate Professor Maria Niemi, Department of Global Public Health, Karolinska Institutet, Stockholm, Sweden, maria.niemi@ki.se

For peer review only

Word Count

Abstract: 300

Main text: 4110

Keywords

Major Depression, Difficult-to-Treat, Mindfulness-Based Cognitive Therapy, Independent Patient Data, Meta-Analysis

For peer review only - http://bmjopen.bmj.com/site/about/guidelines.xhtml

ABSTRACT

Introduction: About 30% of depressed patients suffer from a protracted course in which the disorder continues to cause significant burden despite treatment efforts. While originally developed for relapse prevention, Mindfulness-Based Cognitive Therapy (MBCT) has increasingly been investigated in depressed patients with such 'difficult-to-treat' courses. This is a protocol for an individual participant data (IPD) meta-analysis aiming to determine efficacy and potential moderators of MBCT treatment effects in this group based on evidence from randomised controlled trials.

Methods and analysis: Systematic searches in PubMed, Web of Science, Scopus, PsycINFO, EMBASE, and the Cochrane Controlled Trials Register for randomised controlled trials were completed on 17 June 2024. Authors of identified studies have contributed IPD and data extractions have been completed. An update search will be conducted immediately before the start of data analyses. We will investigate the following outcomes: (a) self-reported and observer-reported severity of depression symptomatology, (b) remission, (c) clinically meaningful improvement and deterioration. One-stage and two-stage IPD meta-analyses will be conducted with one-stage models using the observed IPD from all studies simultaneously as the primary approach. One-stage IPD models will include stratified study intercepts and error terms as well as random effects to capture between-study heterogeneity. Moderator analyses will test treatment-covariate interactions for both individual patient level and study level characteristics.

Ethics and dissemination: The results will inform understanding of the use of MBCT in patients with current 'difficult-to-treat' depression and will contribute to arguments in favour of or against implementing MBCT as a treatment for this group. They will be published in a peer reviewed journal and made available to stakeholders in accessible formats. No local ethical review was necessary following consultation with the Ethics and Governance Board

For peer review only - http://bmjopen.bmj.com/site/about/guidelines.xhtml

of the University of Surrey. Guidance on patient data storage and management will be adhered to throughout.

PROSPERO registration number: CRD42022332039

For peer review only

For peer review only - http://bmjopen.bmj.com/site/about/guidelines.xhtml

STRENGTHS AND LIMITATIONS OF THIS STUDY

- The chosen approach using IPD has several advantages over conventional meta-analyses including the possibility to assess whether individual participant level characteristics influence treatment effects with more specificity and power
- Limitations of the approach include the fact that inconsistencies across studies regarding the format and type of variables reported may restrict our ability to investigate moderators of treatment effects
- Transformation of outcome measures to a common metric across all studies may come with a small loss of information.

For peer review only

For peer review only - http://bmjopen.bmj.com/site/about/guidelines.xhtml

INTRODUCTION

Major Depressive Disorder (MDD) is highly prevalent, debilitating [1] , and in about 30% of patients takes a recurrent or chronic course in which established treatments fail to bring sustained remission [2] . Treatment resistance has traditionally been defined in narrow operational terms (e.g. non-response after two adequate pharmacological treatments), but such definitions vary across studies and exclude many patients with persistent, impairing depression. The broader construct of difficult-to-treat depression (DTD) was recently introduced to capture the true spectrum within a single heuristic, providing a clinically meaningful framework that better reflects the overlap between these groups and the challenges faced in practice [3, 4]. Defined as "depression that continues to cause significant burden despite usual treatment efforts", such courses are associated with continuing functional impairment, reduced quality of life, and significantly increased risk for chronic physical and neurodegenerative disorders [5, 6] . Finding effective treatments for DTD remains an important challenge for depression research [7] .

While treatment approaches for DTD have traditionally been dominated by pharmacological strategies [8] , it is now increasingly acknowledged that effective management requires a broader emphasis on working with symptoms, maximising function and minimizing burden [4] . Psychological interventions have an important contribution to make in this context [9] and emerging evidence suggests that Mindfulness-Based Cognitive Therapy (MBCT [10]), an eight-week treatment that combines elements of cognitive therapy with mindfulness meditation, may be suited for this purpose [11] . Using a mental training approach, the intervention aims to provide patients with portable skills that enable them to disengage from habitual maladaptive responses and respond more adaptively to negative mood and stress.

For peer review only - http://bmjopen.bmj.com/site/about/guidelines.xhtml

MBCT was originally introduced for the prevention of relapse in patients who are currently in remission and has been proven to be effective for this purpose [12] , particularly in patients with highly recurrent courses (a use that is endorsed by treatment guidelines internationally). Early trials restricted the intervention to patients who were currently in full remission as it was assumed that intensive mindfulness practice may be too demanding for those who are suffering from current symptoms [13, 14] . However, as patients who are at high risk of relapse are likely to show residual symptoms between episodes, this restriction was later loosened, and analyses across studies for relapse prevention show that treatment effects increase, rather than decrease, with level of residual symptoms [12] . From a theoretical perspective, this observation is in line with the view that mindfulness skills serve to buffer the effects of negative mood and stress [15, 16] , which implies that effects should become more visible under conditions of higher symptoms or stress.

Based on this reasoning, research has extended the use of MBCT to patients with current depression described as 'difficult-to-treat'. Preliminary studies investigating MBCT in treatment-resistant and chronic depression were published in 2008 [17] and 2009 [18] and several definitive randomised controlled trials have been conducted since then [19–24] , suggesting that evidence has accumulated to a point where the use of MBCT as a treatment for patients with current DTD can now be considered for guideline endorsement and wider implementation. However, conclusions from individual studies are contrained by heterogeneous definitions and settings, variable comparators and follow-up durations, inconsistent outcome reporting, and limited power for moderator analyses. It seems timely therefore to bring data from existing randomised controlled trials together and analyse outcome across studies.

For this purpose, we have pooled individual patient data (IPD) from these studies to conduct meta-analyses with one-stage random effects meta-analysis of independent patient

For peer review only

data (IPD) as our primary approach given its advantages over conventional aggregate data meta-analyses [25–28] . This IPD meta-analysis will apply a consistent DTD framework, harmonise outcomes, estimate overall effects using one-stage models, and test prespecified patient-level moderators, with comprehensive risk-of-bias and small-study assessments. The main aim of the analyses is to establish efficacy of MBCT for patients with DTD as compared to the control conditions utilised in the existing trials. Given the need for both effective symptom reduction and the maintenance of gains, we will aim to determine immediate effects at the end of the eight-week MBCT intervention (post-treatment) and effects in the longer-term after patients have finished the intervention and are free to continue engaging in mindfulness practice by themselves (follow-up).

An important advantage of IPD meta-analysis is that it allows to examine moderator or subgroup questions across the pooled sample and can thus serve to overcome limitations in sample size and power for answering these questions in the original studies [29] . Information about potential moderators of treatment effects, such as severity of depression at entry to treatment or a history of bipolar disorder, is important as it can potentially help with implementation by providing more precise information about relative indications or contraindications of the intervention. A further aim of this IPD meta-analysis is therefore to explore effects of potential individual patient level (e.g. sociodemographic and clinical characteristics) and study level (e.g. delivery format) moderators of treatment effects in the pooled data set.

As the clinical concept of DTD does not link to an objective way of assessment, the current meta-analysis will include studies that have used a range of conventional definitions falling under the wider concept of DTD, including selecting participants based on treatment non-response/remission, treatment resistance or the presence of chronic depression. Subgroup analyses will allow us to determine the extent to which effects found in the aggregated data

For peer review only

generalise across these conventional categories. Given that DTD reflects a wider construct encompassing a large and heterogeneous group of patients with different characteristics and different treatment histories, we will aim to carefully describe the sample both on the meta- and the study-level.

METHODS

General approach

This IPD meta-analysis is registered as part of a broader project on PROSPERO (registration number: CRD42022332039; date of registration in PROSPERO: 24 May 2022) and any key changes or amendments will be documented there. In addition to the direct comparisons between MBCT and control conditions, the wider project also includes studies of an individual psychotherapeutic intervention, Cognitive Behavioral Analysis System of Psychotherapy, and considers a wider range of outcomes, including both beneficial and adverse treatment effects. The current protocol specifies procedures for the comparison between MBCT and the control conditions utilized in the existing MBCT trials only as we assume that this comparison is of particular interest given the need for establishing further treatment options for DTD. The moderator and sensitivity analyses described here were prespecified in this protocol prior to data analysis but were not detailed in the initial PROSPERO entry. They are included to address theoretically and clinically relevant questions, and all analyses (including null results) will be reported to minimise selective reporting. The protocol is registered retrospectively as searches to identify eligible papers were already conducted in 2024, with the last search having been performed on 17 June 2024 and an update search planned immediately before the start of data analysis. Corresponding authors of the selected studies were contacted and asked to provide raw data from their studies. The Preferred Reporting Items for Systematic Reviews and Meta-Analyses IPD statement [30] will be followed for the reporting of this study.

For peer review only - http://bmjopen.bmj.com/site/about/guidelines.xhtml

Eligibility criteria

Types of studies to be included

This IPD meta-analysis will include only peer-reviewed RCTs, published in English. There will be no restriction on the type of recruitment settings. The restriction to English-language publications is due to feasibility constraints and is recognised as a potential source of language bias.

Type of intervention

Included studies will have investigated the effects of manualised mindfulness-based cognitive therapy (MBCT) alone or as an adjunct treatment to treatment-as-usual (TAU) [31]. MBCT is a psychological group-based intervention that combines intensive training in mindfulness meditation with elements of cognitive behavioural therapy (CBT) for depression. Mindfulness can be operationally defined as non-judgemental awareness of present moment experience [32]. As MBCT was originally designed to prevent relapse rather than to reduce symptoms during a current episode, we will accept studies that introduce minor adaptations to the *delivery* of the manualised approach (e.g. incorporating specific support for managing current depressive symptoms, particularly around the engagement in regular practice, and rephrasing psycho-educational components to address current depression rather than relapse, *without altering the structure or core content of the manual*). Other types of mindfulness-based interventions will not be included. MBCT studies will be considered regardless of delivery format (e.g. physical group setting versus videoconference).

Types of comparators

This may include TAU for depression, including pharmacological and psychological treatments (e.g., CBT) or active controls. The latter will include established interventions as well as control interventions designed to mimic effects of mindfulness-based interventions in some or all aspects with the exception of the core element of mindfulness.

For peer review only - http://bmjopen.bmj.com/site/about/guidelines.xhtml

Participants

We will include studies that recruited adults with current Major Depressive Disorder (MDD) [33, 34] and studies which included mixed groups of patients with residual depressive symptoms or full episodes in the context of a chronic or treatment-resistant course of the disorder. In the latter case, participants with only residual symptoms will be excluded from the primary analysis approach (one-stage meta-analysis). As DTD does not link to an objective way of assessment, we will opt for a maximally inclusive interpretation of the term and include any studies that selected patients based on previous treatment non-response/remission, treatment-resistance or chronicity. We will exclude studies with participants below the age of 18 and studies that have exclusively recruited participants over the age of 65 given that the factors involved in the onset, maintenance and recurrence of depression in these age groups can differ from those in adults within this age bracket [35, 36] .

Outcomes

The main type of outcome will be severity of depressive symptomatology as assessed using standard depression outcome measures. Standard depression outcome measures may include self-report measures and observer-rated measures. If both types of measures are available, we will investigate effects on both types of outcome measures. If several measures are available for a single study, preference will be given to the measure that was used as the primary outcome of the original study. To compare outcomes across studies using different outcome measures, we will transform scores from these measures into standardised scores using the common metric developed by Wahl et al. [37] . Where a measure is not part of the common metric approach, we will transform the measure into one that is part of the common metric using published conversion tables [38] , and then derive the common metric.

For peer review only - http://bmjopen.bmj.com/site/about/guidelines.xhtml

To facilitate clinical interpretation of results, we will derive dichotomous outcomes reflecting remission, improvement and deterioration. To apply a common rule for all studies, common metric scores will be converted to the PHQ-9 and dichotomous outcomes computed based on this measure. Following conventions for the PHQ-9 used in pragmatic trials and health services, remission will be defined as a score of less than 10 [39, 40], i.e. a shift from "caseness" to a non-case score. Definition of clinically meaningful change will follow suggestions from recent trials in treatment-resistant depression, which has indicated a threshold of 6 or more points [41, 42] . We will therefore define clinically meaningful improvement as a reduction of 6 or more points and clinically meaningful deterioration as an increase of 6 or more points on the PHQ-9.

A more detailed analysis of negative effects including suicidality is covered in a separate protocol covering a network meta-analysis of MBCT and another individual psychotherapeutic intervention (Michalak et al., in submission). Secondary outcomes such as mindfulness, self-compassion and quality of life will be collected and may be used for future analysis.

Moderators

We will also investigate potential moderators of treatment response, i.e. factors that determine whether a person responds better to MBCT as compared to the control condition or vice versa. These will include potential moderators on the individual patient level (e.g. sociodemographic characteristics, history of childhood trauma, and clinical characteristics) and on the study level. We will include patient level characteristics in the analyses, if they are reported across a sufficient number of studies and datasets (see statistical analysis section for criteria).

Sociodemographic and clinical characteristics that we aim to investigate include age [43] , gender [44] , marital status [45] , education [46] , employment [47] , childhood

For peer review only - http://bmjopen.bmj.com/site/about/guidelines.xhtml

adversity [48] , age at onset of depression [49] , number of previous episodes [50] , duration of current episode [51] , current suicidality [52] , current antidepressants [53] , past antidepressants and treatment-resistance to antidepressants [54] , co-morbid anxiety disorder, comorbid obsessive-compulsive disorder, co-morbid post-traumatic stress disorder, co-morbid substance abuse or dependence, co-morbid eating disorder, co-morbid personality disorder [43], and level of depressive symptoms at entry [55] . Study level characteristics will include type of delivery (remote versus face-to-face), type of control condition (TAU, active control), type of DTD (non-response/remission, treatment-resistance, chronic depression) and study quality.

Timing of outcome assessments

As follow-up periods of the studies differ, we will group follow-up periods in the following categories: 8 to 10 weeks post-randomisation (post-treatment), 11 to 34 weeks post-randomisation (follow-up 1), and 35 to 60 weeks post-randomisation (follow-up 2).

Searches and study selection

We searched PubMed, Web of Science, Scopus, PsychINFO, EMBASE, and the Cochrane Controlled Trials Register using index and free terms, jointly with Boolean operators, on four tiers, namely: (1) depressive disorder, (2) non-response, treatment resistance and chronicity, (3) mindfulness-based cognitive therapy, and (4) randomised-controlled trial. The search terms used for the different databases are shown in the appendix. We did not search grey literature or trial registries beyond the Cochrane Controlled Trials Register, and we did not consult experts outside of the research team.

We searched for any study published up until the day of the search, on 17 June 2024. There was no lower time limit. Initial screening of titles was performed by two independent reviewers (TB, JM), with the publication abstract being obtained if selected by at least one reviewer. Abstracts were screened by the same two independent reviewers, with

For peer review only - http://bmjopen.bmj.com/site/about/guidelines.xhtml

disagreements about eligibility being referred to a third reviewer whose decision was final. An update search will be conducted immediately before the start of data analysis.

Risk of bias assessment

The validity of the studies will be assessed using the Risk of Bias tool by the Cochrane Collaboration (version 2) [56, 57] . Possible sources of bias assessed by the tool are (1) random sequence generation, (2) allocation concealment, (3) blinding of outcome assessors, (4) incomplete outcome data, (5) selective outcome reporting, and (6) other threats to validity including lack of compliance and different timing of outcome assessments. Data from the published papers of the trials will be evaluated by two independent assessors. Agreement rate will be reported, and any disagreements will be solved through discussion. A sensitivity analysis will be performed excluding studies judged to be at high risk of bias, or where risk of bias is unclear.

Patient and public involvement

A lived experience advisory group, led by MR and consisting of experts who have been involved in previous trials of MBCT, will be consulted for the interpretation of study results and their dissemination. A coproduced lay summary of results will be written for dissemination alongside papers for peer review journals.

IPD data collection and aggregation

All corresponding authors of the articles selected for inclusion were contacted via email by TB with an invitation to participate in the review and to share the IPD from their primary study. The standard letter described all relevant details of the study including its purpose, main research questions, and the variables of interest and ended with a request to share the raw data from the study. Corresponding authors of studies were invited to serve as collaborators for the project. If corresponding authors did not respond within four weeks, we sent a second email request. If this had failed, a second author was contacted. Apart from one

For peer review only

For peer review only - http://bmjopen.bmj.com/site/about/guidelines.xhtml

study, whose authors could not be contacted, none of the selected studies required contact attempts beyond a second author and the responding authors agreed to share their data.

Data checking and integrity

Authors were requested to provide a codebook or data dictionary explaining variable names, coding of values, measurement units etc. After accepting the invitation to collaborate and signing the data transfer agreement, the study authors shared their IPD via secure data transfer portal at the University of Surrey. Data is stored securely on University of Surrey servers in accordance with the European Union's General Data Protection Regulation Act and the UK's Data Protection Act 2018.

TB and MH will take primary responsibility for preparing and extracting outcome data and other characteristics, with MV independently checking extractions for accuracy. Received datasets will be reviewed to assess completeness and checked against the reported data from relevant study publications. The numbers of participants, overall and by arm, will be confirmed to correspond with the numbers in the study publication. Ranges of values will be inspected for clearly erroneous data. Baseline participant demographic data will be inspected by arm, with the aim of checking that the IPD matches the data reported in the publication, in terms of the numbers of participants with reported data, and overall aggregated values for each variable. Discrepancies will be referred to the study authors for investigation and any remaining discrepancies will be recorded. For each included trial, we will extract information on funding sources and any reported conflicts of interest, and this information will be presented alongside other study characteristics.

Although the main search and initial receipt of IPD occurred before the submission of this protocol for publication, the protocol was registered in PROSPERO (CRD42022332039) beforehand. No data analysis or outcome-driven decisions were made prior to finalizing the protocol, and the methods described here were pre-specified prior to data inspection.

For peer review only - http://bmjopen.bmj.com/site/about/guidelines.xhtml

Data harmonization

Once datasets from the individual studies have been checked and outcomes standardised, they will be merged into the final dataset. The final dataset for analysis will be checked again for accuracy by a researcher in the study team.

STATISTICAL ANALYSIS

To finalise the selection of moderator variables, we will determine presence of these variables across the individual data sets and decide on ways to standardise these variables where needed, for example by collapsing categories. Potential moderator variables will be included in the analyses if the variable has at least 40% available data in the final dataset and has been recorded by at least 4 studies.

To investigate the efficacy of MBCT compared to control conditions and test moderating effects of individual patient level and study level characteristics, we will conduct one-stage and two-stage IPD meta-analyses (IPD-MAs). Random-effects models will be used throughout because we expect high statistical heterogeneity due to variation in control groups (treatment as usual, active controls) and study populations (treatment non-responders/remitters, treatment-resistance, chronic depression).

The main research question of the study (whether MBCT is efficacious compared to control conditions) will be addressed via a maximum of three pairwise comparisons: (1) MBCT versus treatment-as-usual controls, (2) MBCT versus active controls, and (3) MBCT versus all controls.

By default, all analyses will be conducted in a Bayesian framework using JAGS (Just Another Gibbs Sampler)/BUGS (Bayesian analysis Using Gibbs Sampling) [58]. We chose a Bayesian approach because it offers greater flexibility in the parametrization of the (one-stage) IPD-MA models, and because this allows for a more intuitive interpretation of results

For peer review only - http://bmjopen.bmj.com/site/about/guidelines.xhtml

based on posterior tail probabilities [59] . Across all analyses, findings will be considered "significant" when the credibility interval of the relevant effect does not include zero.

Two-stage random effects meta-analysis

We will first conduct a conventional meta-analysis using data extracted from the published papers. We will calculate Hedges' g to determine the effect sizes of the difference between the intervention and the control conditions reported in the papers. To investigate heterogeneity of effect sizes, we will calculate I^2, the percentage of variation not attributable to sampling error, as well as 95% prediction intervals (PIs) around the pooled effect [60, 61] . Leave-one-out analyses will be conducted to identify potential outliers and/or influential studies. Small-study effects will be investigated visually using funnel plot inspection and the Egger's test for asymmetry. A weakly informative Half-Cauchy (0,0.5) prior will be used for the between-study heterogeneity variance τ^2 [62], and flat normal priors for all other parameters. A detailed justification for this prior setup has been derived in previous work [63].

One-stage random effects meta-analysis using IPD

One-stage IPD meta-analysis models will be used as the primary analytic approach. To analyse the effects on common metrics-converted symptom severity scores, an identity-link linear mixed effects model with stratified intercepts and trial-specific (heteroscedastic) error terms will be used. Let y_{ik} be the depressive symptom severity of patient i in trial k (with k=1, …, K studies included in the meta-analysis). The one-stage IPD can then be defined as:

$$y_{ik} = \alpha_k + \theta T_{ik} + u_k T_{ik} + \beta_k \left(X_{ik} - \overline{X}_{ik}\right) + \varepsilon_{ik} \qquad (1)$$

$$u_k \sim \mathcal{N}\left(0,\tau_\theta^2\right) \qquad \varepsilon_{ik} \sim \mathcal{N}\left(0,\sigma_k^2\right)$$

Where T_{ik} is the treatment indicator, X_{ik} is the symptom severity score at baseline, $\overline{X}_{ik}$ is the mean of X for each specific trial, α_k is the stratified trial intercept, and u_k is the

For peer review only - http://bmjopen.bmj.com/site/about/guidelines.xhtml
For peer review only

trial-specific random slope of the average treatment effect θ. For this model, priors mirroring the two-stage approach will be used throughout, with a uniform distribution assigned to σ_k^2.

The same model specification, but with a logit link, will be used to model the effects on binary outcomes, including remission, clinically meaningful improvement and clinically meaningful deterioration based on PHQ-9 thresholds:

$$\log_e\left(\frac{\pi_{ik}}{1-\pi_{ik}}\right) = \alpha_k + \theta T_{ik} + u_k T_{ik} + \beta_k\left(X_{ik} - \bar{X}_{ik}\right) + \varepsilon_{ik} \\ y_{ik} \sim \mathrm{Bern}(\pi_{ik}) \quad u_k \sim \mathcal{N}\left(0,\tau_\theta^2\right) \quad \varepsilon_{ik} \sim \mathcal{N}\left(0,\sigma_k^2\right) \tag{2}$$

Where y_{ik} now represents the patient's binary outcome of interest. Numbers needed to treat (*NNT*) corresponding to the estimated SMD values will be calculated using the method by Furukawa and Leucht [64], and as the inverse of the absolute risk reduction otherwise.

Moderator analyses

We will explore potential moderators of treatment effect by adding individual participant level and study level treatment-covariate interactions into the main IPD-MA model. To optimise power, these analyses will be done on treatment effects at post-treatment as all studies can provide data for this timepoint.

Missing data

We will record the percentage of individual participant missing data for baseline characteristics and outcomes in each of the studies included in the meta-analyses. All analyses will be conducted according to the "intention-to-treat" principle, so that analyses target a treatment policy estimand [65]. Missing data will be handled using multiple imputation (fully conditional specification; MICE algorithm [66] under the missing at random (MAR) assumption. For the main effectiveness analysis, multilevel two-stage imputation models with heteroscedastic errors will be used to account for the nested data

For peer review only - http://bmjopen.bmj.com/site/about/guidelines.xhtml

structure [67]. Highly collinear variables will be removed as predictors, as well as variables with systematically missing information (“structural zeros”). A total of m=50 imputation sets will be generated. A separate imputation model will be constructed for each focal moderator, including auxiliary variables with a maximum of 10% missingness. Imputations will be generated separately for each of the treatment groups (intervention, control) to account for treatment-covariate interactions. All analysis models will be fitted in the multiply imputed data. Parameter estimates and test statistics will then be aggregated by mixing the draws from the posterior distribution of each completed dataset [68]. Sensitivity analyses will be performed to compare results using observed and imputed data [69] .

Subgroup analyses

We plan to examine the efficacy of MBCT and potential moderators of treatment outcomes in subgroups defined by the conventional descriptive categories used by the individual studies to define their populations, distinguishing between studies that recruited patients characterised as treatment non-responders/remitters, treatment-resistant, or as suffering from chronic depression. These analyses will be based on subsamples created from the final IPD dataset.

Sensitivity analyses

As a sensitivity analysis, we will repeat all main effectiveness analyses using equivalent frequentist (one- and two-stage) approaches. Additionally, we will conduct sensitivity analyses to investigate whether sources of heterogeneity affect the overall effect size estimate and robustness of our findings. This will include exploring the role of risk of bias and other potential study level sources of heterogeneity that may become obvious after data have been examined. These analyses will be conducted using a two-stage IPD-MA model.

DISCUSSION

For peer review only - http://bmjopen.bmj.com/site/about/guidelines.xhtml
For peer review only

This IPD meta-analysis will provide estimates of efficacy for the use of MBCT in DTD and offer comprehensive information about potential moderators of treatment effects. As such it will contribute to arguments in favour of or against implementing MBCT as a treatment for DTD and may help to decide whether MBCT is a recommendable treatment option for this group. This is important given the need to improve the care and management of DTD and the increasing recognition that psychological therapies can have an integral role in this endeavour. Information about potential moderators of treatment effects will help to refine the understanding of who may or may not benefit from MBCT for DTD.

We have chosen the emerging concept of DTD as an umbrella because it provides a broad conceptualisation of depression that remains burdensome despite usual treatment attempts. Most available trials have operationalised this construct using narrower criteria such as non-response, non-remission, treatment-resistance, or chronicity, and our inclusion criteria therefore reflect how DTD has been studied to date. These characteristics represent core manifestations of DTD, and their use ensures consistency and feasibility for the planned meta-analysis. We acknowledge, however, that DTD extends beyond these dimensions, for example in its emphasis on functional outcomes, and generalisability of findings to the broader construct should be interpreted with this in mind.

The chosen approach using IPD has several advantages over conventional meta-analyses including the possibility to assess whether individual participant level characteristics influence treatment effects with more specificity and power. Limitations of the approach include the fact that inconsistencies across studies regarding the format and type of variables reported may restrict our ability to investigate moderators of treatment effects and that transformation of outcome measures to a common metric across all studies may come with a small loss of information. To address this latter point, the current study will complement one-stage IPD analyses with a conventional two-stage approach. A more general concern that is

For peer review only - http://bmjopen.bmj.com/site/about/guidelines.xhtml

often raised for IPD meta-analyses is inclusion bias as it may be difficult to obtain IPD for all selected studies.

This IPD meta-analysis is registered as part of a broader project that examines a wider range of interventions and outcomes, including both beneficial and adverse effects. The present protocol specifies procedures for the comparison between MBCT and control conditions in MBCT trials, as we assume that this comparison is of particular interest given the need to establish interventions for DTD. To our knowledge, this is the first IPD meta-analysis to investigate the effects of MBCT in DTD and assess moderators of treatment effects. As evidence from trials of MBCT for DTD has now accumulated to a point where the use of MBCT for DTD can be considered for endorsement in treatment guidelines, findings from this IPD meta-analysis have the potential to inform treatment recommendations and decisions regarding implementation. The results will provide clinicians, healthcare providers and people who are suffering from DTD with information on the characteristics that may make it more or less likely for patients to benefit from this treatment and thus facilitate implementation.

ETHICS AND DISSEMINATION

No local ethical review was necessary following consultation with the Ethics and Governance Board of the University of Surrey. The investigators of the primary trials have local ethical approval for sharing the data. TB and MH will be responsible for oversight of the database and analyses will be conducted by MH and TB. Patient privacy will be ensured by adhering to University of Surrey guidance on data management and storage. Exchange of data is governed by inter-institutional data sharing agreements. All data were pseudonymised (de-identified) before sharing, shared through secure upload facilities and are stored on secure servers. The data set will not be open access. Researchers of good standing may receive access pending on institutional approvals on data transfer.

For peer review only - http://bmjopen.bmj.com/site/about/guidelines.xhtml

We will disseminate the work in peer-reviewed journals, provide lay summaries and discuss findings with patient and lived experience groups. Other means of communication to disseminate the results will include conference presentations and social media announcements.

CONTRIBUTIONS

TB, MN, JM, MV and MH conceptualised and designed the study. TB developed the search strategy and contacted the primary authors, who contributed to further refinement of the design and approach of the study. TB, MV and MH are responsible for building the database. MH provides statistical expertise and is responsible for data analyses. TB drafted the protocol manuscript with statistical input by MH, which was critically revised by all authors. All authors read and approved the final version. TB serves as the guarantor of the review.

FUNDING STATEMENT

This research received no specific grant from any funding agency in the public, commercial or not-for-profit sectors.

ACKNOWLEDGMENTS

JMM has a ‘Miguel Servet’ research contract from the ISCIII (CP21/00080), and he is grateful to the Spanish CIBER of Epidemiology and Public Health (CBERESP CB22/02/00052< ISCIII) for their support.

COMPETING INTERESTS STATEMENT

TB is the co-author of a book on mindfulness-based interventions and regularly offers workshops on mindfulness-based interventions. He is the co-investigator of a programme grant evaluating an adapted MBCT course for adolescents experiencing depression.

MN is author of a book chapter on mindfulness-based interventions and offers workshops on mindfulness-based interventions.

For peer review only - http://bmjopen.bmj.com/site/about/guidelines.xhtml

JM is the director of the Achtsamkeitsinstitut Ruhr (an institute offering mindfulness training) and principal investigator of several DFG (German Science Foundation) research projects. He receives royalties from mindfulness books he has authored.

MV reports no conflicts of interest.

JMGW co-developed MBCT, and was founding Director of the University of Oxford Mindfulness Centre. He receives royalties from books describing the MBCT program. He also receives honoraria from lectures and training workshops on mindfulness.

AC leads a professional practice (Istituto Mente Corpo) that offers courses of mindfulness-based interventions.

SE is the author of book on Mindfulness-Based Cognitive Therapy for the treatment of chronic and treatment-resistant depression.

KD declares no conflicts of interest.

ZS received book royalties from Guilford Press, workshop fees from the Centre for Mindfulness studies, and revenue from online sales at Mindful Noggin Inc., which are all related to his work as a co-founder of Mindfulness-Based Cognitive Therapy (MBCT).

MCM declares no conflicts of interest.

AS is the founder and director of the Radboudumc Expertise Centre for Mindfulness and co-investigator of a number of studies examining the effectiveness and implementation of mindfulness-based interventions for patients with psychological or somatic disorders and health care professionals.

AAF declares no conflicts of interest.

MGT declares no conflicts of interest.

JMM is associated with the University of Oxford Mindfulness Research Centre.

BDD is the lead of the University of Exeter AccEPT clinic, which offers courses of MBCT.

For peer review only - http://bmjopen.bmj.com/site/about/guidelines.xhtml

CS is a member of a training organisation that has been commissioned by NHS England to deliver MBCT training across NHS Talking Therapies services in England and co-leads the Sussex Mindfulness Centre. CS is a co-investigator of a programme grant evaluating an adapted MBCT course for adolescents experiencing depression and co-investigator on a grant evaluating an adapted MBCT course for NHS staff.

FAR regularly provides workshops on mindfulness-based interventions.

MR declares no conflicts of interest.

MH serves as a statistical consultant for HelloBetter/GetOn Institut für Gesundheitstrainings GmbH, a company that implements digital mental health therapeutics in routine care.

REFERENCES

1. Collaborators G 2019 MD. Global, regional, and national burden of 12 mental disorders in 204 countries and territories, 1990–2019: a systematic analysis for the Global Burden of Disease Study 2019. Lancet Psychiatry. 2022;9:137–50. https://doi.org/10.1016/s2215-0366(21)00395-3.

2. McIntyre RS, Alsuwaidan M, Baune BT, Berk M, Demyttenaere K, Goldberg JF, et al. Treatment-resistant depression: definition, prevalence, detection, management, and investigational interventions. World Psychiatry. 2023;22:394–412. https://doi.org/10.1002/wps.21120.

3. Rush AJ, Sackeim HA, Conway CR, Bunker MT, Hollon SD, Demyttenaere K, et al. Clinical research challenges posed by difficult-to-treat depression. Psychol Med. 2022;52:419–32. https://doi.org/10.1017/s0033291721004943.

4. McAllister-Williams RH, Arango C, Blier P, Demyttenaere K, Falkai P, Gorwood P, et al. The identification, assessment and management of difficult-to-treat depression: An international consensus statement. J Affect Disord. 2020;267:264–82. https://doi.org/10.1016/j.jad.2020.02.023.

5. Jaffe DH, Rive B, Denee TR. The humanistic and economic burden of treatment-resistant depression in Europe: a cross-sectional study. BMC Psychiatry. 2019;19:247. https://doi.org/10.1186/s12888-019-2222-4.

For peer review only - http://bmjopen.bmj.com/site/about/guidelines.xhtml

6. Bhattacharya R, Shen C, Sambamoorthi U. Excess risk of chronic physical conditions associated with depression and anxiety. BMC Psychiatry. 2014;14. https://doi.org/10.1186/1471-244x-14-10.

7. Moeller SB, Gbyl K, Hjorthøj C, Andreasen M, Austin SF, Buchholtz PE, et al. Treatment of difficult-to-treat depression – clinical guideline for selected interventions. Nord J Psychiatry. 2022;76:177–88. https://doi.org/10.1080/08039488.2021.1952303.

8. Markowitz JC, Wright JH, Peeters F, Thase ME, Kocsis JH, Sudak DM. The Neglected Role of Psychotherapy for Treatment-Resistant Depression. Am J Psychiatry. 2022;179:90–3. https://doi.org/10.1176/appi.ajp.2021.21050535.

9. McHugh RK, Whitton SW, Peckham AD, Welge JA, Otto MW. Patient Preference for Psychological vs Pharmacologic Treatment of Psychiatric Disorders: A Meta-Analytic Review. J Clin Psychiatry. 2013;74:595–602. https://doi.org/10.4088/jcp.12r07757.

10. Segal ZV, Williams JMG, Teasdale J. Mindfulness-Based Cognitive Therapy for Depression. 2013.

11. Barnhofer T, Dunn BD, Strauss C, Ruths FA, Barrett B, Ryan M, et al. Mindfulness-based cognitive therapy versus treatment as usual after non-remission with NHS Talking Therapies high-intensity psychological therapy for depression: a UK-based clinical effectiveness and cost-effectiveness randomised, controlled, superiority trial. Lancet Psychiatry. 2025;12:433–46. https://doi.org/10.1016/s2215-0366(25)00105-1.

12. Kuyken W, Warren FC, Taylor RS, Whalley B, Crane C, Bondolfi G, et al. Efficacy of Mindfulness-Based Cognitive Therapy in Prevention of Depressive Relapse: An Individual Patient Data Meta-analysis From Randomized Trials. Jama Psychiat. 2016;73:565. https://doi.org/10.1001/jamapsychiatry.2016.0076.

13. Ma SH, Teasdale JD. Mindfulness-Based Cognitive Therapy for Depression: Replication and Exploration of Differential Relapse Prevention Effects. J Consult Clin Psych. 2004;72:31–40. https://doi.org/10.1037/0022-006x.72.1.31.

14. Teasdale JD, Segal ZV, Williams JMG, Ridgeway VA, Soulsby JM, Lau MA. Prevention of relapse/recurrence in major depression by mindfulness-based cognitive therapy. J Consult Clin Psych. 2000;68:615–23. https://doi.org/10.1037/0022-006x.68.4.615.

15. Creswell JD, Lindsay EK. How Does Mindfulness Training Affect Health? A Mindfulness Stress Buffering Account. Current Directions in Psychological Science. 2014;23. https://doi.org/10.1177/0963721414547415.

16. Lindsay EK, Creswell DJ. Mechanisms of mindfulness training: Monitor and Acceptance Theory (MAT). Clinical Psychology Review. 2017;51. https://doi.org/10.1016/j.cpr.2016.10.011.

17. Eisendrath SJ, Delucchi K, Bitner R, Fenimore P, Smit M, McLane M. Mindfulness-Based Cognitive Therapy for Treatment-Resistant Depression: A Pilot Study. Psychother Psychosom. 2008;77:319–20. https://doi.org/10.1159/000142525.

For peer review only - http://bmjopen.bmj.com/site/about/guidelines.xhtml

18. Barnhofer T, Crane C, Hargus E, Amarasinghe M, Winder R, Williams JMG. Mindfulness-based cognitive therapy as a treatment for chronic depression: A preliminary study. Behav Res Ther. 2009;47:366–73. https://doi.org/10.1016/j.brat.2009.01.019.

19. Eisendrath SJ, Gillung E, Delucchi KL, Segal ZV, Nelson JC, McInnes LA, et al. A Randomized Controlled Trial of Mindfulness-Based Cognitive Therapy for Treatment-Resistant Depression. Psychother Psychosom. 2016;85:99–110. https://doi.org/10.1159/000442260.

20. Cladder-Micus MB, Speckens AEM, Vrijsen JN, Donders ART, Becker ES, Spijker J. Mindfulness-based cognitive therapy for patients with chronic, treatment-resistant depression: A pragmatic randomized controlled trial. Depress Anxiety. 2018;35:914–24. https://doi.org/10.1002/da.22788.

21. Michalak J, Schultze M, Heidenreich T, Schramm E. A randomized controlled trial on the efficacy of mindfulness-based cognitive therapy and a group version of cognitive behavioral analysis system of psychotherapy for chronically depressed patients. J Consult Clin Psych. 2015;83:951–63. https://doi.org/10.1037/ccp0000042.

22. Chiesa A, Castagner V, Andrisano C, Serretti A, Mandelli L, Porcelli S, et al. Mindfulness-based cognitive therapy vs. psycho-education for patients with major depression who did not achieve remission following antidepressant treatment. Psychiat Res. 2015;226:474–83. https://doi.org/10.1016/j.psychres.2015.02.003.

23. Garcia A, Yáñez AM, Bennasar-Veny M, Navarro C, Salva J, Ibarra O, et al. Efficacy of an adjuvant non-face-to-face multimodal lifestyle modification program for patients with treatment-resistant major depression: A randomized controlled trial. Psychiatry Res. 2023;319:114975. https://doi.org/10.1016/j.psychres.2022.114975.

24. Foroughi A, Sadeghi K, Parvizifard A, Moghadam AP, Davarinejad O, Farnia V, et al. The effectiveness of mindfulness-based cognitive therapy for reducing rumination and improving mindfulness and self-compassion in patients with treatment-resistant depression. Trends Psychiatry Psychotherapy. 2020;42:138–46. https://doi.org/10.1590/2237-6089-2019-0016.

25. Mathew T, Nordström K. Comparison of One-Step and Two-Step Meta-Analysis Models Using Individual Patient Data. Biom J. 2010;52:271–87. https://doi.org/10.1002/bimj.200900143.

26. Clarke MJ. Individual patient data meta-analyses. Best Pr Res Clin Obstet Gynaecol. 2005;19:47–55. https://doi.org/10.1016/j.bpobgyn.2004.10.011.

27. Riley RD, Lambert PC, Abo-Zaid G. Meta-analysis of individual participant data: rationale, conduct, and reporting. BMJ. 2010;340 feb05 1:c221. https://doi.org/10.1136/bmj.c221.

28. Jones AP, Riley RD, Williamson PR, Whitehead A. Meta-analysis of individual patient data versus aggregate data from longitudinal clinical trials. Clin Trials. 2009;6:16–27. https://doi.org/10.1177/1740774508100984.

For peer review only

29. Cooper H, Patall EA. The Relative Benefits of Meta-Analysis Conducted With Individual Participant Data Versus Aggregated Data. Psychol Methods. 2009;14:165–76. https://doi.org/10.1037/a0015565.

30. Stewart LA, Clarke M, Rovers M, Riley RD, Simmonds M, Stewart G, et al. Preferred Reporting Items for a Systematic Review and Meta-analysis of Individual Participant Data: The PRISMA-IPD Statement. Jama. 2015;313:1657–65. https://doi.org/10.1001/jama.2015.3656.

31. Löfholm CA, Brännström L, Olsson M, Hansson K. Treatment-as-usual in effectiveness studies: What is it and does it matter? Int J Soc Welf. 2013;22:25–34. https://doi.org/10.1111/j.1468-2397.2012.00870.x.

32. Kabat-Zinn J. Mindfulness-Based Interventions in Context: Past, Present, and Future. Clinical Psychology: Science and Practice. 2003;10:144–56. https://doi.org/10.1093/clipsy.bpg016.

33. Association AP. Diagnostic and statistical manual of mental disorders (5th ed.). Arlington, VA: Author; 2013.

34. Association AP. Diagnostic and statistical manual of mental disorders (4th ed.). Washington, DC: Author; 2000.

35. Korten NCM, Comijs HC, Lamers F, Penninx BWJH. Early and late onset depression in young and middle aged adults: Differential symptomatology, characteristics and risk factors? J Affect Disord. 2012;138:259–67. https://doi.org/10.1016/j.jad.2012.01.042.

36. Devanand DP, Adorno E, Cheng J, Burt T, Pelton GH, Roose SP, et al. Late onset dysthymic disorder and major depression differ from early onset dysthymic disorder and major depression in elderly outpatients. J Affect Disord. 2004;78:259–67. https://doi.org/10.1016/s0165-0327(02)00307-5.

37. Wahl I, Löwe B, Bjorner JB, Fischer F, Langs G, Voderholzer U, et al. Standardization of depression measurement: a common metric was developed for 11 self-report depression measures. J Clin Epidemiology. 2014;67:73–86. https://doi.org/10.1016/j.jclinepi.2013.04.019.

38. Rush AJ, Trivedi MH, Ibrahim HM, Carmody TJ, Arnow B, Klein DN, et al. The 16-Item quick inventory of depressive symptomatology (QIDS), clinician rating (QIDS-C), and self-report (QIDS-SR): a psychometric evaluation in patients with chronic major depression. Biol Psychiatry. 2003;54:573–83. https://doi.org/10.1016/s0006-3223(02)01866-8.

39. Kroenke K, Spitzer RL, Williams JBW. The PHQ-9: Validity of a brief depression severity measure. J Gen Intern Med. 2001;16:606–13. https://doi.org/10.1046/j.1525-1497.2001.016009606.x.

40. Löwe B, Unützer J, Callahan CM, Perkins AJ, Kroenke K. Monitoring Depression Treatment Outcomes With the Patient Health Questionnaire-9. Med Care. 2004;42:1194–201. https://doi.org/10.1097/00005650-200412000-00006.

For peer review only - http://bmjopen.bmj.com/site/about/guidelines.xhtml

41. Hudgens S, Floden L, Blackowicz M, Jamieson C, Popova V, Fedgchin M, et al. Meaningful Change in Depression Symptoms Assessed with the Patient Health Questionnaire (PHQ-9) and Montgomery-Åsberg Depression Rating Scale (MADRS) Among Patients with Treatment Resistant Depression in Two, Randomized, Double-blind, Active-controlled Trials of Esketamine Nasal Spray Combined With a New Oral Antidepressant. J Affect Disord. 2021;281:767–75. https://doi.org/10.1016/j.jad.2020.11.066.

42. Turkoz I, Alphs L, Singh J, Jamieson C, Daly E, Shawi M, et al. Clinically meaningful changes on depressive symptom measures and patient-reported outcomes in patients with treatment-resistant depression. Acta Psychiatr Scand. 2021;143:253–63. https://doi.org/10.1111/acps.13260.

43. Hölzel L, Härter M, Reese C, Kriston L. Risk factors for chronic depression — A systematic review. J Affect Disorders. 2011;129:1–13. https://doi.org/10.1016/j.jad.2010.03.025.

44. Seedat S, Scott KM, Angermeyer MC, Berglund P, Bromet EJ, Brugha TS, et al. Cross-National Associations Between Gender and Mental Disorders in the World Health Organization World Mental Health Surveys. Arch Gen Psychiatry. 2009;66:785–95. https://doi.org/10.1001/archgenpsychiatry.2009.36.

45. Buckman JEJ, Saunders R, Stott J, Arundell L-L, O'Driscoll C, Davies MR, et al. Role of age, gender and marital status in prognosis for adults with depression: An individual patient data meta-analysis. Epidemiology Psychiatr Sci. 2021;30:e42. https://doi.org/10.1017/s2045796021000342.

46. Trivedi MH, Rush AJ, Wisniewski SR, Nierenberg AA, Warden D, Ritz L, et al. Evaluation of Outcomes With Citalopram for Depression Using Measurement-Based Care in STARD: Implications for Clinical Practice. Am J Psychiatry. 2006;163:28–40. https://doi.org/10.1176/appi.ajp.163.1.28.

47. Oosten AJ van, Mens K van, Blonk RWB, Burdorf A, Tiemens B. The relationship between having a job and the outcome of brief therapy in patients with common mental disorders. BMC Psychiatry. 2023;23:910. https://doi.org/10.1186/s12888-023-05418-z.

48. Nanni V, Uher R, Danese A. Childhood Maltreatment Predicts Unfavorable Course of Illness and Treatment Outcome in Depression: A Meta-Analysis. Am J Psychiat. 2012;169:141–51. https://doi.org/10.1176/appi.ajp.2011.11020335.

49. Klein DN, Schatzberg AF, McCullough JP, Dowling F, Goodman D, Howland RH, et al. Age of onset in chronic major depression: relation to demographic and clinical variables, family history, and treatment response. J Affect Disord. 1999;55:149–57. https://doi.org/10.1016/s0165-0327(99)00020-8.

50. Bromberger JT, Schott L, Kravitz HM, Joffe H. Risk factors for major depression during midlife among a community sample of women with and without prior major depression: are they the same or different? Psychol Med. 2015;45:1653–64. https://doi.org/10.1017/s0033291714002773.

For peer review only - http://bmjopen.bmj.com/site/about/guidelines.xhtml

51. Myklebost SB, Gjestad R, Inal Y, Hammar Å, Nordgreen T. Predictors of Treatment Response to an Internet-Delivered Intervention Targeting Residual Cognitive Symptoms After Major Depressive Disorder. Front Psychiatry. 2022;13:795698. https://doi.org/10.3389/fpsyt.2022.795698.

52. Courtney DB, Watson P, Krause KR, Chan BWC, Bennett K, Gunlicks-Stoessel M, et al. Predictors, Moderators, and Mediators Associated With Treatment Outcome in Randomized Clinical Trials Among Adolescents With Depression. JAMA Netw Open. 2022;5:e2146331. https://doi.org/10.1001/jamanetworkopen.2021.46331.

53. Cuijpers P, Sijbrandij M, Koole SL, Andersson G, Beekman AT, Reynolds CF. Adding psychotherapy to antidepressant medication in depression and anxiety disorders: a meta-analysis. World Psychiatry. 2014;13:56–67. https://doi.org/10.1002/wps.20089.

54. DeRubeis RJ, Hollon SD, Amsterdam JD, Shelton RC, Young PR, Salomon RM, et al. Cognitive Therapy vs Medications in the Treatment of Moderate to Severe Depression. Arch Gen Psychiatry. 2005;62:409–16. https://doi.org/10.1001/archpsyc.62.4.409.

55. Button KS, Wiles NJ, Lewis G, Peters TJ, Kessler D. Factors associated with differential response to online cognitive behavioural therapy. Soc Psychiatry Psychiatr Epidemiology. 2012;47:827–33. https://doi.org/10.1007/s00127-011-0389-1.

56. Furlan AD, Pennick V, Bombardier C, Tulder M van, Group EB Cochrane Back Review. 2009 Updated Method Guidelines for Systematic Reviews in the Cochrane Back Review Group. Spine. 2009;34:1929–41. https://doi.org/10.1097/brs.0b013e3181b1c99f.

57. Sterne JAC, Savović J, Page MJ, Elbers RG, Blencowe NS, Boutron I, et al. RoB 2: a revised tool for assessing risk of bias in randomised trials. Bmj. 2019;366:l4898. https://doi.org/10.1136/bmj.l4898.

58. Lunn D, Jackson C, Best N, Thomas A, Spiegelhalter D. The BUGS Book, A Practical Introduction to Bayesian Analysis. 2019. https://doi.org/10.1201/b13613.

59. Goligher EC, Heath A, Harhay MO. Bayesian statistics for clinical research. Lancet. 2024;404:1067–76. https://doi.org/10.1016/s0140-6736(24)01295-9.

60. Higgins JPT, Thompson SG, Deeks JJ, Altman DG. Measuring inconsistency in meta-analyses. BMJ. 2003;327:557–60. https://doi.org/10.1136/bmj.327.7414.557.

61. Higgins JPT, Thompson SG. Quantifying heterogeneity in a meta-analysis. Stat Med. 2002;21:1539–58. https://doi.org/10.1002/sim.1186.

62. Harrer M, Cuijpers P, Furukawa TA, Ebert DD. Doing Meta-Analysis with R, A Hands-On Guide. 2022. https://doi.org/10.1201/9781003107347.

63. Harrer M. Heterogeneity Prior Justification. 2022. https://osf.io/3tfm6. Accessed 16 May 2025.

64. Furukawa TA, Leucht S. How to Obtain NNT from Cohen's d: Comparison of Two Methods. PLoS ONE. 2011;6:e19070. https://doi.org/10.1371/journal.pone.0019070.

For peer review only

65. Han S, Zhou X. Defining estimands in clinical trials: A unified procedure. Stat Med. 2023;42:1869–87. https://doi.org/10.1002/sim.9702.

66. Buuren van, Groothuis-Oudshoorn K. mice: multivariate imputation by chained equations in R. Journal of Statistical Software. 2011;45:1–67.

67. Resche-Rigon M, White IR. Multiple imputation by chained equations for systematically and sporadically missing multilevel data. Stat Methods Méd Res. 2018;27:1634–49. https://doi.org/10.1177/0962280216666564.

68. Zhou X, Reiter JP. A Note on Bayesian Inference After Multiple Imputation. Am Stat. 2010;64:159–63. https://doi.org/10.1198/tast.2010.09109.

69. Burgess S, White IR, Resche-Rigon M, Wood AM. Combining multiple imputation and meta-analysis with individual participant data. Stat Med. 2013;32:4499–514. https://doi.org/10.1002/sim.5844.

For peer review only

For peer review only - http://bmjopen.bmj.com/site/about/guidelines.xhtml

APPENDIX

Search Terms

Database	Search Term
Web of Science	(“MDE” OR “major depressive episode” OR “MDD” OR “major depressive disorder” OR “major depression” OR “depression” OR “dysthymia” OR “dysthymic”) AND (“chronic” OR “persistent” OR “treatment-resistant” OR “non-responders” OR “non-response” OR “non-remitted” OR “dysthymia” OR “dysthymic”) AND (“MBCT” OR "mindfulness-based cognitive therapy") AND (“randomized controlled trial” OR “randomised controlled trial” OR “randomized-controlled trial” OR “randomised-controlled trial” OR “RCT” OR “randomized” or “randomised” OR “randomly allocated” OR “randomly assigned”)
Scopus	(“MDE” OR “major depressive episode” OR “MDD” OR “major depressive disorder” OR “major depression” OR “depression” OR “dysthymia” OR “dysthymic”) AND (“chronic” OR “persistent” OR “treatment-resistant” OR “non-responders” OR “non-response” OR “non-remitted” OR “dysthymia” OR “dysthymic”) AND (“MBCT” OR "mindfulness-based cognitive therapy") AND (“randomized controlled trial” OR “randomised controlled trial” OR “randomized-controlled trial” OR “randomised-controlled trial” OR “RCT” OR “randomized” or “randomised” OR “randomly allocated” OR “randomly assigned”)
PubMed	(“MDE” OR “major depressive episode” OR “MDD” OR “major depressive disorder” OR “major depression” OR “depression” OR “dysthymia” OR “dysthymic”) AND (“chronic” OR “persistent” OR “treatment-resistant” OR “non-responders” OR “non-response” OR “non-remitted” OR “dysthymia” OR “dysthymic”) AND (“MBCT” OR "mindfulness-based cognitive therapy") AND (“randomized controlled trial” OR “randomised controlled trial” OR “randomized-controlled trial” OR “randomised-controlled trial” OR “RCT” OR “randomized” or “randomised” OR “randomly allocated” OR “randomly assigned”)
PsycInfo	(“MDE” OR “major depressive episode” OR “MDD” OR “major depressive disorder” OR “major depression” OR “depression” OR “dysthymia” OR “dysthymic”) **AND** (“chronic” OR “persistent” OR “treatment-resistant” OR “non-responders” OR “non-response” OR “non-remitted” OR “dysthymia” OR “dysthymic”) **AND** (“MBCT” OR "mindfulness-based cognitive therapy") **AND** (“randomized controlled trial” OR “randomised controlled trial” OR “randomized-controlled trial” OR “randomised-controlled trial” OR “RCT” OR “randomized” or “randomised” OR “randomly allocated” OR “randomly assigned”)
Embase	(‘MDE’ OR ‘major depressive episode’ OR ‘MDD’ OR ‘major depressive disorder’ OR ‘major depression’ OR ‘depression’ OR ‘dysthymia’ OR ‘dysthymic’) **AND** (‘chronic’ OR ‘persistent’ OR ‘treatment-resistant’ OR ‘non-responders’ OR ‘non-response’ OR ‘non-remitted’ OR ‘dysthymia’ OR ‘dysthymic’) **AND** (‘MBCT’ OR ‘mindfulness-based cognitive therapy’) **AND** (‘randomized controlled trial’ OR ‘randomised controlled trial’ OR ‘randomized-controlled trial’ OR ‘randomised-controlled trial’ OR ‘RCT’ OR ‘randomized’ or ‘randomised’ OR ‘randomly allocated’ OR ‘randomly assigned’)

For peer review only - http://bmjopen.bmj.com/site/about/guidelines.xhtml

Cochrane Controlled Trials	(“MDE” OR “major depressive episode” OR “MDD” OR “major depressive disorder” OR “major depression” OR “depression” OR “dysthymia” OR “dysthymic”) AND (“chronic” OR “persistent” OR “treatment-resistant” OR “non-responders” OR “non-response” OR “non-remitted” OR “dysthymia” OR “dysthymic”) AND (“MBCT” OR "mindfulness-based cognitive therapy") AND (“randomized controlled trial” OR “randomised controlled trial” OR “randomized-controlled trial” OR “randomised-controlled trial” OR “RCT” OR “randomized” or “randomised” OR “randomly allocated” OR “randomly assigned”)

For peer review only
